# Supplementary figures and images for: Acceptability and feasibility of weight management programmes for adults with severe obesity: a qualitative systematic review
Source: BMJ Open. 2019 Sep 11;9(9):e029473. doi: 10.1136/bmjopen-2019-029473 (PMC6738728; doi:10.1136/bmjopen-2019-029473)

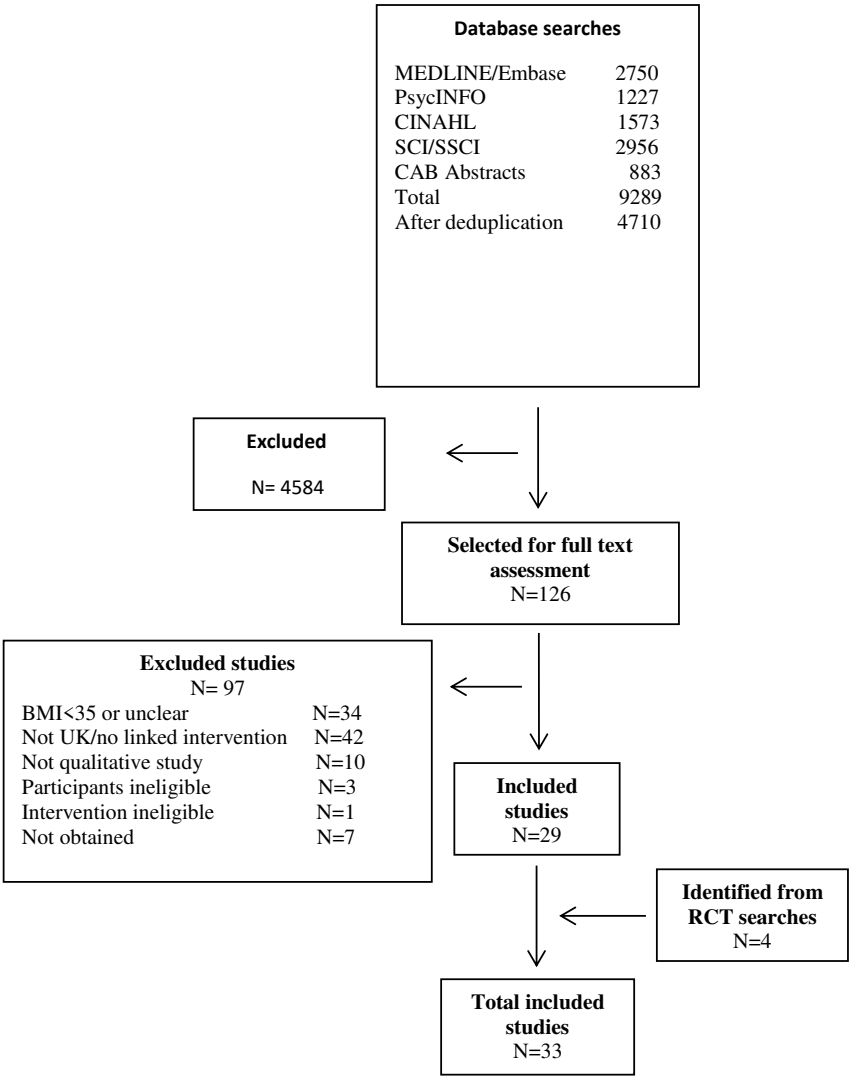

S1 Figure Flow chart of included studies

Supplement: Supplementary data [file bmjopen-2019-029473supp004.pdf]

Motivating factors for engagement

Generally positively valued aspects of WMPs

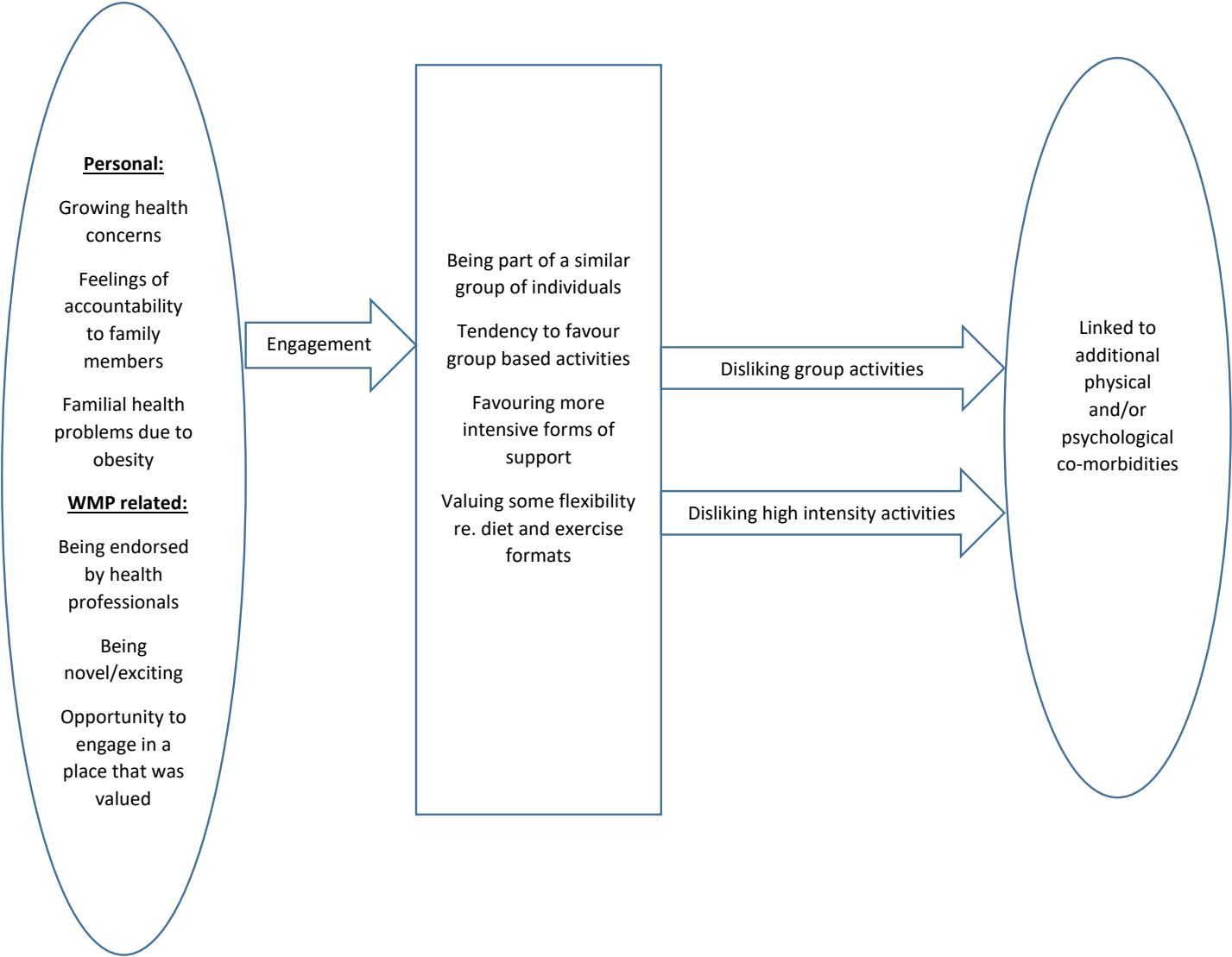

Supplement: Supplementary data [file bmjopen-2019-029473supp001.pdf]
