# Supplementary material for: Acceptability and feasibility of weight management programmes for adults with severe obesity: a qualitative systematic review
Source: BMJ Open. 2019 Sep 11;9(9):e029473. doi: 10.1136/bmjopen-2019-029473 (PMC6738728; doi:10.1136/bmjopen-2019-029473)
Supplement: Supplementary data [file bmjopen-2019-029473supp005.pdf]

S1 Table Characteristics of the included qualitative studies

Study	Aim (as described within the papers)	Condition of Focus	Participants Characteristics	Details of intervention	Qualitative data collection methods
First Author: Bennett<br>*Year:* 2014<br>*Category*: A<br>*Country*: USA	To understand primary care providers' (PCPs) perspectives about their role in the intervention and in their patients' weight loss, thereby providing insights to inform best practices in developing practice-based weight management programmes.	Patients with obesity in their usual care practices.	*Role:* Provider<br>*Number providers interviewed*: 26 PCPs<br>*Providers' characteristics*: 15 female, 11 male, 24 physicians, 2 nurse practitioners, and 20 had internal medicine training. The mean time in practice was 16 years (SD ± 11.7), and mean number of patients in the trial was 11.1 (SD ± 6.8)<br>*Socioeconomic and demographic characteristics*: 15 White, 6 Asian/Pacific Islander, 3 Black, 2 Other	The Practice-based Opportunities for Weight Reduction (POWER) was a 24 month trial that had two intervention groups (by phone and face-to-face) in which weight-loss health coaches (not PCPs) provided education and positive reinforcement. Participants in both intervention arms had access to the same online educational modules, self-monitoring tools and received both automated and individualized e-mails. Participants in the control arm met with a weight loss health coach at the time of randomization and, if desired, after the final data collection visit. They also received brochures along with a list of recommended weight loss websites.	Focus groups
First Author: Bradbury<br>*Year:* 2015<br>*Category*: A	To explore helpful (and unhelpful) aspects of coaching;	Participants with obesity.	*Role:* Participant<br>*Number of participants*: 58.	Positive Online Weight Reduction (POWeR) is an e-health intervention designed to produce sustainable weight	Interviews

Country: UK	the experiences of POWeR and the accompanying coaching, including what aspects people found most helpful, unhelpful, appealing or unappealing, and what factors seemed to influence whether participants continued to follow POWeR.		Planning and development stages: 16 participants;<br>Feasibility stage: 23 participants;<br>Community trial 19 participants.<br>*Participants' characteristics*: From the community trial: age range 34-68, Participants were sampled from both the coaching arm (10 female, four male) and Web only arm (four female, one male) and varied in their usage of POWeR.<br>*Socioeconomic and demographic characteristics*: NR<br>*Comorbidities*: NR	management. POWeR consisted of 12 sessions which taught users self-regulation skills in order for them to become their own personal health trainer. Patients were randomized to either usual care, the POWeR website, POWeR accompanied by basic nurse support, or POWeR with regular nurse support. The nurse support was mainly delivered face to face, although telephone and email support could also be provided.	
First Author: Gudzune<br>*Year:* 2012<br>*Category*: A<br>*Country*: USA	To explore PCPs' usual practices as part of weight counselling to identify how PCPs communicate with their patients about weight loss.	Patients with obesity in their usual care practices	See *Bennett 2014*	See *Bennett 2014*	Focus groups
First Author: Hunt<br>*Year:* 2014<br>*Category*: A<br>*Country*: UK	To report the characteristics of men participating in a randomised	Men with obesity (BMI > 28kg/m^2), age 35–65 at high	*Role:* Participant<br>*Number of participants*: 63 men (who had attended at least six FFIT sessions of the programme).	Football Fans in Training (FFIT) is a men-only, evidence-based, 12-session, weight management and physical activity group programme with subsequent	Focus groups

	controlled trial of a weight management programme designed specifically to attract men, and, secondly, their accounts of why they decided to participate in the programme.	risk of ill-health due to obesity	*Participants characteristics*: No specific data for qualitative analysed participants<br>*Socioeconomic and demographic characteristics*: NR<br>*Comorbidities reported*: NR	minimal-contact weight loss maintenance support delivered free of charge at Scotland's top professional football clubs by community coaches trained in diet, nutrition, physical activity and behaviour change techniques to a standard programme delivery protocol.	
First Author: Little<br>*Year:* 2017<br>*Category*: A<br>*Country*: UK	To explore patients' expectations of POWeR+, experiences of the POWeR+ programme, experiences of using the POWeR+ website and experiences of nurse support.	Participants with obesity (BMI ≥30kg/m^2, or ≥28kg/m^2 with comorbidities) from general practice	*Role:* Participant and Provider<br>*Number of providers*: 13 nurses (HCPs who supported POWeR+ were included in qualitative evaluation)<br>*Number of participants:* 31 POWeR+ programme users. 14 remote support (3 low users/11 high users) and 17 face-to-face support patients (2 low users/15 high users).<br>*Participants' characteristics*: 15 female, 16 male, mean age 61 years (range 45-88 years).<br>*Socioeconomic and demographic characteristics*: No specific data for qualitative analysed participants.<br>*Comorbidities reported*: No specific	This is a 24-session web-based weight management intervention consisting of a series of 24 brief maintenance-oriented sessions for up to 6 months and links to encourage patients to continue to use the website to track their weight at least fortnightly until they have formed healthy eating habits that sustain weight management.	Interviews

			data for qualitative analysed participants.		
First Author: McRobbie<br>*Year:* 2016<br>*Category*: A<br>*Country*: UK	To explore the many components of the WAP. By providing a summary of participant feedback on the overall helpfulness of the programme.	Adults (aged ≥ 18 years) with obesity (BMI of ≥ 30 kg/m^2 or a BMI of ≥ 28 kg/m^2 plus comorbidities) who wanted to lose weight	*Role:* Participant<br>*Number of participants:* 177. Participants who reported helpfulness of the programme at 12-months follow up; 48 in the nurse arm and 129 in the WAP arm. People who dropped out of treatment were called; only 19 provided a reason for dropping out.<br>*Participants' characteristics*: Not reported<br>*Socioeconomic and demographic characteristics:* Not reported.<br>*Comorbidities:* Not reported	The WAP is a multicomponent programme that includes a range of concrete and verifiable tasks agreed individually with each participant and also includes monthly 'maintenance' sessions that targeted to improve participant motivation, allowing participants to discuss the challenges they have faced since the last session, and to anticipate challenges of the month ahead.	Anonymous feedback questionnaire
First Author: Yarborough<br>*Year:* 2016<br>*Category*: A<br>*Country*: USA	To assess lifestyle change barriers and facilitators across the first 18 months of study participation and to identify modifiable factors associated with making and maintaining healthy	Adults (aged ≥ 18 years) with obesity (BMI ≥27kg/m^2) taking antipsychotic medications (stable on antipsychotic medications for at least 30 days)	*Role:* Participant<br>*Number of participants*: 84. Participants in the control arm were interviewed once; 17 intervention participants were interviewed more than once to ensure that all cohorts were represented in each interview wave.<br>*Participants' characteristics*: Mean age 48.1 (SD ± 10.1), 30 male, 54	This was a 24-month study of the STRIDE comprehensive weight loss and lifestyle-change intervention that consisted of 24 weekly meetings that targeted readiness to change; included interactive, participant-centred delivery of lifestyle education information along with a 20-min walk; encouraged skills practice, self-monitoring and feedback; and facilitated group interactions and	Interviews

	lifestyle changes in order to inform clinicians and improve the development of future interventions for individuals with serious mental illnesses.		female. 18 were members of ethnic or racial minorities.<br>*Socioeconomic and demographic characteristics*: 34 married or living with partner, 27 had an income of $30,000 or higher, 18 were college graduate or higher, 28 were retired, unemployed, student, homemaker or temporarily laid off.<br>*Comorbidities*: 34 Schizophrenia, 17 bipolar disorder, 31 affective psychoses, 2 PTSD	support. Intervention participants could consult with interventionists by telephone as needed.	
First Author: Abildso<br>*Year:* 2010<br>*Category*: B<br>*Country*: USA	To examine physical and psychosocial differences at baseline between completers of and dropouts from a 12-week weight management program; to assess the physical, behavioural, and psychosocial impact on program completers; to	Adults with obesity (BMI ≥ 30kg/m^2 alone or a BMI of 25 to 29.9kg/m^2 with comorbidities)	*Role:* Participant<br>*Number of participants*: 11<br>*Participants characteristics*: Mean age 46.2 (SD ± 8.5), 8 female, 3 male. Seven were successful program completers (three high weight losers, four moderate weight losers), and four were program dropouts or completers with low weight loss).<br>*Socioeconomic and demographic characteristics*: 7 married, number of children 1.5 (SD ± 1.1)<br>*Comorbidities*: Not reported	Weight loss is encouraged in the weight management program (WMP) through increasing physical activity and decreasing caloric intake. For a $45 monthly co-payment, the WMP benefit during Phase 1 (12 weeks) included assessment and follow-up meetings with an exercise physiologist and registered dietitian, monthly personal training sessions, and periodic phone calls from the insurance agency to track progress.	Interviews

	compare the psychosocial changes of high and moderate weight losers; and to qualitatively explore factors associated with program adherence and weight loss.				
First Author: Aschbrenner<br>*Year:* 2016<br>*Category*: B<br>*Country*: USA	To explore participants' perceptions and experiences with peer interactions during the lifestyle intervention.	Obese (BMI ≥ 30kg/m^2) adults (aged 21 or older) with serious mental illness (diagnosis of schizophrenia, schizoaffective disorder, major depressive disorder, or bipolar disorder) on stable pharmacological treatment	*Role:* Participant<br>*Number of participants*: 17<br>*Participants' characteristics*: No specific data for qualitative analysed participants<br>*Socioeconomic and demographic characteristics*: Not reported<br>*Comorbidities:* Not reported	A 24-week group-based lifestyle intervention that consisted of once weekly 1-hr group weight management sessions facilitated by a psychologist and a public health professional; twice weekly (optional) 1-hr group exercise sessions led by a certified fitness trainer; and mobile technology and use of social media to increase motivation and facilitate self-monitoring and peer-to-peer support outside of in person group treatment or exercise sessions.	Focus groups

First Author: Asselin<br>*Year:* 2015<br>*Category*: B<br>*Country*: Canada	To explore how primary care providers incorporate weight management in their practice.	Obesity prevention and weight management at interdisciplinary primary care environment	*Role:* Provider<br>*Number of providers interviewed:* 29<br>*Providers' characteristics*: 7 mental healthcare workers, 7 registered dietitians, 15 registered nurses or nurse practitioners.<br>*Socioeconomic and demographic characteristics:* NR	The 5 As Team (5AsT) study was designed to create, implement and evaluate a flexible intervention to improve the quality and quantity of weight management visits in primary care. 5AsT is a randomized controlled trial on the implementation of a 6-month 5AsT intervention designed to operationalize the 5As of obesity management in primary care.	Interviews and field notes of intervention sessions
First Author: Asselin<br>*Year:* 2016<br>*Category*: B<br>*Country*: Canada	To describe the intervention, provide continual intervention monitoring and to identify contextual factors that could influence the primary outcome measure.	See *Asselin 2015*	See *Asselin 2015*	See *Asselin 2015*	See *Asselin 2015*
First Author: Barham<br>*Year:* 2011<br>*Category*: B<br>*Country*: USA	To improve nutrition and physical activity of county employees and promote weight loss (*There was no*	Adults at highest risk for the development of diabetes or who already have been	*Role:* Participant<br>*Number of participants*: Unclear how many of 45 programme participants provided written responses on the end of study programme evaluations.	There were 2 waves of enrolment and 4 intervention groups (up to 12 participants/ group). The intervention was a 3-month program (12 one hour weekly midday group sessions) that targeted healthy diet, physical activity,	Written responses to end of programme participant evaluations

	qualitative aim stated).	diagnosed with type 2 diabetes	*Participants characteristics*: No specific data for those who provided written responses<br>*Socioeconomic and demographic characteristics*: Not reported<br>*Comorbidities reported*: Not reported	and stress reduction, followed by a monthly maintenance program with the groups choosing topics that they considered of greatest benefit. Most of the sessions were led by a nurse educator, but individual sessions were also conducted by a dietitian, psychologist, and physical therapist all employees of Upstate Medical University, Syracuse, NY.	
First Author: Borkoles<br>*Year:* 2016<br>*Category*: B<br>*Country*: UK	To examine the effects of a non-dieting lifestyle intervention approach for women with morbid obesity designed in the framework of the self-determination theory and Health at Every Size on weight maintenance and psychological functioning.	Pre-menopausal females with morbid obesity (BMI ≥30kg/m^2) older than 18 years of age free of obesity-related diseases and fit for exercise	*Role:* Participant<br>*Number of participants*: 62 (62 interviews at baseline with 36 follow-up interviews, including 12 drop-outs).<br>*Participants' characteristics*: Pre-menopausal women predominantly white Caucasian (97%), with a mean age of 40.2 years<br>*Socioeconomic and demographic characteristics*: most were from the lower SES background, 21% had a degree and 57% left school at 16, 66.1% worked full time and 11% worked part-time, in mainly manual	The WHEEL (Weight, Healthy Eating and Exercise in Leeds) study was a delayed-start, 12 weeks of intensive intervention and 40-week maintenance phase RCT comprising of community-based supervised exercise, lifestyle physical activity and psycho-educational classes on healthy eating and weight management.	Interviews

			(29%) and administrative jobs (46.8%)<br>*Comorbidities*: 50% met the International Diabetes Federation metabolic syndrome criteria, 42% reported to have depression often or very often, and 36% used medication related to psychological problems		
First Author: Dahl<br>*Year:* 2014<br>*Category*: B<br>*Country*: Norway	To describe how personnel argued for and perceived a residential weight-loss program, to investigate how the participants experienced the program, and to contrast these perspectives.	Adults (between 18 and 60 years old) with obesity (BMI > 40kg/m^2 or >35kg/m^2 including comorbidities)<br>*Providers*:<br>The personnel were recruited among the staff at the centre	*Role:* Participant and Provider<br>*Number of participants*: 10<br>*Participants' characteristics*: 10 Norwegian participants took part in interviews (8 in focus groups and 2 individually). The age and weight range for these 10 persons were the same as for the total sample (n=30). Age between 22 and 56 years old, their BMI was between 40 and 63, and the group's mean body weight was 144kg<br>*Socioeconomic and demographic characteristics*: NR<br>*Comorbidities:* NR<br>*Number of providers interviewed*: 6<br>*Providers' characteristics*: 2 males and 4 females, considered to be key	This 18-week on-site program intervention took place at the Danish residential weight-loss centre. The program consisted of group-based intensive structured group exercise and educational sessions exercise, diet (individual calorie intake was based on energy calculations for a normal weight person with a sedentary activity level), and an educational program. The educational program comprised lessons about nutrition, monitoring of food intake and instruction in behavioural techniques from cognitive therapy. The personal development component included a minimum of two individual conversations with one of the	Focus groups and interviews

			personnel; the director, the administrative executive, and the leaders of the main areas diet, exercise and personal development	psychotherapists, motivational meetings for all participants.	
First Author: Danielsen<br>*Year:* 2016<br>*Category*: B<br>*Country*: Norway	To explore the experiences of physical activity from a participant perspective prior to, during, and after an intensive inpatient lifestyle modification program, including a high volume of adapted physical activity for the treatment of severe obesity.	Both genders, with a variety in age, degree of obesity (BMI ≥ 40 or 35.0–39.9 with comorbidities), and weight loss during the inpatient stay, as well as variation in weight-loss maintenance and lack of maintenance	*Role:* Participant<br>*Number of participants*: 8<br>*Participants' characteristics*: 5 female, 3 male, aged 35 to 63 years; 6 married/cohabitants and 2 single; BMI ranged from 37 to 60 and body weight from 96 to 185 kg<br>*Socioeconomic and demographic characteristics*: NR<br>*Co-morbidities:* NR	The study was supplementary to a clinical controlled trial with a 1-year prospective follow-up study examining the effects of a 10- to 14-week inpatient lifestyle modification program for subjects with severe obesity. Two to three group-exercise sessions 5 days a week during the inpatient period, each lasting for a minimum of 45 minutes. Aiming to increase compliance, the activity was supervised by exercise scientists and physiotherapists, and the participants were introduced to adapted physical activity and equipment, and exercised together with other individuals with severe obesity.	Interviews
First Author: Groven<br>*Year:* 2010<br>*Category*: B<br>*Country*: Norway	To show how the training is experienced from a first-person perspective, namely	Female participants with obesity (BMI >35kg/m^2) from the weight-loss program in	*Role:* Participants<br>*Number of participants*: 5<br>*Participants' characteristics*: Aged 35-63 years and had been overweight for more than 10 years	Group-based weight-loss program in Norway, a program organized by physiotherapists in the primary health system. Offered to eight women struggling with obesity problems in a particular district of Norway for one	Interviews

	the patients themselves.	Norway	*Socioeconomic and demographic characteristics*: 3 married, 1 divorced and 1 widowed, 1 had a university degree, 2 had a college degree, and 2 had no formal education after high school. The women were at present or previously working in professions providing a service, or care, doing office work, or an academic job on various levels.<br>*Comorbidities*: Not reported	year. Total of 12 exercises were performed throughout the one-hour exercise program. The treatment also included group discussion for 1 hour per month.	
First Author:<br>Jackson<br>Year: 2007<br>Category: B<br>Country:<br>UK	To evaluate the effectiveness and acceptability of a specialist health visitor-led weight management clinic in primary care.	Patients with a BMI ≥30	*Role: Participants*<br>*Number of participants: Unclear how many of 25 questionnaires returned provided written responses*<br>*Participants' characteristics: Not reported*<br>*Socioeconomic and demographic characteristics: Not reported*<br>*Comorbidities: Not reported*	Specialist health visitor-led intervention based on the Jan Felgens '12E2' model. The specialist health visitor sought to inspire participants through a combination of shared goal setting, reflection, problem-solving, positive affirmation and reinforcement. Consultations took place at the health centre and a relaxed, unhurried atmosphere was created. The average consultation time was 20 minutes (range 10–30 minutes), although the first appointment took approximately 1 hour and gave participants time to reflect on their lifestyles and to plan realistic goals	Open ended response options to questionnaire

				for healthy eating and physical activity with the specialist health visitor.	
First Author: Janke<br>*Year:* 2012<br>*Category*: B<br>*Country*: USA	To gain insight into the patient's experience of comorbid chronic pain and obesity and to improve understanding of the behavioural linkages between the experience of pain, engagement in health behaviours, and obesity treatment outcomes.	Patients attending primary care clinics at a large Midwestern Veteran's Affairs hospital, > 18 years, BMI ≥25; weekly pain at an intensity ≥4 during the prior 3 months; and current diagnosis of a medical complaint associated with persistent pain	*Role:* Participant<br>*Number of participants*: 30<br>*Participants characteristics*: 24 male, 6 female<br>26 were age 50 or older, mean BMI was 36.8 (SD ± 8.9)<br>*Socioeconomic and demographic characteristics*: 22 were white, 20 had greater than a high school education, and 14 were unemployed or disabled while 13 were retired<br>*Comorbidities*: Measured on a scale of 0 to 10 (0 = none, 10 = worst imaginable), average pain intensity was 5.6 (SD ± 1.9) and average pain interference was 3.6 (SD ± 2.1)	The qualitative research project was designed to identify perceptions of those with both overweight/obesity and chronic pain regarding their experience of the course, impact, and treatment history of pain and weight symptoms; factors that might either ease or limit their ability to engage in health-promoting behaviours; and factors that facilitate or hinder engagement in treatments designed to achieve weight and/or pain control.	Focus groups and interviews
First Author: Jennings<br>*Year:* 2014<br>*Category*: B<br>*Country*: UK	To facilitate weight loss by implementing progressive and sustainable lifestyle changes, based on individually agreed goals over a 1-year	Adults (over 18 years) with obesity (BMI ≥40, or BMI ≥30 with obesity-related comorbidities and/or waist	*Role:* Participant<br>*Number of participants*: 12<br>*Participants' characteristics*: No specific data for qualitative analysed participants<br>*Socioeconomic and demographic characteristics*: No specific data for qualitative analysed participants.	The Fakenham weight management service (FWMS) provides Tier 3 services. This paper was service evaluation and had a cohort design recruited patients to a 1-year programme.	Focus groups

	programme. Focus groups were conducted to explore participants' experiences.	circumference ≥102 cm in men or ≥88 cm in women)	*Comorbidities*: No specific data for qualitative analysed participants.		
First Author: Jimenez Lopez<br>*Year:* 2012<br>*Category*: B<br>*Country*: Mexico	To explore the motivations of patients involved in a with reduction programme, by analysing their experiences.	Patients with obesity included in a waiting list for bariatric surgery at a public hospital	*Role:* Participant<br>*Number of participants*: 10<br>*Participants' characteristics*: 2 Male, 8 women, mean age 45.2, mean BMI 41.3<br>*Socioeconomic and demographic characteristics*: NR<br>*Comorbidities*: NR	The dynamic of the intervention included the modification of dietary habits by a psychologic intervention, as recommended by the federal law of obesity management The focus group included ten patients with one investigator as an active observer, and 12 weekly sessions.	Focus groups
First Author: Kidd<br>*Year:* 2013<br>*Category*: B<br>*Country*: USA	To describe the effect of an 8-week mindful eating intervention on mindful eating, weight loss self-efficacy, depression, and biomarkers of weight in urban, underserved, women	Females (aged 30 years and older) with obesity (BMI ≥30kg/m^2)	*Role:* Participant<br>*Number of participants*:12<br>*Participants' characteristics*: Mean weight was 119.7kg (SD ± 16.87), BMI 44.7 (SD ±6.9)，Age ranged from 31–61 and averaged 51.8 years (SD ± 9.1)<br>*Socioeconomic and demographic characteristics*: 7 African American, 5 unemployed, and 4 married; 11	The study used a mixed methods design. A one group pre-test/ post-test design examined the effect of an 8-week mindful eating intervention on the psychosocial variables and biomarkers. Weekly group sessions lasted 60 to 90 minutes and consisted of education and application of mindful eating principles.	Focus groups

	with obesity; and to identify themes of the lived experience of mindful eating.		graduated from high school, 6 had college degrees<br>*Comorbidities*: Not reported		
First Author: Pera<br>*Year:* 2016<br>*Category*: B<br>*Country*: Spain	To explore the meaning of obesity in elderly persons with knee osteoarthritis and to determine the factors that encourage or discourage weight loss.	Participants with obesity, knee osteoarthritis, and polypathology	*Role:* Participant<br>*Number of participants*: 10<br>*Participants characteristics*: 2 male, 8 female, mean age 67.23 (SD ±7.87), BMI 40.47 (SD ± 4.22), mean weight 92.35 kg (SD ± 8.93)<br>*Socioeconomic characteristics*:: 1 No education, 5 Primary (<5 years), 3 Secondary (<10 years), 1 Higher (>10 years), 2 Housewife, 8 Retired<br>*Comorbidities*: Mean number of co-morbidities 7.02 (SD ± 3.08)	The therapeutic education and functional preadaptation program was a 4-month program consisted of two 40-minute individual visits and three 90-minute group sessions for participants with obesity, knee osteoarthritis and polypathology. The program was designed following the methodology established for this type of program and was based on social learning theories.	Focus group
First Author: Counterweight<br>*Year:* 2008<br>*Category*: B<br>*Country*: UK	To explore key barriers and facilitators of practice and patient engagement in the Counterweight Programme and to describe key strategies used to	Patients with obesity in routine primary care	*Role:* Participant and Provider<br>*Number of participants*: 37 patients<br>*Number of providers*: weight management advisers (n = 7) in a focus group. In depth interviews were conducted with 15 PNs and 7 GPs across 11 practices.<br>*Participants' and/or providers characteristics*: Not reported	The Counterweight Project was set up to establish and improve obesity management in primary care by implementing an evidence-based weight management intervention that is practice focused. It was developed using theoretical models of behavioural change and, the best available methods from the published evidence.	*Participants*: Interviews and focus groups<br><br>*Providers*: Interviews and focus groups

	address barriers in the wider implementation of this weight management programme in UK primary care.		*Socioeconomic and demographic characteristics*: Not reported<br>*Comorbidities reported*: Not reported		
First Author: Shaw<br>*Year:* 2013<br>*Category*: B<br>*Country*: USA	To evaluate the acceptability, feasibility, and efficacy of daily text messages using regulatory focus theory to help individuals sustain weight loss.	Individuals had to own a mobile phone, be able to receive text messages, and have lost 5% of their body weight since entering the Duke Diet and Fitness Centre	*Role:* Participant<br>*Number of participants*: 60<br>*Participants' characteristics*: No specific data for qualitative analysed participants<br>*Socioeconomic and demographic characteristics*: No specific data for qualitative analysed participants.<br>*Comorbidities*: Not reported	Clients who received treatment at a residential weight loss management program that provides education, practical behavioural strategies, and ongoing support to make long-term changes at the Duke Diet and Fitness Centre (DFC), participated in this study. Participants were randomized to a promotion, prevention, or an attention control text message group after completion of a weight loss program.	Interviews
First Author: Sturgiss<br>*Year:* 2016<br>*Category*: B<br>*Country*: Australia	To describe the collaborative process used to develop an obesity management programme based on current Australian guidelines for GPs and their patients to	Health professionals involved in obesity management programme based on current Australian	*Role:* Provider<br>*Number of providers*: 38<br>*Providers' characteristics*: 15 GPs, 14 GPs registrar, 5 healthcare consumer representative, 2 representative bodies for chronic illness, 1 dietician, 1 psychologist	The Change Programme is a GP-delivered weight management programme that was developed based on Australian guidelines for the management of obesity in primary healthcare. It is based on one of the pillars of general practice—'patient centeredness'. No directive patient goals	Interviews and focus groups

	be used in primary care.	guidelines for GPs and their patients to be used in primary care	*Socioeconomic and demographic characteristics*: Not reported	were stated and the work was individualized. The programme consists of a GP handbook, patient workbook and computer template. This programme. The patients initially attended appointments every 2 weeks, with less frequent appointments as the programme continued.	
First Author: Sturgiss<br>*Year:* 2017<br>*Category*: B<br>*Country*: Australia	To assess the acceptability and feasibility of a GP-delivered weight management programme.	*Providers*: Fully qualified GPs from the Australian Capital Territory and New South Wales.	*Role:* Participant and Provider<br>*Number of providers*: 12<br>*Providers' characteristics*: The recruited GPs had an average 12 years of experience (range 4–30 years). The GPs worked in four urban practices and one rural practice.<br>*Number of patient participants*: 15 interviewed<br>*Participants' characteristics*: No specific data for qualitative analysed participants.<br>*Socioeconomic and demographic characteristics*: NR<br>*Comorbidities*: Not reported	See *Sturgiss 2016a*	Interviews
First Author: Sturgiss<br>*Year:* 2017<br>*Category*: B	To assess the self-efficacy and confidence of GPs	GPs working in 5 different general practices	*Role:* Provider<br>*Number of providers*: 12	See *Sturgiss 2016a*	Interviews

Country: Australia	before and after implementing a weight management programme in their practice.		*Providers' characteristics*: 12 GPs practised in 5 different general practices, 1 rural and 4 urban, and had between 4 and 30 years clinical experience<br>*Socioeconomic and demographic characteristics*: Not reported		
First Author: Turner<br>*Year:* 2015<br>*Category*: B<br>*Country*: UK	To determine both physiological benefits and qualitative information, namely patient satisfaction, associated with the service.	Patients with obesity attending Multidisciplinary Weight Management Clinic (MDWMC) at Aneurin Bevan Hospital, Wales	*Role:* Participant<br>*Number of participants*: 180<br>*Participants characteristics*: 131 female, 49 male, ages ranged between 19 and 74<br>*Socioeconomic and demographic characteristics*: Not reported<br>*Comorbidities*: Not reported	Obesity management in Wales includes the provision of a 1:1 MDWMC. Strategic management of obesity in Wales is guided by The All Wales Obesity Pathway and recommends MDWMCs for people with obesity who have one or more co morbidities and who have tried several interventions without success, or who have complex emotional relationships with food.	Interviews
First Author: VanWormer<br>*Year:* 2010<br>*Category*: B<br>*Country*: USA	To examine the association between participant and program experiences and satisfaction with a weight loss intervention.	Adults (18 years or older) with obesity (BMI ≥ 32kg/m^2) employees of a managed care organization	*Role:* Participant<br>*Number of participants*: 78 (*not clear if all of these provided qualitative information*)<br>*Participants' characteristics*: Mean age 46.9 (SD ± 8.3), 70 female, 8 male, 55 married or living with a partner, 23 not married; body weight	Participants were randomly assigned to either an immediate or delayed start group. The intervention lasted 6 months. During treatment, participants received a telephone-based behavioural weight loss counselling intervention. The intervention included a course manual, behaviour change tools (e.g., food/ activity log, weight chart, pedometer),	Written responses to open ended response options within a questionnaire

			(kg) 106.2 (SD ± 16.32), BMI 38.3 (SD ± 5.2)<br>*Socioeconomic and demographic characteristics*: 36 college or graduate degree, 42 had less than college degree<br>*Comorbidities*: Not reported	and up to 10 telephone counselling calls from a registered dietitian and/or health educator. In addition, participants received a home tele monitoring scale and were instructed to weigh themselves daily.	
First Author: Young<br>*Year:* 2017<br>*Category*: B<br>*Country*: USA	To determine whether computerized provision of weight management with peer coaching is feasible to deliver, is acceptable to patients, and is more effective than in-person delivery or usual care.	Adults (18 years or older) with obesity (BMI > 30 or 28–30kg/m^2 with self-reported weight gain of at least 10 pounds in the last 3 months), with diagnosis of schizophrenia, schizoaffective disorder, bipolar disorder, major depressive disorder with psychosis, or posttraumatic	*Role:* Participant<br>*Number of participants*: 48 (24 randomized to WebMOVE and 24 randomized to MOVE SMI)<br>*Participants' characteristics*: No specific data for qualitative analysed participants<br>*Socioeconomic and demographic characteristics*: No specific data for qualitative analysed participants<br>*Comorbidities*: Not reported	Patients were randomized to a computerized weight management with peer coaching (Web- MOVE) or in-person clinician-led weight services, or usual care. Both active interventions offered the same educational content. WebMOVE weekly manualized peer coaching was delivered by phone and emphasized a strengths-based approach with motivational interviewing. MOVE SMI is an in-person weight management program led by a master's level mental health clinician. The program includes 24 sessions (8 individual and 16 group), each lasting 60 min. Usual care consisted of one educational handout on the benefits of weight loss, given to participants after randomization	Interviews

		stress disorder; with prescribed an antipsychotic medication			
First Author: Zizzi<br>*Year:* 2016<br>*Category*: B<br>*Country*: USA	To explain how these services are perceived and received by participants in a community-based intervention so that specific recommendations can be made to health professionals working with similar populations and in similar settings.	West Virginia public employees' insurance agency weight management program (WMP), which is open to insured members that have a BMI >25	*Role:* Participant<br>*Number of participants*: 567 (not clear how many provided qualitative data within the questionnaire<br>*Participants' characteristics*: 437 female, 130 male<br>*Socioeconomic and demographic characteristics*: Not reported<br>*Comorbidities*: Self-reported medication usage for 36% heart disease or high blood pressure, 31% anxiety or depression 21% high cholesterol, 12.7% diabetes, 9% sleep apnea	The WMP was a 2-year long benefit, and a $20 monthly co-payment that allowed participants to meet with a registered dietitian, exercise physiologist, and certified personal trainer at various point throughout their time in the program. The majority of individuals in the program also spoke with a health behaviour counsellor via telephone every 6 to 8 weeks. The WMP was offered at approximately 60 approved exercise facilities in West Virginia, such as YMCAs, wellness centres, fitness centres, and physical therapy clinics.	Written responses to open ended response options within a questionnaire
First Author: Owen Smith<br>*Year:* 2014<br>*Category*: C<br>*Country*: UK	To present a synthesis of data from two qualitative studies in which both the development and the experience of living with morbid obesity in men and	Individuals who met the United Kingdom NICE criteria for a morbid obesity (BMI ≥ 40, or 35 kg/m^2 with comorbidity), and	*Role:* Participant<br>*Number of participants*: 31 (Study 1 n = 13; Study 2 n = 18)<br>*Participants characteristics*: 9 males, 3 age group 20–29, 11 age group 30–39, 7 age group 40–49, 9 age group 50–59, 1 60+ age group	The qualitative approach to both studies, to investigate individual experiences of developing and living with morbid obesity. The first study (Study 1) as part of a broader investigation into patients' experiences of implicit and explicit rationing. The core results the second study (Study 2) as part of an ongoing	Interviews

	women were explored in depth.	sought access to treatment for their condition	*Socioeconomic and demographic characteristics*: 15 non manual employment, 5 manual employment, 5 homeworker/carer, 1 retired, 4 unemployed<br>*Comorbidities*: Not reported	longitudinal study investigating how clinicians communicate with patients about the availability of treatment in the context of resource scarcity.	
First Author: Owen Smith<br>*Year:* 2016<br>*Category*: C<br>*Country*: UK	To focus on experiences of accessing treatment for morbid obesity in primary care.	Patients and providers at a weight management clinic at a general hospital in the South West of England	*Role:* Participant and providers<br>*Number of participants*: 22 patients<br>*Number of providers*: 11<br>*Participants' characteristics*: 7 male, 15 female, 9 age group 20-39, 12 age group 40-59, 1 age 60+<br>*Socioeconomic and demographic characteristics*: 21 white British, 4 professional, 8 other non-manual, 3 manual, 6 unemployed, 1 retired<br>*Comorbidities*: 19 joint pain/mobility issues, 11 depression/other depressive disorder, 10 breathlessness/respiratory difficulties, 9 diabetes, 8 hypertension, 4 sleep apnoea, 4 cardiac problems, 3 fertility issues<br>*Number of providers*: 11 clinicians<br>*Providers' characteristics*: Clinician informants included consultants and	Data collection was undertaken using in-depth interviews with patients and clinicians working in a specialist secondary care facility, and analysis took a constant comparative approach. Patients were followed from before their first consultation in secondary care up to 36 months after referral.	Interviews

			three allied medical professionals who worked within the weight management service.<br>*Socioeconomic and demographic characteristics*: Not Reported		

Categories: A= Qualitative and mixed-methods studies linked to eligible RCTs, including any qualitative data reported as part of papers reporting quantitative outcomes; B= Qualitative and mixed-methods studies linked to ineligible RCTs and identified non-randomised intervention studies including any reported qualitative data; C= UK-based qualitative studies not linked to any specific interventions that draw on the experiences and perceptions of adults with BMI ≥35 (and/or providers involved in their care). ¥=Studies included in review 2 (long-term randomised and non-randomised studies conducted in UK). BMI= Body Mass Index, calculated weight (kg) / height (m2)
