# Supplementary material for: Acceptability and feasibility of weight management programmes for adults with severe obesity: a qualitative systematic review
Source: BMJ Open. 2019 Sep 11;9(9):e029473. doi: 10.1136/bmjopen-2019-029473 (PMC6738728; doi:10.1136/bmjopen-2019-029473)
Supplement: Supplementary data [file bmjopen-2019-029473supp002.pdf]

REVIEW: Qualitative Studies

MEDLINE and EMBASE

Ovid multifile search: http://shibboleth.ovid.com/

Database: Embase <1980 to 2017 Week 31>, Epub Ahead of Print, In-Process & Other Non-Indexed Citations, Ovid MEDLINE(R) Daily and Ovid MEDLINE(R) <1946 to Present>
26th April 2017

Date of Search 26th April 2017

1 qualitative research/
2 exp interviews as topic/ use ppez
3 exp interview/ use emez
4 focus groups/ use ppez
5 grounded theory/
6 (qualitative or interview$ or focus group?).tw,kw.
7 (ethno$ or grounded or thematic or realist or interpretive or narrative or discourse analysis or discursive or mixed method$).tw,kw.
8 or/1-7
9 *obesity/
10 morbid obesity/ use emez
11 exp obesity, morbid/ use ppez
12 (obese or obesity).tw,kw
13 or/9-12
14 Weight Loss/ use ppez
15 weight reduction/ use emez
16 (weight adj1 (los$ or reduc$ or maint$ or control$ or manag$)).tw,kw.
17 (reduc$ adj2 (bmi or body mass index)).tw.
18 (reduc$ adj2 (waist adj3 (ratio$ or circumference))).tw.
19 (obesity adj1 manag$).tw,kw
20 anti obesity.tw,kw
21 or/14-20

22 8 and 13 and 21

23 (obes$ adj3 (morbid$ or severe$ or extreme$)).tw,kw.

24 8 and (10 or 11 or 23)

25 22 or 24

26 25 not (abstract or letter or note or comment).pt.

27 remove duplicates from 26

PsycINFO

Ovid: http://shibboleth.ovid.com/

Database: PsycINFO <1987 to April Week 3 2017>

Date of Search: 26th April 2017

1 qualitative research/

2 interviews/

3 grounded theory/

4 discourse analysis/

5 ethnography/

6 (qualitative or interview$ or focus group?).tw,kw.

7 (ethno$ or grounded or thematic or realist or interpretive or narrative or discourse analysis or discursive or mixed method$).tw,kw.

8 or/1-7

9 obesity/ or body weight/

10 (obese or obesity).tw,kw

11 9 or 10

12 Weight Loss/ or weight control/

13 (weight adj1 (los$ or reduc$ or maint$ or control$ or manag$)).tw,kw.

14 (reduc$ adj2 (bmi or body mass index)).tw.

15 (reduc$ adj2 (waist adj3 (ratio$ or circumference))).tw

16 anti obesity.tw,kw.

17 (obesity adj1 manag$).tw,kw

18 or/12-17

19 8 and 11 and 18

20 (obes$ adj3 (morbid$ or severe$ or extreme$)).tw,kw.

21 8 and 20

22 "obesity (attitudes toward)"/

23 19 or 21 or 22

CINAHL

http://search.ebscohost.com

!981- 25th April 2017

Date of Search: 25th April 2017

S1 (MH "Qualitative Studies+")

S2 (MH "Interviews") OR (MH "Semi-Structured Interview") OR (MH "Structured Interview")

S3 (MH "Focus Groups")

S4 (MH "Narratives")

S5 TX qualitative OR TX interview* OR TX focus group*

S6 TX (ethno* or grounded or thematic) OR TX (realist or interpretive or narrative) OR TX (discourse analysis or discursive or mixed method*)

S7 S1 OR S2 OR S3 OR S4 OR S5 OR S6

S8 (MH "Obesity") OR (MH "Obesity, Morbid")

S9 (MH "Body Weight")

S10 TX obese OR TX obesity

S11 S8 OR S9 OR S10

S12 (MH "Weight Control")

S13 (MH "Weight Loss")

S14 TX weight N1 los* OR TX weight N1 reduc* OR TX weight N1 maint* OR TX weight N1 control

S15 TX weight N1 manag* OR TX reduc* N2 bmi OR TX reduc* N2 body mass

S16 reduc* N2 waist ratio* OR TX reduc* N2 waist circumference TX

S17 S12 OR S13 OR S14 OR S15 OR S16

S18 (S7 AND S11 AND S17)

S19 (MH "Obesity, Morbid")

S20 TX obes* N3 morbid* OR TX obes* N3 severe OR TX obes* N3 extreme*

S21 S19 OR S20

S22 S7 AND S21

S23 (MH "Attitude to Obesity")

S24 S18 OR S22 OR S23

Science Citation Index and Social Science Citation Index

www.webofknowledge.com

1980 - 28th April 2017

Date of Search: 28th April 2017

1 TS=(qualitative or interview* or focus group)

2 TS=(ethno* or grounded or thematic or realist or interpretive or narrative or discourse analysis or discursive or mixed method*).

3 #1 OR #2

4 TS=(obesity or obese)

5 TS=(weight NEAR/1 los*) or TS=(weight NEAR/1 reduc*) or TS=(weight NEAR/1 maint*) or TS=(weight NEAR/1 control*) or TS=(weight NEAR/1 manag*).

6 TS=(reduc* NEAR/2 BMI) OR TS=(reduc* NEAR/2 body mass index)

7 TS=anti obesity

8 TS= (obesity NEAR/1 manag*)

9 #5 or #6 or #7 or #8

10 #3 AND #4 AND #9 *))) AND DOCUMENT TYPES: (Article)

CAB Abstracts

Ovid search: http://shibboleth.ovid.com/

Database: CAB Abstracts <1984 to 2017 Week 15>

Date of Search: 26th April 2017

1 qualitative analysis/

2 qualitative techniques/

3 (qualitative or interview$ or focus group?).tw.

4 (ethno$ or grounded or thematic or realist or interpretive or narrative or discourse analysis or discursive or mixed method$).tw.

5 or/1-4

6 obesity/
7 (obese or obesity).tw.
8 6 or 7
9 weight reduction/
10 (weight adj1 (los$ or reduc$ or maint$ or control$ or manag$)).tw.
11 (reduc$ adj2 (bmi or body mass index)).tw.
12 (reduc$ adj2 (waist adj3 (ratio$ or circumference))).tw.
13 (obesity adj1 manag$).tw
14 anti obesity.tw.
15 or/9-14
16 5 and 8 and 15
17 (obes$ adj3 (morbid$ or severe$ or extreme$)).tw.
18 5 and 17
19 16 or 18
