# Supplementary material for: Acceptability and feasibility of weight management programmes for adults with severe obesity: a qualitative systematic review
Source: BMJ Open. 2019 Sep 11;9(9):e029473. doi: 10.1136/bmjopen-2019-029473 (PMC6738728; doi:10.1136/bmjopen-2019-029473)
Supplement: Author's manuscript [file bmjopen-2019-029473.draft_revisions.pdf]

BMJ Open

BMJ Open is committed to open peer review. As part of this commitment we make the peer review history of every article we publish publicly available.

When an article is published we post the peer reviewers' comments and the authors' responses online. We also post the versions of the paper that were used during peer review. These are the versions that the peer review comments apply to.

The versions of the paper that follow are the versions that were submitted during the peer review process. They are not the versions of record or the final published versions. They should not be cited or distributed as the published version of this manuscript.

BMJ Open is an open access journal and the full, final, typeset and author-corrected version of record of the manuscript is available on our site with no access controls, subscription charges or pay-per-view fees (http://bmjopen.bmj.com).

If you have any questions on BMJ Open's open peer review process please email info.bmjopen@bmj.com

# The acceptability and feasibility of weight management programmes for adults with severe obesity: A qualitative evidence synthesis


| | |
|---|---|
| Journal: | *BMJ Open* |
| Manuscript ID | bmjopen-2019-029473 |
| Article Type: | Research |
| Date Submitted by the Author: | 28-Jan-2019 |


| | |
|---|---|
| Complete List of Authors: | Skea, Zoë; Universit of Aberdeen, Health Services Research Unit<br>Aceves-Martins, Magaly; University of Aberdeen, Health Services Research Unit<br>Robertson, Clare; University of Aberdeen, Health Services Research Unit<br>De Bruin, M; University of Aberdeen,<br>Avenell, Alison; University of Aberdeen, Health Services Research Unit |


| | |
|---|---|
| Keywords: | QUALITATIVE RESEARCH, PUBLIC HEALTH, NUTRITION & DIETETICS |


SCHOLARONE™
Manuscripts

# The acceptability and feasibility of weight management programmes for adults with severe obesity: A qualitative evidence synthesis

**Skea ZC, Aceves-Martins M, Robertson C, de Bruin M, Avenell A, and the REBALANCE team ***

**Author: Dr Zoë Christina Skea**

**Corresponding author: Dr Zoë Christina Skea, Health Services Research Unit, 3rd Floor, Health Sciences Building, University of Aberdeen, Aberdeen, AB25 2ZD, Tel: +44 (0) 1224 438153. email:z.skea@abdn.ac.uk. Fax: +44 (0) 1224 438165.**

**Co-authors: Dr Magaly Aceves-Martins, University of Aberdeen, Aberdeen, UK. Ms Clare Robertson, University of Aberdeen, Aberdeen, UK, Professor Marijn de Bruin, University of Aberdeen, Aberdeen, UK; Radboud Institute for Health Sciences, The Netherlands, Professor Alison Avenell, University of Aberdeen, Aberdeen, UK.**

**Key words: Qualitative research; Severe obesity; Weight management programmes.**

**Word count: 7165 words**

For peer review only

## Abstract

### Objectives

To undertake to a synthesis of published qualitative data to improve our understanding of the feasibility and acceptability of behavioural weight management programmes (WMPs) for adults with severe obesity and programme providers.

### Design

A systematic search and qualitative synthesis was conducted for published papers (from 1964- May 2017) that contained qualitative data from adults with BMI ≥ 35kg/m$^2$ (and/or the views of providers involved in their care) and considered issues relating to weight management.

### Participants

33 papers met our inclusion criteria from seven countries published 2007-2017. Findings were presented from a total of 644 participants and 153 programme providers (mostly from interviews or focus group sessions).

### Results



For peer review only - http://bmjopen.bmj.com/site/about/guidelines.xhtml

We found recurring themes around what programme participants described valuing and enjoying within WMPs. Participants described being attracted to programmes that were perceived to be novel or exciting in some key way, as well as programmes that had been endorsed by their health care provider (a view supported by programme providers themselves). The sense of belonging to a group who shared similar issues relating to weight and food, and who had similar physiques and personalities, was particularly important and seemed to foster a strong group identity and related accountability, which seemed to help with motivation and continuing engagement. Group based activities were apparently enjoyed by many and participants preferred WMPs with more intensive support from programme providers. However, some described struggling with physical activities (due to a range of physical co-morbidities) and not everyone enjoyed group interaction with others (sometimes due to various mental health co-morbidities).

**Conclusions**

Although group-based interventions were favoured, developers should bear in mind that people with very severe obesity might be especially vulnerable to both physical and mental co-morbidities which could inhibit engagement with certain intervention components (e.g. group based interaction; physical activities).

**Strengths and limitations of this study**

- To our knowledge, this is the first synthesis of key findings from qualitative studies exploring participants' and providers perspectives of Weight Management Programmes for adults with severe obesity (body mass index ≥35kg/m$^2$).
- Qualitative studies have a key role to play in understanding how factors facilitate or hinder the effectiveness of interventions, and how the process of interventions are perceived and implemented by users.
- Understanding the complex reasons why people with severe obesity chose to engage (or not) with lifestyle weight management programmes is important if health services are to be able to design effective intervention strategies to address and support weight management.
- Although the mean BMI reported across the papers ranged from 36.8 - 44.7kg/m$^2$, no quotes from participants in any of the included papers were linked to specific detail regarding BMI status.



For peer review only - http://bmjopen.bmj.com/site/about/guidelines.xhtml

## Introduction

There has been a continued increase in severe obesity (i.e. body mass index ≥35kg/m$^2$) in adults worldwide. As BMI increases, obesity-related comorbidities, social, psychological and economic consequences increase, with the potential need for greater support for help with weight loss. In the UK, having severe obesity, with or without comorbidities, may be a referral criterion for Tier 3 specialist weight management services in the obesity pathway, prior to Tier 4 services for bariatric surgery [1,2]. Effective weight loss services may reduce the need for bariatric surgery, and could also increase the effectiveness of subsequent bariatric surgery.

Qualitative studies have a key role to play in understanding how factors facilitate or hinder the effectiveness of interventions, and how the process of interventions are perceived and implemented by participants. This qualitative evidence synthesis was conducted as part of a larger systematic review funded by the UK's National Institute for Health Research Health Technology Assessment Programme [3] and aimed to improve our understanding of the feasibility and acceptability of non-surgical weight management programmes (WMPs) for adults with severe obesity and programme providers. Previous qualitative evidence syntheses have been undertaken [4,5] but these have not focussed on WMPs that are designed for or include people with severe obesity.

Our broad initial research questions included "What is it like to engage with (or be a provider of) weight loss interventions for adults with severe obesity?" and "What is it about interventions for adults with severe obesity that makes them helpful or unhelpful? Our review also considered issues around what might motivate people to decide to engage in such programmes.

This paper focuses on the main themes that emerged from the qualitative synthesis of included studies. These themes shed light on 1) motivating factors for engagement; 2) components of WMPs participants described valuing; and 3) general challenges for engagement.

## Methods

### *Searching and identification of relevant studies*

A systematic search was conducted in June 2016 and updated during April/May 2017 for published papers that contained qualitative data from adults with BMI ≥ 35kg/m$^2$ (and/or the views of providers involved in their care) and considered issues relating to weight management (See S1



For peer review only - http://bmjopen.bmj.com/site/about/guidelines.xhtml

Appendix for search strategies and S1 ENTREQ Checklist). Two researchers (ZCS and MAM) independently screened titles, abstracts and selected full text papers. Where consensus could not be reached regarding eligibility, a discussion at a research team meeting took place.

We included studies that fitted into the following broad categories:

A. Qualitative and mixed-methods studies linked to eligible RCTs (from our other review), including any qualitative data reported as part of papers reporting quantitative outcomes;
B. Qualitative and mixed-methods studies linked to ineligible RCTs and identified non-randomised intervention studies including any reported qualitative data;
C. Qualitative studies not linked to specific interventions that drew on the experiences and perceptions of adults with BMI ≥35kg/m$^2$ (and/or providers involved in their care) providing they reported data specifically relating to views/experiences of strategies for weight loss.

***Analysis and synthesis***

There are several approaches that can be used for synthesising the findings of qualitative studies.{6,7} Whilst being aware of the differing philosophical stances underlying various approaches to qualitative synthesis, we chose to adopt a pragmatic approach to our work in this area, which specifically aims to synthesise data that are relevant to informing policy and practice.{5} Our pragmatic approach corresponded most closely to a 'realist' perspective{7,8} as we were concerned with trying to find out not only 'what works' in terms of weight management for this group of adults and intervention providers, but also 'for whom, and under what circumstances'. At the same time, our approach was informed by and used aspects of review methods such as thematic synthesis{9,10} and analytical approaches developed from methods of inquiry such as grounded theory.{10}

In order to collate and synthesise the available primary research, two authors (ZS, MAM) each read and systematically extracted data from the included papers, shared notes and discussed study findings and interpretations during a series of group meetings. The papers were initially organised according to the categories described above but, as inductive analysis progressed, papers were grouped, compared, and contrasted according to emerging issues and themes. We used a data extraction form, which summarised the main themes, information regarding aims, methods, and any other important information relating to the context of the research within each study.

***Study quality***



For peer review only - http://bmjopen.bmj.com/site/about/guidelines.xhtml

The retrieved publications were appraised for methodological rigour and theoretical relevance independently by two reviewers using Toye's recently proposed criteria for quality in relation to meta-ethnography.{11} They suggest two core facets of quality for inclusion in syntheses of qualitative evidence, namely (1) Conceptual clarity: how clearly has the author articulated a concept that facilitates theoretical insight; (2) Interpretive rigour: what is the context of the interpretation; how inductive are the findings; has the interpretation been challenged? Two reviewers made notes regarding quality and results were compared and discussed.

***Patient and Public Involvement***

The REBALANCE Advisory Group included lay members who offered advice throughout various stages of this project including during initial discussions around the choice of appropriate research questions and areas of interest. Results were disseminated at a final project meeting in 2018 at which the Advisory Group were present.

**Findings**

***Description of studies***

The database search produced 4710 abstracts (See S1 Figure). Four additional papers were identified from included RCTs. In all, 33 papers met our inclusion criteria.{12-44}

The focus and key study characteristics of the 33 papers are outlined in S1 Table. The identified papers reported research conducted in seven countries (USA n=12; UK n=11; Norway n=3; Spain n=1; Canada n=2; Australia n=3; Mexico n=1), published between 2007 and 2017, and seven papers were linked to broader intervention studies: {15,16,18,25,37,38,39} Seven papers were classed as Category A; 24 Category B; and 2 Category C. As can be seen from S1Table, the studies had varying aims, but all offered insights into stakeholder's perceptions of weight loss strategies and programmes.

Although all the included papers provided some qualitative data for analysis, five of these provided qualitative data in the form of responses to open-ended survey questions within structured questionnaires.{17,27,32,41,44} Of those studies that used qualitative methods to collect their data, findings were presented from a total of 644 participants and 153 programme providers (mostly from interviews or focus group sessions).



For peer review only - http://bmjopen.bmj.com/site/about/guidelines.xhtml

Across the 33 papers, specific participant characteristics were inconsistently and poorly reported (if at all). Only 16 out of 33 papers provided any details. In terms of sex, information for 588 participants (out of 644 of those who specifically took part in qualitative evaluations) was provided – 372 female; 216 male. Age was reported across 15 papers, with the range being 19-88 years. Six of these papers provided mean age with the range being 40.2–67 years. BMI for those involved in qualitative evaluations was reported in nine papers. Of those that provided a mean, this ranged from 36.8-44.7kg/m$^2$. Only four papers gave details of participants' ethnicity; from 188 participants, 35 were reported as being from ethnic or racial minorities. Furthermore, 14 papers specifically stated that study participants had a range of additional physical and/or serious mental health problems (e.g. osteoarthritis, chronic pain, schizophrenia, post-traumatic stress disorder). It was also apparent across other included papers from quotes and/or author comments that many participants had a range of similar comorbidities.

Although no included papers provided qualitative data from those who had been invited to join a programme, but had declined to take part at recruitment stage, some papers reported including participants who had not fully engaged with programme activities (being described as 'low users'; 'quitters' or 'drop outs').{12,19,20,31}.

The WMPs varied in terms of the types and formats of support offered. Some programmes involved predominantly face to face interaction and activities with other participants and/or programme staff{19,22,24,26,27,28,29,30,35,40,42,} whereas others involved more remote forms of support (e.g. e-mail, telephone, text contact).{36,41} Other studies included and evaluated a mix of formats that also varied in intensity.{12,14,18,20,25,31,32,37,38,39,43,44}

Programmes incorporated a variety of tools and techniques designed to support behaviour change and to help people lose weight, e.g. tools such as diet diaries;{19,32} workbooks;{37,38,39} pedometers;{31,32,43} food logs; {12,42} conversation maps;{17} interactive monitoring devices;{41} social media group interaction; {14} daily text messages;{36} buddying;{32} and a range of behaviour change techniques and/or psychological support {15,16,21}such as goal setting;{27,28,31} motivational interviewing;{28} mindfulness;{309} self-determination theory based support;{19} regulatory focus theory;{36} self-regulation and cognitive behavioural techniques; {12,18,22,25,26,28,31,37,38,39} readiness to change and self-monitoring and feedback;{42} psychotherapeutic sessions;{29} emotional freedom therapy;{28}; neurolinguistic programming;{28} solution focussed therapy;{28} social learning theories;{35}



For peer review only - http://bmjopen.bmj.com/site/about/guidelines.xhtml

**Findings from the synthesis – participants**

***Motivating factors for engagement in WMPs***

Several papers provided insights into what had motivated prospective participants to take part in a specific WMP.{19,21,22,26,28,30,42}Important 'push' factors were sometimes internal to participants, for example expressing a desire to do something about their weight/poor physical fitness for themselves (e.g. as a result of growing health concerns and/or recent personal health scares) and also feelings of accountability to their families (e.g. stating that they wanted to be more engaged in activities with family members, as well as being there for family for as long as possible). Others recounted familial past experiences of health problems due to obesity or their own sudden and rapid weight gain due to mental health medication. For example:

***Recent personal health scares***

> *"I was told I was at risk of becoming diabetic." (No sample characteristics provided) {28}*

***Feelings of accountability to their families***

> *"I've had two kids in the last three years… that was part of the motivation… just getting fitter for my kids…I need to be aboot* [about] *for as long as possible" (Male).{26}*

***Familial past experiences of health problems due to obesity***

> *"My dad was a big guy and he developed diabetes, and he had to have surgeries and all kinds of stuff. I don't want to do that later in life." (intervention arm; no other sample characteristics provided). {42}*

***Sudden and rapid weight gain due to mental health medication***

> *"When I went on Zyprexa I gained a hundred pounds, very quickly. And that was really frustrating for me." (control arm; no other sample characteristics provided). {42}*

In addition to describing motivating factors that could be classed as internal, some participants described motivators that were apparently related to certain aspects of the programme intervention itself, for example, because it was perceived as being endorsed as credible by health professionals; perceived as being novel and exciting in some key way, and also because it provided an opportunity to engage with the intervention in a place that was valued.{21,22,26}



For peer review only - http://bmjopen.bmj.com/site/about/guidelines.xhtml

*"When I first went in there I thought this is great. I am going to diet at my doctor's surgery. Knowing that it was at my doctor's surgery gave me a big 'oof'." (no sample characteristics provided). {21}*

Although one paper highlighted that decisions to join a WMP were sometimes difficult and that some participants had expressed initial apprehension and reservations around taking part, {26} no included studies provided data about those who were invited to join but declined to take part at recruitment stage.

**Components of lifestyle programmes participants described liking or valuing**

We examined various aspects of WMPs that participants described valuing. In doing so, we were interested in the range of factors that might motivate those participants to join in the first place, continue to stay in the programme and also the factors that they described as having assisted them to change aspects of their behaviour or ways of thinking. All but two papers were set within the context of a WMP. The two included papers that were not linked to a specific intervention{33,34} also provided data regarding perceptions of weight loss strategies and engagement in diet and lifestyle programmes and were useful in this context. Unsurprisingly, there was variation in terms of what participants described as valuing within their WMP, demonstrating that a one size fits all approach is unlikely to be appropriate. We noted some key recurring themes in terms of what participants valued, and we grouped these around aspects that relate to a) the overall setting or context of the programme; b) the people (both other participants and health professionals/support staff) within the programme setting; c) the type of interaction/support offered; d) dietary elements; e) physical activities; and d) programme tools and techniques designed to support behaviour change. These are discussed below.

***a) The overall setting or context of the programme***

The overall setting of the programme was important for motivating people to decide to engage and also seemed important for motivating them to stay in and keep going with the various intervention activities. Some participants described their programmes as being exciting or novel in that they perceived them to be different to interventions they had tried previously (e.g. being focussed on physical activity rather than dieting;{19} being focussed on changing overall attitudes towards eating rather dieting *per se*;{30,38}) and an important consideration was the extent to which they could 'relate' to the nature of the programme (including how it was presented to them at recruitment) and how well it appeared to match with their own identities and values:{19,26,30,34}



For peer review only - http://bmjopen.bmj.com/site/about/guidelines.xhtml

*"…the main thing that drew us to it was because it's* [at a football club]*" (Male). {26}*

*"I always think somebody approaching you one-on-one is better. They can post all the weight loss you know pamphlets out there…I was hooked right away because somebody took the time to really explain it and take her time to do that." (Female). {30}*

Several participants also positively contrasted their overall perceptions of the WMPs with previous negative views towards other WMPs they had engaged with (e.g. WMPs which were perceived as being too 'feminine' or in some ways humiliating and embarrassing, or being perceived to be overly preoccupied with dieting;{19,20,24,27,28,34}

*"If you go to a slimming class you feel that you've made a fool of yourself or you get weighed and you've put on half a pound or a pound, and then you don't want to go back the next week so you don't go back." (Coaching group arm; no other sample characteristics provided). {20}*

*"Well, I think it's (WHEEL) appealed to me because I won't be dieting…I am obsessed with dieting me." (Female) {19}*

*"…spent many useless years at weight watchers with various leaders but never felt confident and in control or had the motivation I have now." (No sample characteristics provided). {27}*

***b) The importance of the people within the programme setting (for fostering a sense of accountability)***

A strong recurring theme was the value participants placed on perceiving themselves to be part of a like-minded group of individuals – individuals that faced similar issues, and who had similar physiques and personalities.{14,17,19,20,24,26,29} For example:

*"I do not feel so ashamed of my body here. We are all in the same situation, you see, which is really nice" (Female). {24}*



For peer review only - http://bmjopen.bmj.com/site/about/guidelines.xhtml

These perceptions seemed to foster a strong group identity and related 'accountability' in participants. Something that was apparently important for people in terms of motivating them to stick with the programmes and to not let their fellows down by dropping out or not sustaining behaviour changes:{12,14,19,20,26,30,31,32,42}

> *"So, you didn't want to disappoint yourself, but you didn't want to disappoint … your friends now either." (No sample characteristics provided). {30}*

Many participants also discussed the importance of their interactions with health care staff within the programmes.{12,19,20,22,24,27,28,29,30,32,35,38,40,44} They seemed to value the positive, friendly, and non-judgemental encouragement received and they also discussed feeling accountable to programme staff which helped with motivation. These aspects seemed to act as positive 'pulls' in terms of staying in the intervention and helping to sustain behaviour change:

> *"I think I just like talking to you [programme leader]. And I suppose I feel that if I don't do it [the programme] then I'm letting you down" (Female).{19}*

> *"She is my motivator… and she makes me keep a record of my diet" (Female). {24}*

***c) The type of interaction/support offered***

Although not universal, many described particularly valuing the social interactivity of group based programme activities and also fairly intensive support from/interaction with programme staff.{12,14,19,20,23,26,27,29,30,31,35,42,43} This appeared to function strongly as a motivator to maintain engagement with the WMPs by fostering feelings of accountability and by helping to ensure the achievement of pre-set goals:

> *"Oh God I haven't done what I should of done and I promised to do it and I know that isn't what's supposed to spur you on but it I think it does" (Regular support group; no other sample characteristics provided).{20}*

> *"[discussing feedback from programme staff]…great encouragement when the results are positive and a way to improve if the results are not so good." (No sample characteristics provided). {27}*



For peer review only - http://bmjopen.bmj.com/site/about/guidelines.xhtml

Participants discussed appreciating when the timing of support offered was flexible and could fit around their needs,{20,30,32} and several wanted more support than was offered within the programmes (e.g. more frequent contact and for a longer duration than the programme currently allowed).{20,31,41,44} Many also expressed concern about support ending post-intervention{19,20,24,30,36,42} with the suggestion that diminishing intensity of programme activities and/or programme cessation could cause problems for maintaining behaviour change patterns if group interaction and support were key parts of it:

*"I cannot do it without her support, it just wouldn't work" (Female). {24}*

Some WMPs involved predominantly face to face interaction and activities with other participants and/or programme staff. {19,22,24,26,27,28,29,30,35,40,42} In contrast, others involved more remote forms of support (e.g. e-mail, telephone, text contact). {36,41} Some studies included and evaluated a mix of formats that also varied in intensity. {12,14,18,20,25,31,32,37,38,39,43,44} Many participants discussed valuing the social interactivity of the in person group based activities{14,19,20,26,30,31,42} and, where it was discussed and compared, participants tended to value and desire human contact over more remote forms of support. {31,41} This preference seemed to be linked to incentivising people to stay committed to the various programmes and was also apparently important in terms of making participants feel accountable to a likeminded group of individuals.

***d) Dietary elements***

Some WMPs provided detailed dietary advice regarding food choices, whilst others specifically described interventions as 'non-dietary' (nevertheless, incorporating behavioural change techniques to support attitudinal changes towards food and eating patterns). We examined data that were available from participants and/or programme staff relating to the perceived usefulness or otherwise of these dietary aspects. Although views were sometimes mixed, participants tended to describe valuing the flexibility and variety of diet format. {19,30,31,35} This seemed important in terms of helping them to 'normalise' and stabilise their eating habits, particularly as many had attempted diets over a period of many years (without success) leading them to develop negative and unhealthy relationships towards food.{19,30,31,35}

*"The other programs told you not to eat this or that and you were afraid to go back if you hadn't lost weight and …they tell you that you can eat everything but you yourself have to*



For peer review only - http://bmjopen.bmj.com/site/about/guidelines.xhtml

*control the amount…You make up the diet every day and that's very motivating" (Female).{35}*

***e) Physical activities***

All of the WMPs incorporated some attention to increasing physical activity. Whilst clearly some participants described struggling to engage in exercise for a variety of reasons, many participants described the positive psychological and physical benefits they experienced from exercising.{14,19,24,28,42}

*"When I first started I could hardly walk…now I can walk 300-400 yards…if this project has done nothing else it has helped me to walk (no sample characteristics provided." (No sample characteristics provided).* {28}

When it was offered as part of the WMP, participants also discussed valuing the flexibility of being able to choose from a variety of exercise formats and approaches. {19,31}

***f) Programme tools and behaviour change techniques designed to support behaviour change***

We examined data that were available from participants and/or programme staff relating to the perceived usefulness or otherwise of tools and techniques designed to support behaviour change and to help people lose weight. Although not universally popular, {12,19,31,41,42} participants described the incorporation of tools, such as food logs, goal setting, regular text messages, tele-monitoring devices and conversation maps as being motivating, and also helpful for the purposes of education and learning, describing how they helped to facilitate self-awareness of and reflection on eating and other behaviour patterns. {12,17,31,32,36,41,42,43,44}

*"I found it to be very enlightening. It made me start to look at foods differently It has given me a more conscious outlook on how to control my diabetes and the importance of exercise." (No sample characteristics provided). {17}*

*"What really helped me was having somebody go over the food log every day. That was the big thing." (No sample characteristics provided). {12}*



For peer review only - http://bmjopen.bmj.com/site/about/guidelines.xhtml

Participants discussed the positive psychological changes they experienced with regards to their relationship to food/body image, which seemed to relate to the BCTs employed within some of the WMPs (e.g. mindfulness and self-determination theory based support).{12,19,22,30}

## General challenges for engagement in WMPs

Despite the numerous positive comments from within the data with regard to programme engagement, participation was not straightforward for everyone who took part. General challenges resulting in decreased engagement (or success) related to a number of factors. Sometimes, these involved the timing of clinic appointments;{32} cost of travel to appointments;{28,43} general low self-efficacy;{21} family members not being on board, such that behavioural changes were difficult to sustain;{29,42} whereas others described factors which could be described as life getting in the way (e.g. holidays, social events, bad weather as disincentive to exercise). {42}

It was apparent that participants experienced a range of comorbidities, including some serious mental health issues. {13,14,31,32,33,34,41,42,43} Sometimes these specific illnesses presented challenges for motivation and continuing engagement, for example, feeling too ill to focus on weight/feeling too ill to care or to be motivated:{28,31,34,35,42}

> *"Because of the ME* [myalgic encephalopathy] *I'm sleeping fifteen or more hours a day, and so exercise is out of the question because I can't even walk to the end of the road." (Female).{33}*

## Critical reflections on specific components of WMPs

### *The type of interaction/support offered*

The recurring theme of valuing the social interactivity of group-based programme activities was not universally valued by all, with some describing a reluctance to discuss issues within a group setting.{14,22,23,35,40,43} This was perhaps particularly pertinent in studies where participants had additional mental health issues:

> *"I know the importance of the program is to be together, but at the beginning you don't know these people, some of us have problems interacting with people we don't know." (No sample characteristics provided. {14}*

> *"It's just I don't like to be around people." (No sample characteristics provided). {43}*



For peer review only - http://bmjopen.bmj.com/site/about/guidelines.xhtml

*"I prefer to talk in private as I suffer from panic attacks." (No sample characteristics provided). {40}*

One study{38} included data that suggested some participants felt guilty using up what they perceived to be too much of their health care provider's time (in an intervention involving regular GP visits):

*"I must admit I felt frequently embarrassed that I was taking up a lot of my GP's time." (No sample characteristics provided). {38}*

**Dietary elements and physical activities**

Although the majority of participants tended to describe valuing the flexibility and variety of the diet formats offered within programmes,{19,31,35,44} views were sometimes mixed with regard to diets, with a few wanting more prescriptive and structured eating plans than were offered:

*"I think [having a set meal plan to follow] would have been to a certain extent easier at the beginning, but I don't think it would of actually adjusted my attitudes and thinking which it [POWeR+] has done (Male; 64 years; face-to-face support; high user)." (No sample characteristics provided).{31}*

The above quote illustrates that participants often discussed appreciating when programmes apparently emphasised changing attitudes towards food and eating over promoting a specific diet *per se*. However, sometimes participants did feel that their programme (or their primary care providers) tended to over emphasise diet rather than, for example, addressing issues around exercise, sleep or addiction problems.{34,42}

*"…there was no support counselling-wise as to why I have the issues I have with food…" (Male).{34}*

Whilst many participants described the positive psychological and physical benefits they experienced from exercising, {14,19,42} others described struggling to engage in exercise. Some described disliking the perceived high intensity of the exercises (e.g. feeling uncomfortable with



For peer review only - http://bmjopen.bmj.com/site/about/guidelines.xhtml

sweating, {19,23,24} whilst others discussed how their various physical or mental health comorbidities could prohibit them from full engagement in activities.{13,19,23,24,31,32,33,34,42}

> *"Exercise is the best [to lose weight] and I get all this physical therapy exercise and all of that just increases my pain, which reduces my desire to have any exercise." (No sample characteristics provided).{13}*

> *"I think for me, with my disability it was difficult to engage with some of the activities recommended." (No sample characteristics provided).{32}*

**Programme tools and BCTs designed to support behaviour change**

Participants suggested that many of the WMP's tools and techniques were helpful for them in terms of reflecting on their habits and behaviours and for helping them to positively change their attitudes. However, some participants described these tools as being somewhat intrusive and sometimes inflexible in nature. For example, some participants described disliking food logs and found food diaries/goal setting/daily self-weighing and the monitoring of exercise as excessive and too confrontational.{19,31,41,42} Others felt that programme staff did not appropriately monitor and feedback on progress:{12}

> *"I mean no one ever looked at it [food diary]. No one ever asked for it. I just did all the work, like, for nothing because no one ever asked me for it." (No sample characteristics provided). {12}*

Others expressed frustration with the perceived inflexibility of tools designed to record behaviour and activities and to support behaviour change. For example, not being able to record life events and/or comorbidities that might help to explain lack of achievement regarding weight loss:{31,36}

> *"I thought that might be useful [to] have something [to] explain why things are going as they are going." (Female; 59 years, remote support; high user). {31}*

> *"I would want to tailor the messages [daily text messages] to the things that I was most struggling with." (No sample characteristics provided). {36}*



For peer review only - http://bmjopen.bmj.com/site/about/guidelines.xhtml

With regard to psychological support, two papers highlighted that some people wanted more counselling for non-direct weight issues, such as mental health, recognising that these additional problems had implications for weight management. {34,41} In contrast, although many participants discussed the various positive psychological changes they experienced which seemed to relate to the BCTs/counselling employed within some of the WMPs, others found personal development classes challenging and confrontational and questioned their appropriateness:{22}

> *"I cannot benefit from it [the personal development classes]. I will never open up in that room and talk among others." (Male). {22}*

**Findings from the synthesis – provider participants**

Ten of the included papers provided qualitative data from a range of WMP providers. {15,16,18,21,22,25,31,36,37,38} Seven of these papers were linked to one of three of the same interventions. Programme providers who provided qualitative data were described as primary care providers; {18,25} nurses; {31} GPs and consumer representatives;{38} GPs; {37,39} mental health care workers, dietitians, and nurses; {15,16} GPs, weight management advisors, practice nurses, {21} and key personnel working at a residential weight loss centre. {22}

***General impressions of being involved in WMPs***

With the exception of one study, in which some GPs (but not all) were reportedly less enthusiastic, {21} views about being involved in a WMP were generally very positive, with health professionals acknowledging that engagement was potentially very useful for them in terms of facilitating a conversation around weight loss with participants, and recognising that this can often be challenging in their everyday practices. {31,37,38,39}

However, the authors of one study{15} noted that discussions about weight tend to be embedded within the context of conversations about other health issues (rather than being discrete or stand-alone) and argued that this could act as a potential barrier with regards to the implementation of WMPs within primary care:

> *"I don't have patients that come to see me just for obesity or…just one thing…yes they're one of my diabetic patients but … we're talking about their cholesterol today or their blood pressure and their weight another day." (Nurse, no other sample characteristics provided).* {15}



For peer review only

***Motivating factors for participants'/provider engagement in WMPs***

One paper included some insights from the perspectives of programme providers about what apparently motivated prospective participants to take part in a WMP. {18} Health care providers involved in the delivery of the programmes described how they regarded participants' perceptions of their professional 'buy in' to the intervention study (i.e. endorsement) as important and influential regarding their decisions to take part. {18} One study (linked to two papers){18,25} also reported unusual success at enrolling men which programme providers attributed to their endorsing it as a 'medical' programme:

> *"I think that [our affiliation with a research institution] helped make it into a legitimate type of program that* [our patients] *would have confidence in, not just one of these wild watermelon diets or things like that." (Primary Care Provider, no other sample characteristics provided).{18}*

In terms of disincentives towards retention in such WMPs, some providers reported that some participants could have unrealistic expectations about weight loss, not fully understanding programme goals and commitment and wanting a "quick fix":

> *"What they wanted was a quick fix…They want to lose pounds very quickly. And it doesn't happen…*"(*GP, no other sample characteristics provided)* {21}

Only one study {21} provided data around apparent barriers and facilitators to health professionals' own engagement with a specific WMP. They described how clinicians' pre-conceived beliefs and attitudes towards integrating WMPs into primary care settings were important and they noted that engaged practices (as opposed to less engaged practices) were characterised by active GP participation and 'buy in.'

***The importance of the people within the programme setting (for fostering a sense of accountability)***

In keeping with some key findings from participants across the included papers, programme providers reflected on the importance of WMPs for creating a sense of accountability both for themselves as professionals in terms of increasing their responsiveness and sensitivity to their participants' weight management plan and needs and also of their continued engagement, motivation and success: {18,37}



For peer review only - http://bmjopen.bmj.com/site/about/guidelines.xhtml

> *"...I think it just made me be more sensitive…I've been kinda tryin' to dial it* [being tough on the patients*] down a little bit" (Primary Care Provider, no other sample characteristics provided) {18}*

Programme providers also recognised and reflected on what they regarded to be the importance of establishing and maintaining good relationships and of giving positive reinforcement and encouragement and being supportive of their weight loss efforts.{15,18,25,31}

***The types of interaction/support offered***

Several health care providers recognised that the intensity of interactions between programme staff and participants was important for motivating the latter to stay engaged and to sustain behaviour changes. {18,25} However, several provider participants raised concerns about the reality of this for their everyday clinical practice when time constraints were a real issue. {15,16,38} Other health care providers raised concerns around a lack of interdisciplinary working within clinic settings, which could inhibit their abilities to support weight loss, as well as lack of clarity with regard to professional role remits within teams:

> *"I work with our RN all the time so on a daily basis we talk about things going back and forth but the others* [referring to dietitian and mental health workers] *I don't really see to be honest." (Nurse, no other sample characteristics provided). {16}*

Although providers in the above study{16} raised broad issues in their interviews relating to these barriers, they reflected positively on the study WMP in terms of facilitating interdisciplinary collaboration.

**Views about mode of support**

In terms of views about mode of support, health providers in one primary care study{18} argued that telephone-delivered weight counselling was the most convenient for participants. In contrast, providers in another study (one that involved a residential WMP) {22} argued that face-to-face group interaction was essential and particularly useful for participants with severe obesity who often experience social isolation. In another primary care study, {31} views regarding mode of delivery of support were more mixed. Whilst recognising the practicalities of remote forms of support, programme providers (in this case nurses) argued that face-to-face interactions worked



For peer review only - http://bmjopen.bmj.com/site/about/guidelines.xhtml

best in terms of helping them connect more effectively and facilitated participant engagement and motivation. Some even stated that they did not regard remote support as support at all.

**Views about levels of provider engagement**

Health care providers in one study{18} stated that they played a fairly peripheral role in aspects of programme delivery and that sometimes this made it difficult for them to fully engage with their patient and to assess their progress. They suggested that individualised feedback from other professionals involved in programme delivery (e.g. in this case weight loss health coaches) would have been helpful. However, the study also reported that the majority of health care providers valued the fact that they played a limited role in the WMP, with time constraints and specific skill sets being raised as issues. Another study {31} raised related issues around level of provider engagement with aspects of the WMP. In this case, nurses discussed the perceived disadvantage of not being able to view the information provided to participants on the study website. Some felt that viewing this information would have allowed them to understand more fully, what participants were referring to in consultations. In one study,{38} GPs commented on and seemed to value the relatively 'loose' nature of the intervention design (in this case a weight management toolkit) as they considered it offered scope to enable them to tailor it to the individual and their community. Similarly, nurses in another study {31} expressed frustration around the lack of flexibility of their intervention, both in terms of how they were supposed to behave (i.e. by not being directive) and in terms of the scope within the website to document individual issues. This was also a concern raised by the participants themselves. Although, providers in these two studies {31,38} apparently appreciated interventions that were more flexible in nature (and therefore could be tailored more appropriately to individual care). Personnel in a residential WMP{22} specifically designed for people with severe obesity seemed to value having a very strict programme structure (in this case participants had to attend morning meetings, group activities, and eat six meals a day at fixed times). The general feeling amongst staff was that instilling this strictness on participants would facilitate behaviours that they would then seek to maintain at home.

**Views about intervention content**

Whilst some, (but not all), participants in one study{22} found personal development classes challenging and confrontational, providers in the same study consistently argued that personal development (i.e. focussing on internal factors such as self-knowledge and self-acceptance) was essential and crucially important for maintaining lifestyle changes longer term:



For peer review only - http://bmjopen.bmj.com/site/about/guidelines.xhtml

*"It is important that they become aware of what in their life makes a difference in being obese or not." (Personnel, no other sample characteristics provided).{22}*

**Discussion**

*Principal findings*

This review synthesised findings from qualitative data relating to the views of adults with BMI ≥35kg/m$^2$ (and/or their health care providers) about engaging with WMPs. In summary, although there was variation expressed in views about the acceptability of various programme components (indicating the inappropriateness of a 'one size fits all' approach), there were, nevertheless, recurring themes around what both participant and programme providers described valuing and enjoying. Some of these key findings resonate with previous qualitative research with people with less severe obesity. {4,45}.

Participants in our review described being attracted to WMPs that were perceived to be novel or exciting in some key way (e.g. being different to programmes that they had tried previously), as well as perceived to have been endorsed by their health care providers (a view supported by programme providers themselves). The sense of belonging to a group of people who shared similar issues relating to weight and food, and who had similar physiques and personalities, was described as being particularly important to many participants and seemed to foster a strong group identity and related 'accountability', which seemed to help with motivation and continuing engagement.

Good relationships with programme providers were described as being highly valued, with ongoing encouragement and monitoring apparently important for facilitating motivation and behaviour change (a view also endorsed by the programme providers themselves). Group based programme activities were apparently enjoyed by many participants along with fairly intensive support from programme providers. This observation is supported in previous qualitative research with people with less severe obesity.{4,45}. However, in our review, although described by both participants and programme providers as being important for supporting engagement and positive behaviour changes, concerns were raised about the availability of continuing support post intervention, and similarly by providers who questioned the practicalities and logistics of integrating such intense support into their everyday clinical practices once the studies were completed.



For peer review only - http://bmjopen.bmj.com/site/about/guidelines.xhtml

Overall, both participants and programme providers valued having choice and flexibility. For example, participants welcomed flexibility around diet choices, flexibility around when face-to-face counselling sessions, and also welcomed personalised interventions. Similarly, some programme providers found the perceived lack of flexibility with various intervention components frustrating and prohibitive in terms of supporting individualised care.

Those participants who described engaging in group discussions/therapy sessions (with other participants and/or providers) and those who discussed engaging in exercises were mainly positive about their perceived benefits. For example, where it was discussed, participants very much valued the psychological input integrated into many interventions. This is a view supported in a study of user experiences of both Tier 2 and Tier 3 weight management services in England.{45} However, it is worth noting that our review also highlighted that some participants did describe struggling with these aspects, with some describing them as particularly challenging. Some participants described difficulties with the various physical activities (because of a range of physical comorbidities) and not everyone enjoyed group interaction and discussions with others (sometimes apparently because they suffered from various mental health comorbidities).

*Strengths and limitations*

To our knowledge, this is the first synthesis of key findings from qualitative studies exploring participants' perspectives of WMPs for adults with severe obesity. Our synthesis has highlighted a range of important factors that have the potential to facilitate engagement with WMPs for this group.

We were interested in ascertaining the views of participants with severe obesity (people with BMI $\geq$35kg/m$^2$). Therefore, our inclusion criteria were that papers needed to state that participants in their respective studies (i.e. either in their qualitative evaluations or the intervention studies to which their qualitative evaluations were linked) had a mean BMI $\geq$35kg/m$^2$. Of those papers that only considered programme providers' views, these had to be linked to intervention studies where we could establish that included participants had a mean BMI $\geq$35kg/m$^2$. Only two papers stated that their respective WMPs were designed *specifically* for people with BMI $\geq$35kg/m$^2$. {22,40} Thus, across the papers, some people with BMI <35kg/m$^2$ would have been included. Quotes from participants were not linked to specific detail regarding BMI status, and so we cannot be certain that findings reflect exclusively the views of those with severe obesity.



For peer review only - http://bmjopen.bmj.com/site/about/guidelines.xhtml

Only nine papers linked participant quotes to sex; {19,22,24,26,30,31,33,34,35} only one to age status;{31} and none to socioeconomic/demographic characteristics, making it hard for us to consider whether any issues raised were particularly sensitive or pertinent to these aspects.

We know from a recent review of Tier 3 weight management interventions for adults with severe obesity that drop-out rates are very high (43-63%). {46} Only four of our included papers stated that some of the participants in their qualitative evaluations had been ‘low users’, ‘quitters’ or ‘drop-outs’{12,19,20,31} and only one of these papers linked quotation data directly to intervention usage status. {31} Although our findings highlighted a range of views with regard to the usefulness or otherwise of various intervention components, it is worth noting that participant sample characteristics within the included papers are skewed towards those who had chosen to engage and who had completed the various intervention activities.

Applying quality criteria to qualitative research remains a contentious issue and there is no consensus regarding whether and how this should be done (47,48). Whilst authors of some qualitative evidence syntheses have chosen to exclude what they deem to be poor quality papers, we made the decision not to exclude any of the identified papers. We included 33 papers that each reported some qualitative data that met our inclusion criteria and addressed our key research questions. Although all included qualitative data, in terms of ‘quality,’ some were deemed richer than others in terms of data and insights - some ranged from being exclusively qualitative studies providing rich data in our areas of interest, through to studies that were actually primarily quantitative with responses to open-ended survey questions. The five studies providing qualitative data in the form of responses to open-ended survey questions within structured questionnaires{17,27,32,41,44} were deemed less useful in terms of presenting only very limited qualitative data and insights. Despite this variation in the overall level of quality, we felt it was more important to retain any relevant findings rather than disregard based on study quality. In doing so, we would argue that all 33 papers contributed useful elements to the collective whole and enabled us to develop our understanding of the issues of importance to people with BMI $\geq$35kg/m$^2$. We cannot exclude the possibility that unpublished service evaluations from within the NHS, that we failed to locate, might have been sources of rich data.

*Practice Implications*

Within our review, it was clear that ongoing encouragement and monitoring by programme providers was viewed as important for facilitating motivation and behaviour change. However,



For peer review only - http://bmjopen.bmj.com/site/about/guidelines.xhtml

intervention developers should bear in mind that waning intensity of programme activities and/or programme cessation could cause problems for maintaining behaviour change patterns if group interaction and support was an initial integral component.

People with very severe obesity might be especially vulnerable to both physical and mental comorbidities, which could inhibit engagement with certain intervention components (e.g. group based interaction; physical activities). For intervention developers, this is worthy of note. This could inhibit their engagement with much fitter peers with fewer weight-related issues, or restrict their ability to undertake certain intervention components – an observation that is perhaps less apparent in research with people with less severe obesity [4].

*Implications for research*

No papers included in our review provided qualitative data from those who had been invited to join a WMP but who had declined to take part, and only four papers reported including participants who had not fully engaged with all programme activities to varying degrees. Therefore, in terms of pointers for effective interventions, it is worth acknowledging that key findings will be skewed towards those who had chosen to engage and who had completed the various intervention activities. In terms of implications for research, it is clear that the qualitative research literature focusing specifically on lifestyle WMPs for people with very high BMIs is limited, particularly for people who are low-users or do not wish to engage with such services.

*Conclusions*

WMPs that are perceived to be novel or exciting and WMPs that are perceived to be endorsed by health care providers tend to be valued by participants. The sense of belonging to a group of people who share similar issues and characteristics seems particularly important, helping to foster a strong group identity and related 'accountability', which aids motivation and continuing engagement. In person group based programme activities tend to be valued (over more remote forms of support), along with fairly intensive support from programme providers. However, intervention developers should bear in mind that people with very severe obesity might be especially vulnerable to both physical and mental co-morbidities that could inhibit engagement with certain intervention components.

Supporting Information

S1 Appendix: Search strategies



For peer review only - http://bmjopen.bmj.com/site/about/guidelines.xhtml

S1 ENTREQ Checklist

S1 Table: Characteristics of included studies

S1 Figure

**Acknowledgements**

*REBALANCE Project management team Elisabet Jacobsen[1], Dwayne Boyers[2], David Cooper[1], Lise Retat[3], Paul Aveyard[4], Fiona Stewart[1], Graeme MacLennan[1], Laura Webber[3], Emily Corbould[3], Benshuai Xu[3], Abbygail Jaccard[3], Bonnie Boyle[1], Eilidh Duncan[1], Michal Shimonovich[1], Cynthia Fraser[1], Lara Kemp[1].

REBALANCE Advisory Group for all their advice and support during this project: Margaret Watson, Lorna Van Lierop, Richard Clarke, Jennifer Logue, Laura Stewart, Richard Welbourn, Jamie Blackshaw, Su Sethi.

[1]Health Economics Research Unit, University of Aberdeen, Aberdeen, UK

[2]Health Services Research Unit, University of Aberdeen, Aberdeen, UK

[3]UK Health Forum, Fleetbank House, Salisbury Square, London, UK

[4]Nuffield Department of Primary Care Health Sciences, Oxford University, Oxford, UK

**Contributors**

AA and ZCS conceived the study idea for the qualitative synthesis. ZCS and MAM screened all titles and abstracts. ZCS and MAM conducted the data analysis and ZCS wrote the initial and subsequent manuscript drafts. All the authors contributed critically to discussions about interpretation of data and revisions of manuscript drafts. All the authors approved the final version.

**Funding**

The project was funded by the NIHR Health Technology Assessment Programme (Project number: 15/09/04). See the HTA Programme website for further project information.

The views and opinions expressed therein are those of the authors and do not necessarily reflect those of the Department of Health, or the funders that provide institutional support for the authors of this report.

**Data sharing statement**

This is a review of published studies which are available to access through the relevant journals.

**Competing interests statement**

There are no competing interests for any author.



For peer review only - http://bmjopen.bmj.com/site/about/guidelines.xhtml

1. National Institute for Health and Care Excellence. Obesity: identification, assessment and management of overweight and obesity in children, young people and adults. (Partial update of CG43). 2014. http://www.nice.org.uk/guidance/cg189
2. Royal College of Surgeons. Commissioning guide: weight assessment and management clinics (tier 3), 2014. https://www.rcseng.ac.uk/healthcare-bodies/docs/weight-assessment-and-management-tier-3-services.
3. Avenell A, Robertson C, Skea ZC, Jacobsen E, Boyers D, Cooper D et al. Bariatric surgery, lifestyle interventions and orlistat for severe obesity: The REBALANCE mixed-methods systematic review and economic evaluation. Health Technology Assessment, vol 22,no.68.
4. Sutcliffe K, Richardson M, Rees R, Burchett H, Melendez-Torres GJ, Stansfield C, Thomas J (2016) What are the critical features of successful Tier 2 weight management programmes for adults? A systematic review to identify the programme characteristics, and combinations of characteristics, that are associated with successful weight loss. London: EPPI-Centre, Social Science Research Unit, UCL Institute of Education, University College London.
5. Robertson C, Archibald D, Avenell A, Douglas F, Hoddinott P, van Teijlingen E, et al. Systematic reviews of and integrated report on the quantitative, qualitative and economic evidence base for the management of obesity in men. Health Technol Assess 2014;13:35.
6. Barnett-Page E, Thomas J. Methods for the synthesis of qualitative research: A critical review. *BMC Med Res Method* 2009;**9:**59**.**
7. Spencer L, Ritchie J, Lewis J, Dillon L. Quality in qualitative evaluation: a framework for assessing research evidence. London: Cabinet Office; 2003
8. Pawson R. Evidence-based policy: a realist perspective. London: Sage; 2006
9. Thomas J, Harden A. Methods for the thematic synthesis of qualitative research in systematic reviews. *BMC Med Res Method* 2008;**8:**45.
10. Harden A, Brunton G, Fletcher A, Oakley A. Teenage pregnancy and social disadvantage: systematic review integrating controlled trials and qualitative studies. *BMJ* 2009;**339:**b4254.
11. Toye F, Seers K, Allcock N, Briggs M, Carr E, Andrews J, et al. 'Trying to pin down jelly' - exploring intuitive processes in quality assessment for meta-ethnography. *BMC Med Res Method* 2013;**13:**46.
12. Abildso C, Zizzi S, Gilleland D, Thomas J, Bonner D. A mixed methods evaluation of a 12-week insurance-sponsored weight management program incorporating cognitive-behavioral counseling. *J Mix Methods Res* 2010;**4:**278-94.
13. Amy Janke E, Kozak AT. "The more pain I have, the more I want to eat": obesity in the context of chronic pain. *Obesity* 2012;**20:**2027-34.
14. Aschbrenner KA, Naslund JA, Bartels SJ. A Mixed Methods Study of Peer-to-Peer Support in a Group-Based Lifestyle Intervention for Adults With Serious Mental Illness. *Psychiatr Rehabil J* 2016;**39:**328-34.
15. Asselin J, Osunlana AM, Ogunleye AA, Sharma AM, Campbell-Scherer D. Missing an opportunity: the embedded nature of weight management in primary care. *Clin Obes* 2015;**5:**325-32.
16. Asselin J, Osunlana AM, Ogunleye AA, Sharma AM, Campbell-Scherer D. Challenges in interdisciplinary weight management in primary care: lessons learned from the 5As Team study. *Clin Obes* 2016;**6:**124-32.
17. Barham K, West S, Trief P, Morrow C, Wade M, Weinstock RS. Diabetes prevention and control in the workplace: a pilot project for county employees. *JPHMP* 2011;**17:**233-41.
18. Bennett WL, Gudzune KA, Appel LJ, Clark JM. Insights from the POWER practice-based weight loss trial: a focus group study on the PCP's role in weight management. *J Gen Intern Med* 2014;**29:**50-8.



For peer review only - http://bmjopen.bmj.com/site/about/guidelines.xhtml

19. Borkoles E, Carroll S, Clough P, Polman RCJ. Effect of a non-dieting lifestyle randomised control trial on psychological well-being and weight management in morbidly obese pre-menopausal women. *Maturitas* 2016;**83:**51-8.
20. Bradbury K, Dennison L, Little P, Yardley L. Using mixed methods to develop and evaluate an online weight management intervention. *Br J Health Psychol* 2015;**20:**45-55.
21. Counterweight Project T, McQuigg M, Brown JE, Broom JI, Laws RA, Reckless JP, et al. Engaging patients, clinicians and health funders in weight management: the Counterweight Programme. *Fam Pract* 2008;**25 Suppl 1:**i79-86.
22. Dahl U, Rise MB, Kulseng B, Steinsbekk A. Personnel and participant experiences of a residential weight-loss program. A qualitative study. *PLoS ONE* 2014;**9:**e100226.
23. Danielsen KK, Sundgot-Borgen J, Rugseth G. Severe Obesity and the Ambivalence of Attending Physical Activity: Exploring Lived Experiences. *Qual Health Res* 2016;**26:**685-96.
24. Groven KS, Engelsrud G. Dilemmas in the process of weight reduction: Exploring how women experience training as a means of losing weight. *Int J Qual Stud Health Well-being* 2010;**5**.
25. Gudzune KA, Clark JM, Appel LJ, Bennett WL. Primary care providers' communication with patients during weight counseling: a focus group study. *Patient Educ Couns* 2012;**89:**152-7.
26. Hunt K, Gray CM, Maclean A, Smillie S, Bunn C, Wyke S. Do weight management programmes delivered at professional football clubs attract and engage high risk men? A mixed-methods study. *BMC Public Health* 2014;**14:**50.
27. Jackson C, Coe A, Cheater FM, Wroe S. Specialist health visitor-led weight management intervention in primary care: exploratory evaluation. *J Adv Nurs* 2007;**58:**23-34.
28. Jennings A, Hughes CA, Kumaravel B, Bachmann MO, Steel N, Capehorn M, et al. Evaluation of a multidisciplinary Tier 3 weight management service for adults with morbid obesity, or obesity and comorbidities, based in primary care. *Clin Obes* 2014;**4:**254-66.
29. Jimenez-Lopez JL, Maldonado-Guzman ME, Flores-Perez Pasten L, Deciga-Garcia E. [Reasons for losing weight: Why have programed support?]. *Rev Med Inst Mex Seguro Soc* 2012;**50:**407-12.
30. Kidd LI, Graor CH, Murrock CJ. A mindful eating group intervention for obese women: a mixed methods feasibility study. *Arch Psychiatric Nurs* 2013;**27:**211-8.
31. Little P, Stuart B, Richard Hobbs FD, Kelly J, Smith ER, Bradbury KJ, et al. Randomised controlled trial and economic analysis of an internet-based weight management programme: POWeR+ (Positive Online Weight Reduction). *Health Technol Assess* 2017;**21:**1-61.
32. McRobbie H, Hajek P, Peerbux S, Kahan BC, Eldridge S, Trepel D, et al. Tackling obesity in areas of high social deprivation: clinical effectiveness and cost-effectiveness of a task-based weight management group programme - a randomised controlled trial and economic evaluation. *Health Technol Assess* 2016;**20:**79.
33. Owen-Smith A, Donovan J, Coast J. "Vicious circles": the development of morbid obesity. *Qual Health Res* 2014;**24:**1212-20.
34. Owen-Smith A, Donovan J, Coast J. Experiences of accessing obesity surgery on the NHS: a qualitative study. *J Public Health* 2016;**31:**31.
35. Pera PI, Ferrer MCO, Juarez MN, Juarez EN, Soler LM, Matheu CL, et al. Obesity, knee osteoarthritis, and polypathology: Factors favoring weight loss in older people. *Patient Prefer Adher* 2016;**10:**957-65.
36. Shaw RJ, Bosworth HB, Silva SS, Lipkus IM, Davis LL, Sha RS, et al. Mobile health messages help sustain recent weight loss. *Am J Med* 2013;**126:**1002-9.
37. Sturgiss E, Haesler E, Elmitt N, van Weel C, Douglas K. Increasing general practitioners' confidence and self-efficacy in managing obesity: a mixed methods study. *BMJ Open* 2017;**7:**e014314.



For peer review only - http://bmjopen.bmj.com/site/about/guidelines.xhtml

38. Sturgiss EA, Douglas K. A collaborative process for developing a weight management toolkit for general practitioners in Australia-an intervention development study using the Knowledge To Action framework. *Pilot Feasib Stud* 2016;**2:**20.
39. Sturgiss EA, Elmitt N, Haesler E, van Weelc C, Douglas K. Feasibility and acceptability of a physician delivered weight management programme. *Fam Pract* 2017;**34:**43-8.
40. Turner D, Haboubi N. Qualitative and Quantitative Outcomes of a 1:1 Multidisciplinary Weight Management Clinic. *Healthcare* 2015;**3:**429-51.
41. VanWormer JJ, Martinez AM, Cosentino D, Pronk NP. Satisfaction with a weight loss program: what matters? *Am J Health Promot* 2010;**24:**238-45.
42. Yarborough BJ, Stumbo SP, Yarborough MT, Young TJ, Green CA. Improving lifestyle interventions for people with serious mental illnesses: Qualitative results from the STRIDE study. *Psychiatr Rehabil J* 2016;**39:**33-41.
43. Young AS, Cohen AN, Goldberg R, Hellemann G, Kreyenbuhl J, Niv N, et al. Improving Weight in People with Serious Mental Illness: The Effectiveness of Computerized Services with Peer Coaches. *J Gen Intern Med* 2017;**32:**48-55.
44. Zizzi S, Kadushin P, Michel J, Abildso C. Client experiences with dietary, exercise, and behavioral services in a community-based weight management program. *Health Promot Pract* 2016;**17:**98-106.
45. Guzelegun N, Sissoko F, Jeong Ko U, Harrington J, Lanford K, Coulton V, et al. Qualitative opportunities into user experiences of tier 2 and tier 3 weight management services: Public Health England; 2017. https://www.gov.uk/government/publications/weight-management-services-insights-into-user-experiences [Accessed April 2018]
46. Brown 2017 Brown TJ, O'Malley C, Blackshaw J, Coulton V, Tedstone A, Summerbell C, et al. Exploring the evidence base for Tier 3 weight management interventions for adults: a systematic review. *Clin Obes* 2017;**7:**260-72.
47. Campbell R, Pound P, Morgan M, Daker-White G, Britten N, Pill R, Yardley L, Pope C, Donovan J: Evaluating meta-ethnography: systematic analysis and synthesis of qualitative research. Health Technol Assess 2011, 15(43):1–164. 3.
48. Dixon-Woods M, Sutton A, Shaw R, Miller T, Smith J, Young B, Bonas S, Booth A, Jones D: Appraising qualitative research for inclusion in systematic reviews: a quantitative and qualitative comparison of three methods. J Health Serv Res Policy 2007, 12(1):42–47

For peer review only



For peer review only - http://bmjopen.bmj.com/site/about/guidelines.xhtml

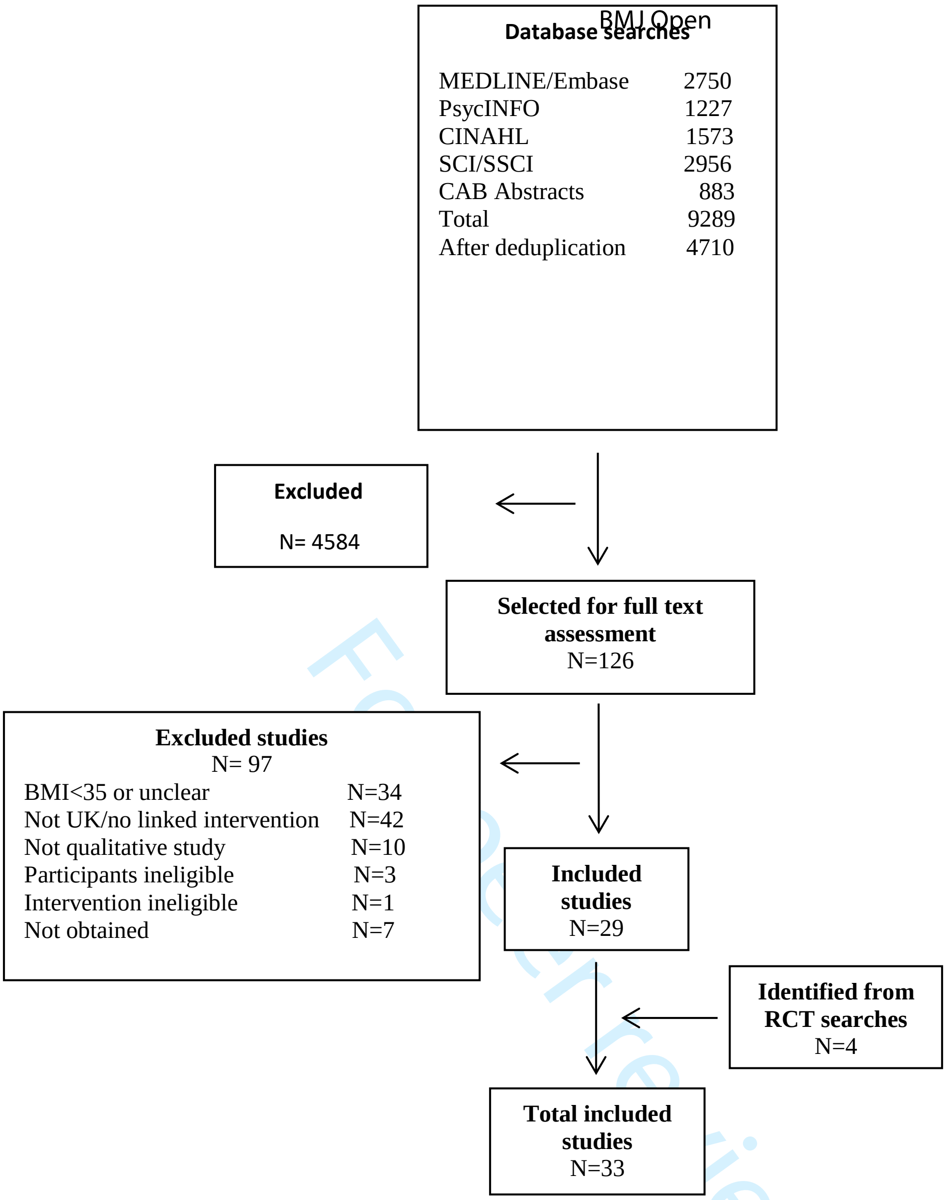


**S1 Figure Flow chart of included studies**

For peer review only

**REVIEW: Qualitative Studies**

**MEDLINE and EMBASE**

Ovid multifile search: http://shibboleth.ovid.com/

Database: Embase <1980 to 2017 Week 31>, Epub Ahead of Print, In-Process & Other Non-Indexed Citations, Ovid MEDLINE(R) Daily and Ovid MEDLINE(R) <1946 to Present>
26th April 2017

For peer review only

**Date of Search 26th April 2017**

1 qualitative research/
2 exp interviews as topic/ use ppez
3 exp interview/ use emez
4 focus groups/ use ppez
5 grounded theory/
6 (qualitative or interview$ or focus group?).tw,kw.
7 (ethno$ or grounded or thematic or realist or interpretive or narrative or discourse analysis or discursive or mixed method$).tw,kw.
8 or/1-7
9 *obesity/
10 morbid obesity/ use emez
11 exp obesity, morbid/ use ppez
12 (obese or obesity).tw,kw
13 or/9-12
14 Weight Loss/ use ppez
15 weight reduction/ use emez
16 (weight adj1 (los$ or reduc$ or maint$ or control$ or manag$)).tw,kw.
17 (reduc$ adj2 (bmi or body mass index)).tw.
18 (reduc$ adj2 (waist adj3 (ratio$ or circumference))).tw.
19 (obesity adj1 manag$).tw,kw
20 anti obesity.tw,kw
21 or/14-20

For peer review only - http://bmjopen.bmj.com/site/about/guidelines.xhtml

22 8 and 13 and 21
23 (obes$ adj3 (morbid$ or severe$ or extreme$)).tw,kw.
24 8 and (10 or 11 or 23)
25 22 or 24
26 25 not (abstract or letter or note or comment).pt.
27 remove duplicates from 26

**PsycINFO**

Ovid: http://shibboleth.ovid.com/

Database: PsycINFO <1987 to April Week 3 2017>

**Date of Search: 26th April 2017**

1 qualitative research/
2 interviews/
3 grounded theory/
4 discourse analysis/
5 ethnography/
6 (qualitative or interview$ or focus group?).tw,kw.
7 (ethno$ or grounded or thematic or realist or interpretive or narrative or discourse analysis or discursive or mixed method$).tw,kw.
8 or/1-7
9 obesity/ or body weight/
10 (obese or obesity).tw,kw
11 9 or 10
12 Weight Loss/ or weight control/
13 (weight adj1 (los$ or reduc$ or maint$ or control$ or manag$)).tw,kw.
14 (reduc$ adj2 (bmi or body mass index)).tw.
15 (reduc$ adj2 (waist adj3 (ratio$ or circumference))).tw
16 anti obesity.tw,kw.
17 (obesity adj1 manag$).tw,kw
18 or/12-17
19 8 and 11 and 18
20 (obes$ adj3 (morbid$ or severe$ or extreme$)).tw,kw.

For peer review only

21 8 and 20

22 "obesity (attitudes toward)"/

23 19 or 21 or 22

**CINAHL**

http://search.ebscohost.com

!981- 25th April 2017

**Date of Search: 25th April 2017**

S1 (MH "Qualitative Studies+")

S2 (MH "Interviews") OR (MH "Semi-Structured Interview") OR (MH "Structured Interview")

S3 (MH "Focus Groups")

S4 (MH "Narratives")

S5 TX qualitative OR TX interview* OR TX focus group*

S6 TX ( ethno* or grounded or thematic ) OR TX ( realist or interpretive or narrative ) OR TX ( discourse analysis or discursive or mixed method* )

S7 S1 OR S2 OR S3 OR S4 OR S5 OR S6

S8 (MH "Obesity") OR (MH "Obesity, Morbid")

S9 (MH "Body Weight")

S10 TX obese OR TX obesity

S11 S8 OR S9 OR S10

S12 (MH "Weight Control")

S13 (MH "Weight Loss")

S14 TX weight N1 los* OR TX weight N1 reduc* OR TX weight N1 maint* OR TX weight N1 control

S15 TX weight N1 manag* OR TX reduc* N2 bmi OR TX reduc* N2 body mass

S16 reduc* N2 waist ratio* OR TX reduc* N2 waist circumference TX

S17 S12 OR S13 OR S14 OR S15 OR S16

S18 (S7 AND S11 AND S17)

S19 (MH "Obesity, Morbid")

S20 TX obes* N3 morbid* OR TX obes* N3 severe OR TX obes* N3 extreme*

S21 S19 OR S20

S22  S7 AND S21

S23 (MH "Attitude to Obesity")

S24  S18 OR S22 OR S23

**Science Citation Index and Social Science Citation Index**

www.webofknowledge.com

1980 -  28th April 2017

**Date of Search: 28th April 2017**

# 1  TS=(qualitative or interview* or focus group)

# 2  TS=(ethno* or grounded or thematic or realist or interpretive or narrative or discourse analysis or discursive or mixed method*).

# 3  #1 OR #2

# 4  TS=(obesity or obese)

# 5   TS=(weight NEAR/1 los*) or TS=(weight NEAR/1 reduc*) or TS=(weight NEAR/1 maint*) or TS=(weight NEAR/1 control*) or TS=(weight NEAR/1 manag*).

# 6  TS=(reduc* NEAR/2 BMI) OR TS=(reduc* NEAR/2 body mass index)

# 7  TS=anti obesity

# 8  TS= (obesity NEAR/1 manag*)

# 9  #5 or #6 or #7 or #8

10  #3 AND #4 AND #9  *))) AND DOCUMENT TYPES: (Article)

**CAB Abstracts**

Ovid search: http://shibboleth.ovid.com/

Database: CAB Abstracts <1984 to 2017 Week 15>

**Date of Search: 26th April 2017**

1  qualitative analysis/

2  qualitative techniques/

3  (qualitative or interview$ or focus group?).tw.

4  (ethno$ or grounded or thematic or realist or interpretive or narrative or discourse analysis or discursive or mixed method$).tw.

5  or/1-4

For peer review only

6 obesity/

7 (obese or obesity).tw.

8 6 or 7

9 weight reduction/

10 (weight adj1 (los$ or reduc$ or maint$ or control$ or manag$)).tw.

11 (reduc$ adj2 (bmi or body mass index)).tw.

12 (reduc$ adj2 (waist adj3 (ratio$ or circumference))).tw.

13 (obesity adj1 manag$).tw

14 anti obesity.tw.

15 or/9-14

16 5 and 8 and 15

17 (obes$ adj3 (morbid$ or severe$ or extreme$)).tw.

18 5 and 17

19 16 or 18

For peer review only

## S1 Table Characteristics of the included qualitative studies

| Study | Aim (as described within the papers) | Condition of Focus | Participants Characteristics | Details of intervention | Qualitative data collection methods |
|---|---|---|---|---|---|
| *First Author*: Bennett<br>*Year:* 2014<br>*Category*: A<br>*Country*: USA | To understand primary care providers' (PCPs) perspectives about their role in the intervention and in their patients' weight loss, thereby providing insights to inform best practices in developing practice-based weight management programmes. | Patients with obesity in their usual care practices. | *Role:* Provider<br>*Number providers interviewed*: 26 PCPs<br>*Providers' characteristics*: 15 female, 11 male, 24 physicians, 2 nurse practitioners, and 20 had internal medicine training. The mean time in practice was 16 years (SD ± 11.7), and mean number of patients in the trial was 11.1 (SD ± 6.8)<br>*Socioeconomic and demographic characteristics*: 15 White, 6 Asian/Pacific Islander, 3 Black, 2 Other | The Practice-based Opportunities for Weight Reduction (POWER) was a 24 month trial that had two intervention groups (by phone and face-to-face) in which weight-loss health coaches (not PCPs) provided education and positive reinforcement. Participants in both intervention arms had access to the same online educational modules, self-monitoring tools and received both automated and individualized e-mails. Participants in the control arm met with a weight loss health coach at the time of randomization and, if desired, after the final data collection visit. They also received brochures along with a list of recommended weight loss websites. | Focus groups |
| *First Author*: Bradbury<br>*Year:* 2015<br>*Category*: A | To explore helpful (and unhelpful) aspects of coaching; | Participants with obesity. | *Role:* Participant<br>*Number of participants*: 58. | Positive Online Weight Reduction (POWeR) is an e-health intervention designed to produce sustainable weight | Interviews |

For peer review only - http://bmjopen.bmj.com/site/about/guidelines.xhtml

| | | | | | |
|---|---|---|---|---|---|
| *Country*: UK | the experiences of POWeR and the accompanying coaching, including what aspects people found most helpful, unhelpful, appealing or unappealing, and what factors seemed to influence whether participants continued to follow POWeR. | | Planning and development stages: 16 participants;<br>Feasibility stage: 23 participants;<br>Community trial 19 participants.<br>*Participants' characteristics*: From the community trial: age range 34-68, Participants were sampled from both the coaching arm (10 female, four male) and Web only arm (four female, one male) and varied in their usage of POWeR.<br>*Socioeconomic and demographic characteristics*: NR<br>*Comorbidities*: NR | management. POWeR consisted of 12 sessions which taught users self-regulation skills in order for them to become their own personal health trainer. Patients were randomized to either usual care, the POWeR website, POWeR accompanied by basic nurse support, or POWeR with regular nurse support. The nurse support was mainly delivered face to face, although telephone and email support could also be provided. | |
| *First Author*: Gudzune<br>*Year:* 2012<br>*Category*: A<br>*Country*: USA | To explore PCPs' usual practices as part of weight counselling to identify how PCPs communicate with their patients about weight loss. | Patients with obesity in their usual care practices | See *Bennett 2014* | See *Bennett 2014* | Focus groups |
| *First Author*: Hunt<br>*Year:* 2014<br>*Category*: A<br>*Country*: UK | To report the characteristics of men participating in a randomised | Men with obesity (BMI > 28kg/m$^2$), age 35–65 at high | *Role:* Participant<br>*Number of participants*: 63 men (who had attended at least six FFIT sessions of the programme). | Football Fans in Training (FFIT) is a men-only, evidence-based, 12-session, weight management and physical activity group programme with subsequent | Focus groups |

| | | | | | |
|---|---|---|---|---|---|
| | controlled trial of a weight management programme designed specifically to attract men, and, secondly, their accounts of why they decided to participate in the programme. | risk of ill-health due to obesity | *Participants characteristics*: No specific data for qualitative analysed participants<br>*Socioeconomic and demographic characteristics*: NR<br>*Comorbidities reported*: NR | minimal-contact weight loss maintenance support delivered free of charge at Scotland's top professional football clubs by community coaches trained in diet, nutrition, physical activity and behaviour change techniques to a standard programme delivery protocol. | |
| *First Author*: Little<br>*Year:* 2017<br>*Category*: A<br>*Country*: UK | To explore patients' expectations of POWeR+, experiences of the POWeR+ programme, experiences of using the POWeR+ website and experiences of nurse support. | Participants with obesity (BMI ≥30kg/m$^2$, or ≥28kg/m$^2$ with comorbidities) from general practice | *Role:* Participant and Provider<br>*Number of providers*: 13 nurses (HCPs who supported POWeR+ were included in qualitative evaluation)<br>*Number of participants:* 31 POWeR+ programme users. 14 remote support (3 low users/11 high users) and 17 face-to-face support patients (2 low users/15 high users).<br>*Participants' characteristics*: 15 female, 16 male, mean age 61 years (range 45-88 years).<br>*Socioeconomic and demographic characteristics*: No specific data for qualitative analysed participants.<br>*Comorbidities reported*: No specific | This is a 24-session web-based weight management intervention consisting of a series of 24 brief maintenance-oriented sessions for up to 6 months and links to encourage patients to continue to use the website to track their weight at least fortnightly until they have formed healthy eating habits that sustain weight management. | Interviews |

For peer review only

| | | | | | |
|---|---|---|---|---|---|
| | | | data for qualitative analysed participants. | | |
| *First Author*: McRobbie<br>*Year:* 2016<br>*Category*: A<br>*Country*: UK | To explore the many components of the WAP. By providing a summary of participant feedback on the overall helpfulness of the programme. | Adults (aged ≥ 18 years) with obesity (BMI of ≥ 30 kg/m$^2$ or a BMI of ≥ 28 kg/m$^2$ plus comorbidities) who wanted to lose weight | *Role:* Participant<br>*Number of participants:* 177. Participants who reported helpfulness of the programme at 12-months follow up; 48 in the nurse arm and 129 in the WAP arm. People who dropped out of treatment were called; only 19 provided a reason for dropping out.<br>*Participants' characteristics*: Not reported<br>*Socioeconomic and demographic characteristics:* Not reported.<br>*Comorbidities:* Not reported | The WAP is a multicomponent programme that includes a range of concrete and verifiable tasks agreed individually with each participant and also includes monthly 'maintenance' sessions that targeted to improve participant motivation, allowing participants to discuss the challenges they have faced since the last session, and to anticipate challenges of the month ahead. | Anonymous feedback questionnaire |
| *First Author*: Yarborough<br>*Year:* 2016<br>*Category*: A<br>*Country*: USA | To assess lifestyle change barriers and facilitators across the first 18 months of study participation and to identify modifiable factors associated with making and maintaining healthy | Adults (aged ≥ 18 years) with obesity (BMI ≥27kg/m$^2$) taking antipsychotic medications (stable on antipsychotic medications for at least 30 days) | *Role:* Participant<br>*Number of participants*: 84. Participants in the control arm were interviewed once; 17 intervention participants were interviewed more than once to ensure that all cohorts were represented in each interview wave.<br>*Participants' characteristics*: Mean age 48.1 (SD ± 10.1), 30 male, 54 | This was a 24-month study of the STRIDE comprehensive weight loss and lifestyle-change intervention that consisted of 24 weekly meetings that targeted readiness to change; included interactive, participant-centred delivery of lifestyle education information along with a 20-min walk; encouraged skills practice, self-monitoring and feedback; and facilitated group interactions and | Interviews |

| | | | | | |
|---|---|---|---|---|---|
| | lifestyle changes in order to inform clinicians and improve the development of future interventions for individuals with serious mental illnesses. | | female. 18 were members of ethnic or racial minorities.<br>*Socioeconomic and demographic characteristics*: 34 married or living with partner, 27 had an income of $30,000 or higher, 18 were college graduate or higher, 28 were retired, unemployed, student, homemaker or temporarily laid off.<br>*Comorbidities*: 34 Schizophrenia, 17 bipolar disorder, 31 affective psychoses, 2 PTSD | support. Intervention participants could consult with interventionists by telephone as needed. | |
| *First Author*: Abildso<br>*Year:* 2010<br>*Category*: B<br>*Country*: USA | To examine physical and psychosocial differences at baseline between completers of and dropouts from a 12-week weight management program; to assess the physical, behavioural, and psychosocial impact on program completers; to | Adults with obesity (BMI ≥ 30kg/m$^2$ alone or a BMI of 25 to 29.9kg/m$^2$ with comorbidities) | *Role:* Participant<br>*Number of participants*: 11<br>*Participants characteristics*: Mean age 46.2 (SD ± 8.5), 8 female, 3 male. Seven were successful program completers (three high weight losers, four moderate weight losers), and four were program dropouts or completers with low weight loss).<br>*Socioeconomic and demographic characteristics*: 7 married, number of children 1.5 (SD ± 1.1)<br>*Comorbidities*: Not reported | Weight loss is encouraged in the weight management program (WMP) through increasing physical activity and decreasing caloric intake. For a $45 monthly co-payment, the WMP benefit during Phase 1 (12 weeks) included assessment and follow-up meetings with an exercise physiologist and registered dietitian, monthly personal training sessions, and periodic phone calls from the insurance agency to track progress. | Interviews |

For peer review only

| | | | | | |
|---|---|---|---|---|---|
| | compare the psychosocial changes of high and moderate weight losers; and to qualitatively explore factors associated with program adherence and weight loss. | | | | |
| *First Author*: Aschbrenner<br>*Year:* 2016<br>*Category*: B<br>*Country*: USA | To explore participants' perceptions and experiences with peer interactions during the lifestyle intervention. | Obese (BMI ≥ 30kg/m$^2$) adults (aged 21 or older) with serious mental illness (diagnosis of schizophrenia, schizoaffective disorder, major depressive disorder, or bipolar disorder) on stable pharmacological treatment | *Role:* Participant<br>*Number of participants*: 17<br>*Participants' characteristics*: No specific data for qualitative analysed participants<br>*Socioeconomic and demographic characteristics*: Not reported<br>*Comorbidities:* Not reported | A 24-week group-based lifestyle intervention that consisted of once weekly 1-hr group weight management sessions facilitated by a psychologist and a public health professional; twice weekly (optional) 1-hr group exercise sessions led by a certified fitness trainer; and mobile technology and use of social media to increase motivation and facilitate self-monitoring and peer-to-peer support outside of in person group treatment or exercise sessions. | Focus groups |

For peer review only

| | | | | | |
|---|---|---|---|---|---|
| *First Author*: Asselin<br>*Year:* 2015<br>*Category*: B<br>*Country*: Canada | To explore how primary care providers incorporate weight management in their practice. | Obesity prevention and weight management at interdisciplinary primary care environment | *Role:* Provider<br>*Number of providers interviewed:* 29<br>*Providers' characteristics*: 7 mental healthcare workers, 7 registered dietitians, 15 registered nurses or nurse practitioners.<br>*Socioeconomic and demographic characteristics:* NR | The 5 As Team (5AsT) study was designed to create, implement and evaluate a flexible intervention to improve the quality and quantity of weight management visits in primary care. 5AsT is a randomized controlled trial on the implementation of a 6-month 5AsT intervention designed to operationalize the 5As of obesity management in primary care. | Interviews and field notes of intervention sessions |
| *First Author*: Asselin<br>*Year:* 2016<br>*Category*: B<br>*Country*: Canada | To describe the intervention, provide continual intervention monitoring and to identify contextual factors that could influence the primary outcome measure. | See *Asselin 2015* | See *Asselin 2015* | See *Asselin 2015* | See *Asselin 2015* |
| *First Author*: Barham<br>*Year:* 2011<br>*Category*: B<br>*Country*: USA | To improve nutrition and physical activity of county employees and promote weight loss (*There was no* | Adults at highest risk for the development of diabetes or who already have been | *Role:* Participant<br>*Number of participants*: Unclear how many of 45 programme participants provided written responses on the end of study programme evaluations. | There were 2 waves of enrolment and 4 intervention groups (up to 12 participants/ group). The intervention was a 3-month program (12 one hour weekly midday group sessions) that targeted healthy diet, physical activity, | Written responses to end of programme participant evaluations |

For peer review only

| | | | | | |
|---|---|---|---|---|---|
| | *qualitative aim stated).* | diagnosed with type 2 diabetes | *Participants characteristics*: No specific data for those who provided written responses<br>*Socioeconomic and demographic characteristics*: Not reported<br>*Comorbidities reported*: Not reported | and stress reduction, followed by a monthly maintenance program with the groups choosing topics that they considered of greatest benefit. Most of the sessions were led by a nurse educator, but individual sessions were also conducted by a dietitian, psychologist, and physical therapist all employees of Upstate Medical University, Syracuse, NY. | |
| *First Author*: Borkoles<br>*Year:* 2016<br>*Category*: B<br>*Country*: UK | To examine the effects of a non-dieting lifestyle intervention approach for women with morbid obesity designed in the framework of the self-determination theory and Health at Every Size on weight maintenance and psychological functioning. | Pre-menopausal females with morbid obesity (BMI $\geq$30kg/m$^2$) older than 18 years of age free of obesity-related diseases and fit for exercise | *Role:* Participant<br>*Number of participants*: 62 (62 interviews at baseline with 36 follow-up interviews, including 12 drop-outs).<br>*Participants' characteristics*: Pre-menopausal women predominantly white Caucasian (97%), with a mean age of 40.2 years<br>*Socioeconomic and demographic characteristics*: most were from the lower SES background, 21% had a degree and 57% left school at 16, 66.1% worked full time and 11% worked part-time, in mainly manual | The WHEEL (Weight, Healthy Eating and Exercise in Leeds) study was a delayed-start, 12 weeks of intensive intervention and 40-week maintenance phase RCT comprising of community-based supervised exercise, lifestyle physical activity and psycho-educational classes on healthy eating and weight management. | Interviews |

For peer review only

| | | | | | |
|---|---|---|---|---|---|
| | | | (29%) and administrative jobs (46.8%)<br>*Comorbidities*: 50% met the International Diabetes Federation metabolic syndrome criteria, 42% reported to have depression often or very often, and 36% used medication related to psychological problems | | |
| *First Author*: Dahl<br>*Year:* 2014<br>*Category*: B<br>*Country*: Norway | To describe how personnel argued for and perceived a residential weight-loss program, to investigate how the participants experienced the program, and to contrast these perspectives. | Adults (between 18 and 60 years old) with obesity (BMI > 40kg/m$^2$ or >35kg/m$^2$ including comorbidities)<br>*Providers*:<br>The personnel were recruited among the staff at the centre | *Role:* Participant and Provider<br>*Number of participants*: 10<br>*Participants' characteristics*: 10 Norwegian participants took part in interviews (8 in focus groups and 2 individually). The age and weight range for these 10 persons were the same as for the total sample (n=30). Age between 22 and 56 years old, their BMI was between 40 and 63, and the group's mean body weight was 144kg<br>*Socioeconomic and demographic characteristics*: NR<br>*Comorbidities:* NR<br>*Number of providers interviewed*: 6<br>*Providers' characteristics*: 2 males and 4 females, considered to be key | This 18-week on-site program intervention took place at the Danish residential weight-loss centre. The program consisted of group-based intensive structured group exercise and educational sessions exercise, diet (individual calorie intake was based on energy calculations for a normal weight person with a sedentary activity level), and an educational program. The educational program comprised lessons about nutrition, monitoring of food intake and instruction in behavioural techniques from cognitive therapy. The personal development component included a minimum of two individual conversations with one of the | Focus groups and interviews |

For peer review only

| | | | | | |
|---|---|---|---|---|---|
| | | | personnel; the director, the administrative executive, and the leaders of the main areas diet, exercise and personal development | psychotherapists, motivational meetings for all participants. | |
| *First Author*: Danielsen<br>*Year:* 2016<br>*Category*: B<br>*Country*: Norway | To explore the experiences of physical activity from a participant perspective prior to, during, and after an intensive inpatient lifestyle modification program, including a high volume of adapted physical activity for the treatment of severe obesity. | Both genders, with a variety in age, degree of obesity (BMI ≥ 40 or 35.0–39.9 with comorbidities), and weight loss during the inpatient stay, as well as variation in weight-loss maintenance and lack of maintenance | *Role:* Participant<br>*Number of participants*: 8<br>*Participants' characteristics*: 5 female, 3 male, aged 35 to 63 years; 6 married/cohabitants and 2 single; BMI ranged from 37 to 60 and body weight from 96 to 185 kg<br>*Socioeconomic and demographic characteristics*: NR<br>*Co-morbidities:* NR | The study was supplementary to a clinical controlled trial with a 1-year prospective follow-up study examining the effects of a 10- to 14-week inpatient lifestyle modification program for subjects with severe obesity. Two to three group-exercise sessions 5 days a week during the inpatient period, each lasting for a minimum of 45 minutes. Aiming to increase compliance, the activity was supervised by exercise scientists and physiotherapists, and the participants were introduced to adapted physical activity and equipment, and exercised together with other individuals with severe obesity. | Interviews |
| *First Author*: Groven<br>*Year:* 2010<br>*Category*: B<br>*Country*: Norway | To show how the training is experienced from a first-person perspective, namely | Female participants with obesity (BMI >35kg/m$^2$) from the weight-loss program in | *Role:* Participants<br>*Number of participants*: 5<br>*Participants' characteristics*: Aged 35-63 years and had been overweight for more than 10 years | Group-based weight-loss program in Norway, a program organized by physiotherapists in the primary health system. Offered to eight women struggling with obesity problems in a particular district of Norway for one | Interviews |

For peer review only

| | | | | | |
|---|---|---|---|---|---|
| | the patients themselves. | Norway | *Socioeconomic and demographic characteristics*: 3 married, 1 divorced and 1 widowed, 1 had a university degree, 2 had a college degree, and 2 had no formal education after high school. The women were at present or previously working in professions providing a service, or care, doing office work, or an academic job on various levels.<br>*Comorbidities*: Not reported | year. Total of 12 exercises were performed throughout the one-hour exercise program. The treatment also included group discussion for 1 hour per month. | |
| First Author: Jackson<br>Year: 2007<br>Category: B<br>Country: UK | To evaluate the effectiveness and acceptability of a specialist health visitor-led weight management clinic in primary care. | Patients with a BMI ≥30 | *Role: Participants*<br>*Number of participants: Unclear how many of 25 questionnaires returned provided written responses*<br>*Participants' characteristics: Not reported*<br>*Socioeconomic and demographic characteristics: Not reported*<br>*Comorbidities: Not reported* | Specialist health visitor-led intervention based on the Jan Felgens '12E2' model. The specialist health visitor sought to inspire participants through a combination of shared goal setting, reflection, problem-solving, positive affirmation and reinforcement. Consultations took place at the health centre and a relaxed, unhurried atmosphere was created. The average consultation time was 20 minutes (range 10–30 minutes), although the first appointment took approximately 1 hour and gave participants time to reflect on their lifestyles and to plan realistic goals | Open ended response options to questionnaire |

For peer review only

| | | | | | |
|---|---|---|---|---|---|
| | | | | for healthy eating and physical activity with the specialist health visitor. | |
| *First Author*: Janke<br>*Year:* 2012<br>*Category*: B<br>*Country*: USA | To gain insight into the patient's experience of comorbid chronic pain and obesity and to improve understanding of the behavioural linkages between the experience of pain, engagement in health behaviours, and obesity treatment outcomes. | Patients attending primary care clinics at a large Midwestern Veteran's Affairs hospital, > 18 years, BMI ≥25; weekly pain at an intensity ≥4 during the prior 3 months; and current diagnosis of a medical complaint associated with persistent pain | *Role:* Participant<br>*Number of participants*: 30<br>*Participants characteristics*: 24 male, 6 female<br>26 were age 50 or older, mean BMI was 36.8 (SD ± 8.9)<br>*Socioeconomic and demographic characteristics*: 22 were white, 20 had greater than a high school education, and 14 were unemployed or disabled while 13 were retired<br>*Comorbidities*: Measured on a scale of 0 to 10 (0 = none, 10 = worst imaginable), average pain intensity was 5.6 (SD ± 1.9) and average pain interference was 3.6 (SD ± 2.1) | The qualitative research project was designed to identify perceptions of those with both overweight/obesity and chronic pain regarding their experience of the course, impact, and treatment history of pain and weight symptoms; factors that might either ease or limit their ability to engage in health-promoting behaviours; and factors that facilitate or hinder engagement in treatments designed to achieve weight and/or pain control. | Focus groups and interviews |
| *First Author*: Jennings<br>*Year:* 2014<br>*Category*: B<br>*Country*: UK | To facilitate weight loss by implementing progressive and sustainable lifestyle changes, based on individually agreed goals over a 1-year | Adults (over 18 years) with obesity (BMI ≥40, or BMI ≥30 with obesity-related comorbidities and/or waist | *Role:* Participant<br>*Number of participants*: 12<br>*Participants' characteristics*: No specific data for qualitative analysed participants<br>*Socioeconomic and demographic characteristics*: No specific data for qualitative analysed participants. | The Fakenham weight management service (FWMS) provides Tier 3 services. This paper was service evaluation and had a cohort design recruited patients to a 1-year programme. | Focus groups |

| | | | | | |
|---|---|---|---|---|---|
| | programme. Focus groups were conducted to explore participants' experiences. | circumference ≥102 cm in men or ≥88 cm in women) | *Comorbidities*: No specific data for qualitative analysed participants. | | |
| *First Author*: Jimenez Lopez<br>*Year:* 2012<br>*Category*: B<br>*Country*: Mexico | To explore the motivations of patients involved in a with reduction programme, by analysing their experiences. | Patients with obesity included in a waiting list for bariatric surgery at a public hospital | *Role:* Participant<br>*Number of participants*: 10<br>*Participants' characteristics*: 2 Male, 8 women, mean age 45.2, mean BMI 41.3<br>*Socioeconomic and demographic characteristics*: NR<br>*Comorbidities*: NR | The dynamic of the intervention included the modification of dietary habits by a psychologic intervention, as recommended by the federal law of obesity management The focus group included ten patients with one investigator as an active observer, and 12 weekly sessions. | Focus groups |
| *First Author*: Kidd<br>*Year:* 2013<br>*Category*: B<br>*Country*: USA | To describe the effect of an 8-week mindful eating intervention on mindful eating, weight loss self-efficacy, depression, and biomarkers of weight in urban, underserved, women | Females (aged 30 years and older) with obesity (BMI ≥30kg/m$^2$) | *Role:* Participant<br>*Number of participants*:12<br>*Participants' characteristics*: Mean weight was 119.7kg (SD ± 16.87), BMI 44.7 (SD ±6.9) , Age ranged from 31–61 and averaged 51.8 years (SD ± 9.1)<br>*Socioeconomic and demographic characteristics*: 7 African American, 5 unemployed, and 4 married; 11 | The study used a mixed methods design. A one group pre-test/ post-test design examined the effect of an 8-week mindful eating intervention on the psychosocial variables and biomarkers. Weekly group sessions lasted 60 to 90 minutes and consisted of education and application of mindful eating principles. | Focus groups |

| | | | | | |
|---|---|---|---|---|---|
| | with obesity; and to identify themes of the lived experience of mindful eating. | | graduated from high school, 6 had college degrees<br>*Comorbidities*: Not reported | | |
| *First Author*: Pera<br>*Year:* 2016<br>*Category*: B<br>*Country*: Spain | To explore the meaning of obesity in elderly persons with knee osteoarthritis and to determine the factors that encourage or discourage weight loss. | Participants with obesity, knee osteoarthritis, and polypathology | *Role:* Participant<br>*Number of participants*: 10<br>*Participants characteristics*: 2 male, 8 female, mean age 67.23 (SD ±7.87), BMI 40.47 (SD ± 4.22), mean weight 92.35 kg (SD ± 8.93)<br>*Socioeconomic characteristics*:: 1 No education, 5 Primary (<5 years), 3 Secondary (<10 years), 1 Higher (>10 years), 2 Housewife, 8 Retired<br>*Comorbidities*: Mean number of co-morbidities 7.02 (SD ± 3.08) | The therapeutic education and functional preadaptation program was a 4-month program consisted of two 40-minute individual visits and three 90-minute group sessions for participants with obesity, knee osteoarthritis and polypathology. The program was designed following the methodology established for this type of program and was based on social learning theories. | Focus group |
| *First Author*: Counterweight<br>*Year:* 2008<br>*Category*: B<br>*Country*: UK | To explore key barriers and facilitators of practice and patient engagement in the Counterweight Programme and to describe key strategies used to | Patients with obesity in routine primary care | *Role:* Participant and Provider<br>*Number of participants*: 37 patients<br>*Number of providers*: weight management advisers (n = 7) in a focus group. In depth interviews were conducted with 15 PNs and 7 GPs across 11 practices.<br>*Participants' and/or providers characteristics*: Not reported | The Counterweight Project was set up to establish and improve obesity management in primary care by implementing an evidence-based weight management intervention that is practice focused. It was developed using theoretical models of behavioural change and, the best available methods from the published evidence. | *Participants*: Interviews and focus groups<br><br>*Providers*: Interviews and focus groups |

For peer review only

| | | | | | |
|---|---|---|---|---|---|
| | address barriers in the wider implementation of this weight management programme in UK primary care. | | *Socioeconomic and demographic characteristics*: Not reported<br>*Comorbidities reported*: Not reported | | |
| *First Author*: Shaw<br>*Year:* 2013<br>*Category*: B<br>*Country*: USA | To evaluate the acceptability, feasibility, and efficacy of daily text messages using regulatory focus theory to help individuals sustain weight loss. | Individuals had to own a mobile phone, be able to receive text messages, and have lost 5% of their body weight since entering the Duke Diet and Fitness Centre | *Role:* Participant<br>*Number of participants*: 60<br>*Participants' characteristics*: No specific data for qualitative analysed participants<br>*Socioeconomic and demographic characteristics*: No specific data for qualitative analysed participants.<br>*Comorbidities*: Not reported | Clients who received treatment at a residential weight loss management program that provides education, practical behavioural strategies, and ongoing support to make long-term changes at the Duke Diet and Fitness Centre (DFC), participated in this study. Participants were randomized to a promotion, prevention, or an attention control text message group after completion of a weight loss program. | Interviews |
| *First Author*: Sturgiss<br>*Year:* 2016<br>*Category*: B<br>*Country*: Australia | To describe the collaborative process used to develop an obesity management programme based on current Australian guidelines for GPs and their patients to | Health professionals involved in obesity management programme based on current Australian | *Role:* Provider<br>*Number of providers*: 38<br>*Providers' characteristics*: 15 GPs, 14 GPs registrar, 5 healthcare consumer representative, 2 representative bodies for chronic illness, 1 dietician, 1 psychologist | The Change Programme is a GP-delivered weight management programme that was developed based on Australian guidelines for the management of obesity in primary healthcare. It is based on one of the pillars of general practice—'patient centeredness'. No directive patient goals | Interviews and focus groups |

For peer review only

| | | | | | |
|---|---|---|---|---|---|
| | be used in primary care. | guidelines for GPs and their patients to be used in primary care | *Socioeconomic and demographic characteristics*: Not reported | were stated and the work was individualized. The programme consists of a GP handbook, patient workbook and computer template. This programme. The patients initially attended appointments every 2 weeks, with less frequent appointments as the programme continued. | |
| *First Author*: Sturgiss<br>*Year:* 2017<br>*Category*: B<br>*Country*: Australia | To assess the acceptability and feasibility of a GP-delivered weight management programme. | *Providers*: Fully qualified GPs from the Australian Capital Territory and New South Wales. | *Role:* Participant and Provider<br>*Number of providers*: 12<br>*Providers' characteristics*: The recruited GPs had an average 12 years of experience (range 4–30 years). The GPs worked in four urban practices and one rural practice.<br>*Number of patient participants*: 15 interviewed<br>*Participants' characteristics*: No specific data for qualitative analysed participants.<br>*Socioeconomic and demographic characteristics*: NR<br>*Comorbidities*: Not reported | See *Sturgiss 2016a* | Interviews |
| *First Author*: Sturgiss<br>*Year:* 2017<br>*Category*: B | To assess the self-efficacy and confidence of GPs | GPs working in 5 different general practices | *Role:* Provider<br>*Number of providers*: 12 | See *Sturgiss 2016a* | Interviews |

For peer review only

| | | | | | |
|---|---|---|---|---|---|
| *Country*: Australia | before and after implementing a weight management programme in their practice. | | *Providers' characteristics*: 12 GPs practised in 5 different general practices, 1 rural and 4 urban, and had between 4 and 30 years clinical experience<br>*Socioeconomic and demographic characteristics*: Not reported | | |
| *First Author*: Turner<br>*Year:* 2015<br>*Category*: B<br>*Country*: UK | To determine both physiological benefits and qualitative information, namely patient satisfaction, associated with the service. | Patients with obesity attending Multidisciplinary Weight Management Clinic (MDWMC) at Aneurin Bevan Hospital, Wales | *Role:* Participant<br>*Number of participants*: 180<br>*Participants characteristics*: 131 female, 49 male, ages ranged between 19 and 74<br>*Socioeconomic and demographic characteristics*: Not reported<br>*Comorbidities*: Not reported | Obesity management in Wales includes the provision of a 1:1 MDWMC. Strategic management of obesity in Wales is guided by The All Wales Obesity Pathway and recommends MDWMCs for people with obesity who have one or more co morbidities and who have tried several interventions without success, or who have complex emotional relationships with food. | Interviews |
| *First Author*: VanWormer<br>*Year:* 2010<br>*Category*: B<br>*Country*: USA | To examine the association between participant and program experiences and satisfaction with a weight loss intervention. | Adults (18 years or older) with obesity (BMI ≥ 32kg/m$^2$) employees of a managed care organization | *Role:* Participant<br>*Number of participants*: 78 (*not clear if all of these provided qualitative information*)<br>*Participants' characteristics*: Mean age 46.9 (SD ± 8.3), 70 female, 8 male, 55 married or living with a partner, 23 not married; body weight | Participants were randomly assigned to either an immediate or delayed start group. The intervention lasted 6 months. During treatment, participants received a telephone-based behavioural weight loss counselling intervention. The intervention included a course manual, behaviour change tools (e.g., food/ activity log, weight chart, pedometer), | Written responses to open ended response options within a questionnaire |

| | | | | | |
|---|---|---|---|---|---|
| | | | (kg) 106.2 (SD ± 16.32), BMI 38.3 (SD ± 5.2)<br>*Socioeconomic and demographic characteristics*: 36 college or graduate degree, 42 had less than college degree<br>*Comorbidities*: Not reported | and up to 10 telephone counselling calls from a registered dietitian and/or health educator. In addition, participants received a home tele monitoring scale and were instructed to weigh themselves daily. | |
| *First Author*: Young<br>*Year:* 2017<br>*Category*: B<br>*Country*: USA | To determine whether computerized provision of weight management with peer coaching is feasible to deliver, is acceptable to patients, and is more effective than in-person delivery or usual care. | Adults (18 years or older) with obesity (BMI > 30 or 28–30kg/$m^2$ with self-reported weight gain of at least 10 pounds in the last 3 months), with diagnosis of schizophrenia, schizoaffective disorder, bipolar disorder, major depressive disorder with psychosis, or posttraumatic | *Role:* Participant<br>*Number of participants*: 48 (24 randomized to WebMOVE and 24 randomized to MOVE SMI)<br>*Participants' characteristics*: No specific data for qualitative analysed participants<br>*Socioeconomic and demographic characteristics*: No specific data for qualitative analysed participants<br>*Comorbidities*: Not reported | Patients were randomized to a computerized weight management with peer coaching (Web- MOVE) or in-person clinician-led weight services, or usual care. Both active interventions offered the same educational content. WebMOVE weekly manualized peer coaching was delivered by phone and emphasized a strengths-based approach with motivational interviewing. MOVE SMI is an in-person weight management program led by a master's level mental health clinician. The program includes 24 sessions (8 individual and 16 group), each lasting 60 min. Usual care consisted of one educational handout on the benefits of weight loss, given to participants after randomization | Interviews |

For peer review only

| | | | | | |
|---|---|---|---|---|---|
| | | stress disorder; with prescribed an antipsychotic medication | | | |
| *First Author*: Zizzi<br>*Year:* 2016<br>*Category*: B<br>*Country*: USA | To explain how these services are perceived and received by participants in a community-based intervention so that specific recommendations can be made to health professionals working with similar populations and in similar settings. | West Virginia public employees' insurance agency weight management program (WMP), which is open to insured members that have a BMI >25 | *Role:* Participant<br>*Number of participants*: 567 (not clear how many provided qualitative data within the questionnaire<br>*Participants' characteristics*: 437 female, 130 male<br>*Socioeconomic and demographic characteristics*: Not reported<br>*Comorbidities*: Self-reported medication usage for 36% heart disease or high blood pressure, 31% anxiety or depression 21% high cholesterol, 12.7% diabetes, 9% sleep apnea | The WMP was a 2-year long benefit, and a $20 monthly co-payment that allowed participants to meet with a registered dietitian, exercise physiologist, and certified personal trainer at various point throughout their time in the program. The majority of individuals in the program also spoke with a health behaviour counsellor via telephone every 6 to 8 weeks. The WMP was offered at approximately 60 approved exercise facilities in West Virginia, such as YMCAs, wellness centres, fitness centres, and physical therapy clinics. | Written responses to open ended response options within a questionnaire |
| *First Author*: Owen Smith<br>*Year:* 2014<br>*Category*: C<br>*Country*: UK | To present a synthesis of data from two qualitative studies in which both the development and the experience of living with morbid obesity in men and | Individuals who met the United Kingdom NICE criteria for a morbid obesity (BMI ≥ 40, or 35 kg/m$^2$ with comorbidity), and | *Role:* Participant<br>*Number of participants*: 31 (Study 1 n = 13; Study 2 n = 18)<br>*Participants characteristics*: 9 males, 3 age group 20–29, 11 age group 30–39, 7 age group 40–49, 9 age group 50–59, 1 60+ age group | The qualitative approach to both studies, to investigate individual experiences of developing and living with morbid obesity. The first study (Study 1) as part of a broader investigation into patients' experiences of implicit and explicit rationing. The core results the second study (Study 2) as part of an ongoing | Interviews |

| | | | | | |
|---|---|---|---|---|---|
| | women were explored in depth. | sought access to treatment for their condition | *Socioeconomic and demographic characteristics*: 15 non manual employment, 5 manual employment, 5 homeworker/carer, 1 retired, 4 unemployed<br>*Comorbidities*: Not reported | longitudinal study investigating how clinicians communicate with patients about the availability of treatment in the context of resource scarcity. | |
| *First Author*: Owen Smith<br>*Year:* 2016<br>*Category*: C<br>*Country*: UK | To focus on experiences of accessing treatment for morbid obesity in primary care. | Patients and providers at a weight management clinic at a general hospital in the South West of England | *Role:* Participant and providers<br>*Number of participants*: 22 patients<br>*Number of providers*: 11<br>*Participants' characteristics*: 7 male, 15 female, 9 age group 20-39, 12 age group 40-59, 1 age 60+<br>*Socioeconomic and demographic characteristics*: 21 white British, 4 professional, 8 other non-manual, 3 manual, 6 unemployed, 1 retired<br>*Comorbidities*: 19 joint pain/mobility issues, 11 depression/other depressive disorder, 10 breathlessness/respiratory difficulties, 9 diabetes, 8 hypertension, 4 sleep apnoea, 4 cardiac problems, 3 fertility issues<br>*Number of providers*: 11 clinicians<br>*Providers' characteristics*: Clinician informants included consultants and | Data collection was undertaken using in-depth interviews with patients and clinicians working in a specialist secondary care facility, and analysis took a constant comparative approach. Patients were followed from before their first consultation in secondary care up to 36 months after referral. | Interviews |

For peer review only

| | | | | | |
|---|---|---|---|---|---|
| | | | three allied medical professionals who worked within the weight management service. *Socioeconomic and demographic characteristics*: Not Reported | | |

Categories: A= Qualitative and mixed-methods studies linked to eligible RCTs, including any qualitative data reported as part of papers reporting quantitative outcomes; B= Qualitative and mixed-methods studies linked to ineligible RCTs and identified non-randomised intervention studies including any reported qualitative data; C= UK-based qualitative studies not linked to any specific interventions that draw on the experiences and perceptions of adults with BMI ≥35 (and/or providers involved in their care). ¥=Studies included in review 2 (long-term randomised and non-randomised studies conducted in UK). BMI= Body Mass Index, calculated weight (kg) / height (m2)

For peer review only

# Enhancing transparency in reporting the synthesis of qualitative research: ENTREQ

## ENTREQ Statement: content and rationale

The ENTREQ statement consists of 21 items grouped into five main domains: introduction, methods and methodology, literature search and selection, appraisal, and synthesis of findings (Table 1). For each item, a descriptor and examples are provided. Below we present a rationale for each domain and its associated items.

Table 1

**Enhancing transparency in reporting the synthesis of qualitative research: the ENTREQ statement**

| No | Item | Guide and description | |
|---|---|---|---|
| **1** | Aim | State the research question the synthesis addresses. | See Page 3 |
| **2** | Synthesis methodology | Identify the synthesis methodology or theoretical framework which underpins the synthesis, and describe the rationale for choice of methodology *(e.g. meta-ethnography, thematic synthesis, critical interpretive synthesis, grounded theory synthesis, realist synthesis, meta-aggregation, meta-study, framework synthesis).* | See Page 4 |
| **3** | Approach to searching | Indicate whether the search was pre-planned (*comprehensive search strategies to seek all available studies)* or iterative (*to seek all available concepts until they theoretical saturation is achieved)*. | See Page 3/4 |
| **4** | Inclusion criteria | Specify the inclusion/exclusion criteria *(e.g. in terms of population, language, year limits, type of publication, study type).* | See Page 3 |
| **5** | Data sources | Describe the information sources used (e.g. *electronic databases (MEDLINE, EMBASE, CINAHL, psycINFO, Econlit), grey literature databases (digital thesis, policy reports), relevant organisational websites,* | See Page 3 |

| No | Item | Guide and description | |
|---|---|---|---|
| | | *experts, information specialists, generic web searches (Google Scholar) hand searching, reference lists)* and when the searches conducted; provide the rationale for using the data sources. | |
| **6** | Electronic Search strategy | Describe the literature search *(e.g. provide electronic search strategies with population terms, clinical or health topic terms, experiential or social phenomena related terms, filters for qualitative research, and search limits)*. | See Page 3 and S1 Appendix |
| **7** | Study screening methods | Describe the process of study screening and sifting *(e.g. title, abstract and full text review, number of independent reviewers who screened studies).* | See Page 3/4 |
| **8** | Study characteristics | Present the characteristics of the included studies *(e.g. year of publication, country, population, number of participants, data collection, methodology, analysis, research questions).* | See Page 6/7 and S1 Table |
| **9** | Study selection results | Identify the number of studies screened and provide reasons for study exclusion *(e,g, for comprehensive searching, provide numbers of studies screened and reasons for exclusion indicated in a figure/flowchart; for iterative searching describe reasons for study exclusion and inclusion based on modifications t the research question and/or contribution to theory development).* | See Figure 1, page 5 |
| **10** | Rationale for appraisal | Describe the rationale and approach used to appraise the included studies or selected findings *(e.g. assessment of conduct (validity and robustness), assessment of reporting (transparency), assessment of content and utility of the findings).* | See Page 5 |

| No | Item | Guide and description | |
|---|---|---|---|
| **11** | Appraisal items | State the tools, frameworks and criteria used to appraise the studies or selected findings *(e.g. Existing tools: CASP, QARI, COREQ, Mays and Pope* [25]*; reviewer developed tools; describe the domains assessed: research team, study design, data analysis and interpretations, reporting).* | See Page 5 |
| **12** | Appraisal process | Indicate whether the appraisal was conducted independently by more than one reviewer and if consensus was required. | See Page 5. Two reviewers initially assessed quality of included studies using the criteria proposed by Toye et al. During subsequent group discussions we continued to discuss and reflect on key aspects of quality. |
| **13** | Appraisal results | Present results of the quality assessment and indicate which articles, if any, were weighted/excluded based on the assessment and give the rationale. | Please see detail provided on pages 22-23 |
| **14** | Data extraction | Indicate which sections of the primary studies were analysed and how were the data extracted from the primary studies? *(e.g. all text under the headings "results /conclusions" were extracted electronically and entered into a computer software).* | See Page 4 ans S1 Table |
| **15** | Software | State the computer software used, if any. | N/A |
| **16** | Number of reviewers | Identify who was involved in coding and analysis. | See Pages 4 |
| **17** | Coding | Describe the process for coding of data *(e.g. line by line coding to search for concepts).* | See Page 4 |
| **18** | Study comparison | Describe how were comparisons made within and across studies *(e.g. subsequent studies were coded into pre-existing concepts, and new concepts were created when deemed necessary).* | See Page 4 and S1 Table |

| No | Item | Guide and description | |
|---|---|---|---|
| **19** | Derivation of themes | Explain whether the process of deriving the themes or constructs was inductive or deductive. | See page 4 |
| **20** | Quotations | Provide quotations from the primary studies to illustrate themes/constructs, and identify whether the quotations were participant quotations of the author's interpretation. | See Results section |
| **21** | Synthesis output | Present rich, compelling and useful results that go beyond a summary of the primary studies (e.g. *new interpretation, models of evidence, conceptual models, analytical framework, development of a new theory or construct).* | See Results and discussion section. |

For peer review only

BMJ Open

# The acceptability and feasibility of weight management programmes for adults with severe obesity: A qualitative systematic review



| | |
|---|---|
| Journal: | *BMJ Open* |
| Manuscript ID | bmjopen-2019-029473.R1 |
| Article Type: | Research |
| Date Submitted by the Author: | 09-Jul-2019 |



| | |
|---|---|
| Complete List of Authors: | Skea, Zoë; Universit of Aberdeen, Health Services Research Unit<br>Aceves-Martins, Magaly; University of Aberdeen, Health Services Research Unit<br>Robertson, Clare; University of Aberdeen, Health Services Research Unit<br>De Bruin, M; University of Aberdeen,<br>Avenell, Alison; University of Aberdeen, Health Services Research Unit |



| | |
|---|---|
| <b>Primary Subject Heading</b>: | Public health |
| Secondary Subject Heading: | Nutrition and metabolism |
| Keywords: | QUALITATIVE RESEARCH, PUBLIC HEALTH, NUTRITION & DIETETICS |
| | |


SCHOLARONE™
Manuscripts

# The acceptability and feasibility of weight management programmes for adults with severe obesity: A qualitative systematic review

**Skea ZC, Aceves-Martins M, Robertson C, de Bruin M, Avenell A, and the REBALANCE team ***

**Author: Dr Zoë Christina Skea**

**Corresponding author: Dr Zoë Christina Skea, Health Services Research Unit, 3rd Floor, Health Sciences Building, University of Aberdeen, Aberdeen, AB25 2ZD, Tel: +44 (0) 1224 438153. email:z.skea@abdn.ac.uk. Fax: +44 (0) 1224 438165.**

**Co-authors: Dr Magaly Aceves-Martins, University of Aberdeen, Aberdeen, UK; Ms Clare Robertson, University of Aberdeen, Aberdeen, UK; Professor Marijn de Bruin, University of Aberdeen, Aberdeen; UK, and Radboud Institute for Health Sciences, The Netherlands; Professor Alison Avenell, University of Aberdeen, Aberdeen, UK.**

**Key words: Qualitative research; Severe obesity; Weight management programmes.**

**Word count: 7165 words**

## Abstract

### Objectives

To improve our understanding of the acceptability of behavioural weight management programmes (WMPs) for adults with severe obesity.

### Design

A systematic review of qualitative evidence.

### Data Sources

Medline, Embase, PsycINFO, CINAHL, SCI, SSCI and CAB abstracts were searched from 1964-May 2017.

### Eligibility Criteria

Papers that contained qualitative data from adults with BMI ≥ 35kg/m$^2$, (and/or the views of providers involved in their care) and considered issues about weight management.

### Data extraction and synthesis

Two reviewers read and systematically extracted data from the included papers which were compared, and contrasted according to emerging issues and themes. Papers were appraised for methodological rigour and theoretical relevance using Toye's proposed criteria for quality in relation to meta-ethnography.



For peer review only - http://bmjopen.bmj.com/site/about/guidelines.xhtml

**Results**

33 papers met our inclusion criteria from seven countries published 2007-2017. Findings were presented from a total of 644 participants and 153 programme providers.

Participants described being attracted to programmes that were perceived to be novel or exciting, as well as being endorsed by their health care provider. The sense of belonging to a group who shared similar issues, and who had similar physiques and personalities, was particularly important and seemed to foster a strong group identity and related accountability. Group based activities were enjoyed by many and participants preferred WMPs with more intensive support. However, some described struggling with physical activities (due to a range of physical co-morbidities) and not everyone enjoyed group interaction with others (sometimes due to various mental health co-morbidities). Although the mean BMI reported across the papers ranged from 36.8 - 44.7kg/m$^2$, no quotes from participants in any of the included papers were linked to specific detail regarding BMI status.

**Conclusions**

Although group-based interventions were favoured, people with severe obesity might be especially vulnerable to physical and mental co-morbidities which could inhibit engagement with certain intervention components.

**Strengths and limitations of this study**

- This is the first synthesis of key findings from qualitative studies exploring views of Weight Management Programmes for adults with severe obesity (body mass index ≥35kg/m$^2$).
- Qualitative studies have a key role to play in understanding how factors facilitate or hinder the effectiveness of interventions, and how the process of interventions are perceived and implemented by users.
- Across the 33 papers, specific participant characteristics were inconsistently and poorly reported (if at all).
- Although the mean BMI reported across the papers ranged from 36.8 - 44.7kg/m$^2$, no quotes from participants in any of the included papers were linked to specific detail regarding BMI status.



For peer review only - http://bmjopen.bmj.com/site/about/guidelines.xhtml

**Introduction**

There has been a continued increase in body mass index ≥35kg/m$^2$ (which we call here 'severe obesity' in adults worldwide. As BMI increases, obesity-related comorbidities, social, psychological and economic consequences increase, with the potential need for greater support for help with weight loss. In the UK, having severe obesity, with or without comorbidities, may be a referral criterion for Tier 3 specialist weight management services in the obesity pathway, prior to Tier 4 services for bariatric surgery {1,2}. Effective weight loss services may reduce the need for bariatric surgery, and could also increase the effectiveness of subsequent bariatric surgery {3}.Current NICE and SIGN guidance on weight management for obesity does not distinguish between obesity (BMI 30 to <35kg/m$^2$) and severe obesity (BMI ≥35kg/m$^2$); and public health guidance excludes evidence on weight loss programmes for obese people with co-morbidities {1,4,5} This implies that Tier 3 services are being created and money is being spent without an appropriate evidence synthesis that clarifies what works for people with severe obesity (and their co-morbidities).

Qualitative studies have a key role to play in understanding how factors facilitate or hinder the effectiveness of interventions, and how the process of interventions are perceived and implemented by participants. This qualitative evidence synthesis was conducted as part of a larger systematic review funded by the UK's National Institute for Health Research Health Technology Assessment Programme {6} and aimed to improve our understanding of the feasibility and acceptability of non-surgical weight management programmes (WMPs) for adults with severe obesity and programme providers. Previous qualitative evidence syntheses have been undertaken {7,8} but these have not focussed on WMPs that are designed for or include people with severe obesity.

Our broad initial research questions included "What is it like to engage with (or be a provider of) weight loss interventions for adults with severe obesity?" and "What is it about interventions for adults with severe obesity that makes them helpful or unhelpful? Our review also considered issues around what might motivate people to decide to engage in such programmes.

This paper focuses on the main themes that emerged from the qualitative synthesis of included studies. These themes shed light on 1) motivating factors for engagement; 2) components of WMPs participants described valuing; and 3) general challenges for engagement.



For peer review only - http://bmjopen.bmj.com/site/about/guidelines.xhtml

## Methods

### *Searching and identification of relevant studies*

A systematic search was conducted in June 2016 and updated during April/May 2017 for published papers that contained qualitative data from adults with BMI ≥ 35kg/m$^2$ (and/or the views of providers involved in their care) and considered issues relating to weight management (See S1 Appendix for search strategies and S1 ENTREQ Checklist). Two researchers (ZCS and MAM) independently screened titles, abstracts and selected full text papers. Where consensus could not be reached regarding eligibility, a discussion at a research team meeting took place.

We included studies that fitted into the following broad categories:

A. Qualitative and mixed-methods studies linked to eligible RCTs (from our other review), including any qualitative data reported as part of papers reporting quantitative outcomes;
B. Qualitative and mixed-methods studies linked to ineligible RCTs and identified non-randomised intervention studies including any reported qualitative data;
C. Qualitative studies not linked to specific interventions that drew on the experiences and perceptions of adults with BMI ≥35kg/m$^2$ (and/or providers involved in their care) providing they reported data specifically relating to views/experiences of strategies for weight loss.

### *Analysis and synthesis*

There are several approaches that can be used for synthesising the findings of qualitative studies.{9,10} Whilst being aware of the differing philosophical stances underlying various approaches to qualitative synthesis, we chose to adopt a pragmatic approach to our work in this area, which specifically aims to synthesise data that are relevant to informing policy and practice.{8} Our pragmatic approach drew on a ‘realist’ perspective{10,11} as we were concerned with trying to find out not only ‘what works’ in terms of weight management for this group of adults and intervention providers, but also ‘for whom, and under what circumstances’. At the same time, our approach was informed by and used aspects of review methods such as thematic synthesis{12,13} and analytical approaches developed from methods of inquiry such as grounded theory.{13}



For peer review only - http://bmjopen.bmj.com/site/about/guidelines.xhtml

In order to collate and synthesise the available primary research, two authors (ZS, MAM) each read and systematically extracted data from the included papers, shared notes and discussed study findings and interpretations during a series of group meetings. The papers were initially organised according to the categories described above but, as inductive analysis progressed, papers were grouped, compared, and contrasted according to emerging issues and themes. We used a data extraction form, which summarised the main findings and original authors' discussion points and to note our own critical and interpretive comments on the papers. We then used these to facilitate the process of comparing and contrasting themes both within and across papers in order to develop cumulative insights into the mechanisms that are likely to impact on decisions to join and decisions to stay in or drop out of WMPs.

### *Study quality*

The retrieved publications were appraised for methodological rigour and theoretical relevance independently by two reviewers using Toye's recently proposed criteria for quality in relation to meta-ethnography.{14} They suggest two core facets of quality for inclusion in syntheses of qualitative evidence, namely (1) Conceptual clarity: how clearly has the author articulated a concept that facilitates theoretical insight; (2) Interpretive rigour: what is the context of the interpretation; how inductive are the findings; has the interpretation been challenged? Two reviewers made notes regarding quality and results were compared and discussed.

### *Patient and Public Involvement*

The REBALANCE Advisory Group included a mix of professional and lay members identified through team contacts (a clinician; dietician; policymaker; and 3 lay people who had all experience of severe obesity and use of related services) who offered advice throughout various stages of this project including during initial discussions around the choice of appropriate research questions to attempt to answer and areas of interest for this review, and our other suite of reviews which considered issues around intervention effectiveness and cost-effectiveness {6}. Results were disseminated at a final project meeting in 2018 at which the Advisory Group were present.

## Findings



For peer review only - http://bmjopen.bmj.com/site/about/guidelines.xhtml

***Description of studies***

The database search produced 4710 abstracts (See S1 Figure for the PRISMA diagram providing information on the flow of studies through the review). Four additional papers were identified from included RCTs. In all, 33 papers met our inclusion criteria.{15-47}

The focus and key study characteristics of the 33 papers are outlined in S1 Table. The identified papers reported research conducted in seven countries (USA n=12; UK n=11; Norway n=3; Spain n=1; Canada n=2; Australia n=3; Mexico n=1), published between 2007 and 2017, and seven papers were linked to broader intervention studies: {18,19,21,28,40,41,42} Seven papers were classed as Category A; 24 Category B; and 2 Category C. As can be seen from S1Table, the studies had varying aims, but all offered insights into stakeholder's perceptions of weight loss strategies and programmes.

Although all the included papers provided some qualitative data for analysis, five of these provided qualitative data in the form of responses to open-ended survey questions within structured questionnaires.{20,30,35,44,47 } Of those studies that used qualitative methods to collect their data, findings were presented from a total of 644 participants and 153 programme providers (mostly from interviews or focus group sessions).

Across the 33 papers, specific participant characteristics were inconsistently and poorly reported (if at all). Only 16 out of 33 papers provided any details. In terms of sex, information for 588 participants (out of 644 of those who specifically took part in qualitative evaluations) was provided – 372 female; 216 male. Age was reported across 15 papers, with the range being 19-88 years. Six of these papers provided mean age with the range being 40.2–67 years. BMI for those involved in qualitative evaluations was reported in nine papers. Of those that provided a mean, this ranged from 36.8-44.7kg/m$^2$. Only four papers gave details of participants' ethnicity; from 188 participants, 35 were reported as being from ethnic or racial minorities. Furthermore, 14 papers specifically stated that study participants had a range of additional physical and/or serious mental health problems (e.g. osteoarthritis, chronic pain, schizophrenia, post-traumatic stress disorder). It was also apparent across other included papers from quotes and/or author comments that many participants had a range of similar comorbidities.

Although no included papers provided qualitative data from those who had been invited to join a programme, but had declined to take part at recruitment stage, some papers reported including



For peer review only - http://bmjopen.bmj.com/site/about/guidelines.xhtml

participants who had not fully engaged with programme activities (being described as 'low users'; 'quitters' or 'drop outs').{15,22,23,34}.

The WMPs varied in terms of the types and formats of support offered. Some programmes involved predominantly face to face interaction and activities with other participants and/or programme staff{22,25,27,29,30,31,32,33,38,43,45} whereas others involved more remote forms of support (e.g. e-mail, telephone, text contact).{39,44} Other studies included and evaluated a mix of formats that also varied in intensity.{15,17,21,23,28,34,35,40,41,42,46,47 }

Programmes incorporated a variety of tools and techniques designed to support behaviour change and to help people lose weight, e.g. tools such as diet diaries;{22,35} workbooks;{40,41,42} pedometers;{34,35,46} food logs; {15,45} conversation maps;{20} interactive monitoring devices;{44} social media group interaction; {17} daily text messages;{39} buddying;{35}. They also included a range of behaviour change techniques (BCTs) and/or psychological support {18,19,24}such as goal setting;{30,31,34} motivational interviewing;{31} mindfulness;{33} self-determination theory based support;{22} regulatory focus theory;{39} self-regulation and cognitive behavioural techniques; {15,21,25,28,29,31,34,40,41,42}. Readiness to change and self-monitoring and feedback was also included{45}along with psychotherapeutic sessions;{32} emotional freedom therapy;{31}; neurolinguistic programming;{31} solution focussed therapy;{31} social learning theories;{38}

**Findings from the synthesis – participants**

This section of the paper discusses the views of participants who chose to engage with WMPs. It considers motivating factors for their initial engagement; components of the WMPs that they described valuing; and then outlines more critical reflections and challenges for engagement (See S1 Conceptual diagram for an illustrative representation of key issues). The subsequent section of the paper discusses similar issues from the perspective of WMP providers.

***Motivating factors for engagement in WMPs***

Several papers provided insights into what had motivated prospective participants to take part in a specific WMP.{22,24,25,29,31,33,45}Important 'push' factors were sometimes personal to participants, for example expressing a desire to do something about their weight/poor physical fitness for themselves (e.g. as a result of growing health concerns and/or recent personal health scares) and also feelings of accountability to their families (e.g. stating that they wanted to be more



For peer review only - http://bmjopen.bmj.com/site/about/guidelines.xhtml

engaged in activities with family members, as well as being there for family for as long as possible). Others recounted familial past experiences of health problems due to obesity or their own sudden and rapid weight gain due to mental health medication. For example:

***Recent personal health scares***

> *"I was told I was at risk of becoming diabetic." (No sample characteristics provided) {31}*

***Feelings of accountability to their families***

> *"I've had two kids in the last three years… that was part of the motivation… just getting fitter for my kids…I need to be aboot* [about] *for as long as possible" (Male).{29}*

***Familial past experiences of health problems due to obesity***

> *"My dad was a big guy and he developed diabetes, and he had to have surgeries and all kinds of stuff. I don't want to do that later in life." (intervention arm; no other sample characteristics provided). {45}*

***Sudden and rapid weight gain due to mental health medication***

> *"When I went on Zyprexa I gained a hundred pounds, very quickly. And that was really frustrating for me." (control arm; no other sample characteristics provided). {45}*

In addition to describing motivating factors that could be classed as personal, some participants described motivators that were apparently related to certain aspects of the programme intervention itself, for example, because it was perceived as being endorsed as credible by health professionals; perceived as being novel and exciting in some key way, and also because it provided an opportunity to engage with the intervention in a place that was valued.{24,25,29}

> *"When I first went in there I thought this is great. I am going to diet at my doctor's surgery. Knowing that it was at my doctor's surgery gave me a big 'oof'." (no sample characteristics provided). {24}*

Although one paper highlighted that decisions to join a WMP were sometimes difficult and that some participants had expressed initial apprehension and reservations around taking part, {29} no included studies provided data about those who were invited to join but declined to take part at recruitment stage.



For peer review only - http://bmjopen.bmj.com/site/about/guidelines.xhtml

**Components of lifestyle programmes participants described liking or valuing**

We examined various aspects of WMPs that participants described valuing. In doing so, we were interested in the range of factors that might motivate those participants to join in the first place, continue to stay in the programme and also the factors that they described as having assisted them to change aspects of their behaviour or ways of thinking. All but two papers were set within the context of a WMP. The two included papers that were not linked to a specific intervention{36,37} also provided data regarding perceptions of weight loss strategies and engagement in diet and lifestyle programmes and were useful in this context. Unsurprisingly, there was variation in terms of what participants described as valuing within their WMP, demonstrating that a one size fits all approach is unlikely to be appropriate. We noted some key recurring themes in terms of what participants valued, and we grouped these around aspects that relate to a) the overall setting or style of the programme; b) the people (both other participants and health professionals/support staff) within the programme setting; c) the type of interaction/support offered; d) dietary elements; e) physical activities; and d) programme tools and techniques designed to support behaviour change. These are discussed below.

***a) The overall setting or style of the programme***

The overall setting of the programme was important for motivating people to decide to engage and also seemed important for motivating them to stay in and keep going with the various intervention activities. Some participants described their programmes as being exciting or novel in that they perceived them to be different to interventions they had tried previously. For example, being focussed on physical activity rather than dieting{22} or being focussed on changing overall attitudes towards eating rather dieting *per se*;{33,41}. An important consideration was the extent to which they could 'relate' to the nature of the programme (including how it was presented to them at recruitment) and how well it appeared to match with their own identities and values:{22,29,33,37}

> *"…the main thing that drew us to it was because it's* [at a football club]*" (Male). {29}*

> *"I always think somebody approaching you one-on-one is better. They can post all the weight loss you know pamphlets out there…I was hooked right away because somebody took the time to really explain it and take her time to do that." (Female). {33}*

Several participants also positively contrasted their overall perceptions of the WMPs with previous negative views towards other WMPs they had engaged with (e.g. WMPs which were perceived as



For peer review only - http://bmjopen.bmj.com/site/about/guidelines.xhtml

being too ‘feminine’ or in some ways humiliating and embarrassing, or being perceived to be overly preoccupied with dieting;{22,23,27,30,31,37}

> *“If you go to a slimming class you feel that you’ve made a fool of yourself or you get weighed and you’ve put on half a pound or a pound, and then you don’t want to go back the next week so you don’t go back.” (Coaching group arm; no other sample characteristics provided). {23}*

> *“Well, I think it’s (WHEEL) appealed to me because I won’t be dieting…I am obsessed with dieting me.” (Female) {22}*

> *“…spent many useless years at weight watchers with various leaders but never felt confident and in control or had the motivation I have now.” (No sample characteristics provided). {30}*

***b) The importance of the people within the programme setting (for fostering a sense of accountability)***

A strong recurring theme was the value participants placed on perceiving themselves to be part of a like-minded group of individuals – individuals that faced similar issues, and who had similar physiques and personalities.{17,20,22,23,27,29,32} For example:

> *“I do not feel so ashamed of my body here. We are all in the same situation, you see, which is really nice” (Female). {27}*

These perceptions seemed to foster a strong group identity and related ‘accountability’ or responsibility in participants. Something that was apparently important for people in terms of motivating them to stick with the programmes and to not let their fellow participants down by dropping out or not sustaining behaviour changes:{15,17,22,23,29,33,34,35,45}

> *“So, you didn’t want to disappoint yourself, but you didn’t want to disappoint … your friends now either." (No sample characteristics provided). {33}*

Many participants also discussed the importance of their interactions with health care staff within the programmes.{15,22,23,25,27,30,31,32,33,35,38,41,43,47} They seemed to value the positive,



For peer review only - http://bmjopen.bmj.com/site/about/guidelines.xhtml

friendly, and non-judgemental encouragement received and they also discussed feeling accountable to programme staff which helped with motivation. These aspects seemed to act as positive ‘pulls’ in terms of staying in the intervention and helping to sustain behaviour change:

> *“I think I just like talking to you [programme leader]. And I suppose I feel that if I don’t do it [the programme] then I’m letting you down” (Female).{22}*

> *“She is my motivator… and she makes me keep a record of my diet” (Female). {27}*

***c) The type of interaction/support offered***

Although not universal, many described particularly valuing the social interactivity of group based programme activities and also fairly intensive support from/interaction with programme staff.{15,17,22,23,26,29,30,32,33,34,38,45,46} This appeared to function strongly as a motivator to maintain engagement with the WMPs by fostering feelings of accountability and by helping to ensure the achievement of pre-set goals:

> *“Oh God I haven’t done what I should of done and I promised to do it and I know that isn’t what’s supposed to spur you on but it I think it does” (Regular support group; no other sample characteristics provided).{23}*

> *“[discussing feedback from programme staff]…great encouragement when the results are positive and a way to improve if the results are not so good.” (No sample characteristics provided). {30}*

Participants discussed appreciating when the timing of support offered was flexible and could fit around their needs,{23,33,35} and several wanted more support than was offered within the programmes (e.g. more frequent contact and for a longer duration than the programme currently allowed).{23,34,44,47} Many also expressed concern about support ending post-intervention{22,23,27,33,39,45} with the suggestion that diminishing intensity of programme activities and/or programme cessation could cause problems for maintaining behaviour change patterns if group interaction and support were key parts of it:

> *“I cannot do it without her support, it just wouldn’t work” (Female). {27}*



For peer review only - http://bmjopen.bmj.com/site/about/guidelines.xhtml

Some WMPs involved predominantly face to face interaction and activities with other participants and/or programme staff. {22,25,27,29,30,31,32,33,38,43,45} In contrast, others involved more remote forms of support (e.g. e-mail, telephone, text contact). {39,44} Some studies included and evaluated a mix of formats that also varied in intensity. {15,17,21,23,28,34,35,40,41,42,46,47} Many participants discussed valuing the social interactivity of the in person group based activities{17,22,23,29,33,34,45} and, where it was discussed and compared, participants tended to value and desire human contact over more remote forms of support. {34,44} This preference seemed to be linked to incentivising people to stay committed to the various programmes and was also apparently important in terms of making participants feel accountable to a likeminded group of individuals.

***d) Dietary elements***

Some WMPs provided detailed dietary advice regarding food choices, whilst others specifically described interventions as 'non-dietary' (nevertheless, incorporating behavioural change techniques to support attitudinal changes towards food and eating patterns). Although views were sometimes mixed, participants tended to describe valuing the flexibility and variety of diet format. {22,33,34,38} This seemed important in terms of helping them to 'normalise' and stabilise their eating habits, particularly as many had attempted diets over a period of many years (without success) leading them to develop negative and unhealthy relationships towards food.{22,33,34,38}

> *"The other programs told you not to eat this or that and you were afraid to go back if you hadn't lost weight and …they tell you that you can eat everything but you yourself have to control the amount…You make up the diet every day and that's very motivating" (Female).{38}*

***e) Physical activities***

All of the WMPs incorporated some attention to increasing physical activity. Whilst clearly some participants described struggling to engage in exercise for a variety of reasons, many participants described the positive psychological and physical benefits they experienced from exercising.{17,22,27,31,45}

> *"When I first started I could hardly walk…now I can walk 300-400 yards…if this project has done nothing else it has helped me to walk (no sample characteristics provided." (No sample characteristics provided).* {31}



For peer review only - http://bmjopen.bmj.com/site/about/guidelines.xhtml

For peer review only

When it was offered as part of the WMP, participants also discussed valuing the flexibility of being able to choose from a variety of exercise formats and approaches. {22,34}

***f) Programme tools and behaviour change techniques designed to support behaviour change***

Although not universally popular, {15,22,34,44,45} participants described the incorporation of tools, such as food logs, goal setting, regular text messages, tele-monitoring devices and conversation maps as being motivating, and also helpful for the purposes of education and learning, describing how they helped to facilitate self-awareness of and reflection on eating and other behaviour patterns. {15,20,34,35,39,44,45,46,47}

> *"I found it to be very enlightening. It made me start to look at foods differently It has given me a more conscious outlook on how to control my diabetes and the importance of exercise." (No sample characteristics provided). {20}*

> *"What really helped me was having somebody go over the food log every day. That was the big thing." (No sample characteristics provided). {15}*

Participants discussed the positive psychological changes they experienced with regards to their relationship to food/body image, which seemed to relate to the BCTs employed within some of the WMPs (e.g. mindfulness and self-determination theory based support).{15,22,25,33}

**General challenges for engagement in WMPs**

Despite the numerous positive comments from within the data with regard to programme engagement, participation was not straightforward for everyone who took part. General challenges resulting in decreased engagement (or success) related to a number of factors. Sometimes, these involved the timing of clinic appointments;{35} cost of travel to appointments;{31,46} general low self-efficacy;{24} family members not being on board, such that behavioural changes were difficult to sustain;{32,45} whereas others described factors which could be described as life getting in the way (e.g. holidays, social events, bad weather as disincentive to exercise). {45}

It was apparent that participants experienced a range of comorbidities, including some serious mental health issues. {16,17,34,35,36,37,44,45,46} Sometimes these specific illnesses presented



For peer review only - http://bmjopen.bmj.com/site/about/guidelines.xhtml

challenges for motivation and continuing engagement, for example, feeling too ill to focus on weight/feeling too ill to care or to be motivated:{31,34,37,38,45}

> *"Because of the ME* [myalgic encephalopathy] *I'm sleeping fifteen or more hours a day, and so exercise is out of the question because I can't even walk to the end of the road." (Female).{36}*

**Critical reflections on specific components of WMPs**

***The type of interaction/support offered***

The recurring theme of valuing the social interactivity of group-based programme activities was not universally valued by all, with some describing a reluctance to discuss issues within a group setting.{17,25,26,38,43,46} This was perhaps particularly pertinent in studies where participants had additional mental health issues:

> *"I know the importance of the program is to be together, but at the beginning you don't know these people, some of us have problems interacting with people we don't know." (No sample characteristics provided. {17}*

> *"It's just I don't like to be around people." (No sample characteristics provided). {46}*

> *"I prefer to talk in private as I suffer from panic attacks." (No sample characteristics provided). {43}*

One study{42} included data that suggested some participants felt guilty using up what they perceived to be too much of their health care provider's time (in an intervention involving regular GP visits):

> *"I must admit I felt frequently embarrassed that I was taking up a lot of my GP's time." (No sample characteristics provided). {42}*

**Dietary elements and physical activities**

Although the majority of participants tended to describe valuing the flexibility and variety of the diet formats offered within programmes,{22,34,38,47} views were sometimes mixed with regard to diets, with a few wanting more prescriptive and structured eating plans than were offered:



For peer review only - http://bmjopen.bmj.com/site/about/guidelines.xhtml

> *"I think [having a set meal plan to follow] would have been to a certain extent easier at the beginning, but I don't think it would of actually adjusted my attitudes and thinking which it [POWeR+] has done (Male; 64 years; face-to-face support; high user)." (No sample characteristics provided).{34}*

The above quote illustrates that participants often discussed appreciating when programmes apparently emphasised changing attitudes towards food and eating over promoting a specific diet *per se*. However, sometimes participants did feel that their programme (or their primary care providers) tended to over emphasise diet rather than, for example, addressing issues around exercise, sleep or addiction problems.{37,45}

> *"…there was no support counselling-wise as to why I have the issues I have with food…" (Male).{37}*

Whilst many participants described the positive psychological and physical benefits they experienced from exercising, {17,22,45} others described struggling to engage in exercise. Some described disliking the perceived high intensity of the exercises (e.g. feeling uncomfortable with sweating, {22,26,27} whilst others discussed how their various physical or mental health comorbidities could prohibit them from full engagement in activities.{16,22,26,27,34,35,36,37,45}

> *"Exercise is the best [to lose weight] and I get all this physical therapy exercise and all of that just increases my pain, which reduces my desire to have any exercise." (No sample characteristics provided).{16}*

> *"I think for me, with my disability it was difficult to engage with some of the activities recommended." (No sample characteristics provided).{35}*

**Programme tools and BCTs designed to support behaviour change**

Participants suggested that many of the WMP's tools and techniques were helpful for them in terms of reflecting on their habits and behaviours and for helping them to positively change their attitudes. However, some participants described these tools as being somewhat intrusive and sometimes inflexible in nature. For example, some participants described disliking food logs and found food diaries/goal setting/daily self-weighing and the monitoring of exercise as excessive and



For peer review only - http://bmjopen.bmj.com/site/about/guidelines.xhtml

too confrontational.{22,34,44,45} Others felt that programme staff did not appropriately monitor and feedback on progress:{15}

> *"I mean no one ever looked at it [food diary]. No one ever asked for it. I just did all the work, like, for nothing because no one ever asked me for it." (No sample characteristics provided). {15}*

Others expressed frustration with the perceived inflexibility of tools designed to record behaviour and activities and to support behaviour change. For example, not being able to record life events and/or comorbidities that might help to explain lack of achievement regarding weight loss:{34,39}

> *"I thought that might be useful [to] have something [to] explain why things are going as they are going." (Female; 59 years, remote support; high user). {34}*

> *"I would want to tailor the messages [daily text messages] to the things that I was most struggling with." (No sample characteristics provided). {39}*

With regard to psychological support, two papers highlighted that some people wanted more counselling for non-direct weight issues, such as mental health, recognising that these additional problems had implications for weight management. {37,44} In contrast, although many participants discussed the various positive psychological changes they experienced which seemed to relate to the BCTs/counselling employed within some of the WMPs, others found personal development classes challenging and confrontational and questioned their appropriateness:{25}

> *"I cannot benefit from it [the personal development classes]. I will never open up in that room and talk among others." (Male). {25}*

**Findings from the synthesis – provider participants**

Ten of the included papers provided qualitative data from a range of WMP providers. {18,19,21,24,26,28,34,39,40,41} Seven of these papers were linked to one of three of the same interventions. Programme providers who provided qualitative data were described as primary care providers; {21,28} nurses; {34} GPs and consumer representatives;{41} GPs; {40,42} mental health care workers, dietitians, and nurses; {18,19} GPs, weight management advisors, practice nurses, {24} and key personnel working at a residential weight loss centre. {25}



For peer review only - http://bmjopen.bmj.com/site/about/guidelines.xhtml

***General impressions of being involved in WMPs***

With the exception of one study, in which some GPs (but not all) were reportedly less enthusiastic, {24} views about being involved in a WMP were generally very positive, with health professionals acknowledging that engagement was potentially very useful for them in terms of facilitating a conversation around weight loss with participants, and recognising that this can often be challenging in their everyday practices. {34,40,41,42}

However, the authors of one study{18} noted that discussions about weight tend to be embedded within the context of conversations about other health issues (rather than being discrete or stand-alone) and argued that this could act as a potential barrier with regards to the implementation of WMPs within primary care:

> *"I don't have patients that come to see me just for obesity or…just one thing…yes they're one of my diabetic patients but … we're talking about their cholesterol today or their blood pressure and their weight another day." (Nurse, no other sample characteristics provided).* {18}

***Motivating factors for participants'/provider engagement in WMPs***

One paper included some insights from the perspectives of programme providers about what apparently motivated prospective participants to take part in a WMP. {21} Health care providers involved in the delivery of the programmes described how they regarded participants' perceptions of their professional 'buy in' to the intervention study (i.e. endorsement) as important and influential regarding their decisions to take part. {21} One study (linked to two papers){21,28} also reported unusual success at enrolling men which programme providers attributed to their endorsing it as a 'medical' programme:

> *"I think that [our affiliation with a research institution] helped make it into a legitimate type of program that* [our patients] *would have confidence in, not just one of these wild watermelon diets or things like that." (Primary Care Provider, no other sample characteristics provided).{21}*

In terms of disincentives towards retention in such WMPs, some providers reported that some participants could have unrealistic expectations about weight loss, not fully understanding programme goals and commitment and wanting a "quick fix":



For peer review only - http://bmjopen.bmj.com/site/about/guidelines.xhtml

> *"What they wanted was a quick fix…They want to lose pounds very quickly. And it doesn't happen…"(GP, no other sample characteristics provided)* {24}

Only one study {24} provided data around apparent barriers and facilitators to health professionals' own engagement with a specific WMP. They described how clinicians' pre-conceived beliefs and attitudes towards integrating WMPs into primary care settings were important and they noted that engaged practices (as opposed to less engaged practices) were characterised by active GP participation and 'buy in.'

***The importance of the people within the programme setting (for fostering a sense of accountability)***

In keeping with some key findings from participants across the included papers, programme providers reflected on the importance of WMPs for creating a sense of accountability both for themselves as professionals in terms of increasing their responsiveness and sensitivity to their participants' weight management plan and needs and also of their continued engagement, motivation and success: {21,40}

> *"...I think it just made me be more sensitive...I've been kinda tryin' to dial it* [being tough on the patients*] down a little bit" (Primary Care Provider, no other sample characteristics provided) {21}*

Programme providers also recognised and reflected on what they regarded to be the importance of establishing and maintaining good relationships and of giving positive reinforcement and encouragement and being supportive of their weight loss efforts.{18,21,28,34}

***The types of interaction/support offered***

Several health care providers recognised that the intensity of interactions between programme staff and participants was important for motivating the latter to stay engaged and to sustain behaviour changes. {21,28} However, several provider participants raised concerns about the reality of this for their everyday clinical practice when time constraints were a real issue. {18,19,41} Other health care providers raised concerns around a lack of interdisciplinary working within clinic settings, which could inhibit their abilities to support weight loss, as well as lack of clarity with regard to professional role remits within teams:



For peer review only - http://bmjopen.bmj.com/site/about/guidelines.xhtml

*"I work with our RN all the time so on a daily basis we talk about things going back and forth but the others* [referring to dietitian and mental health workers] *I don't really see to be honest." (Nurse, no other sample characteristics provided). {19}*

Although providers in the above study{19} raised broad issues in their interviews relating to these barriers, they reflected positively on the study WMP in terms of facilitating interdisciplinary collaboration.

**Views about mode of support**

In terms of views about mode of support, health providers in one primary care study{21} argued that telephone-delivered weight counselling was the most convenient for participants. In contrast, providers in another study (one that involved a residential WMP) {25} argued that face-to-face group interaction was essential and particularly useful for participants with severe obesity who often experience social isolation. In another primary care study, {34} views regarding mode of delivery of support were more mixed. Whilst recognising the practicalities of remote forms of support, programme providers (in this case nurses) argued that face-to-face interactions worked best in terms of helping them connect more effectively and facilitated participant engagement and motivation. Some even stated that they did not regard remote support as support at all.

**Views about levels of provider engagement**

Health care providers in one study{21} stated that they played a fairly peripheral role in aspects of programme delivery and that sometimes this made it difficult for them to fully engage with their patient and to assess their progress. They suggested that individualised feedback from other professionals involved in programme delivery (e.g. in this case weight loss health coaches) would have been helpful. However, the study also reported that the majority of health care providers valued the fact that they played a limited role in the WMP, with time constraints and specific skill sets being raised as issues. Another study {34} raised related issues around level of provider engagement with aspects of the WMP. In this case, nurses discussed the perceived disadvantage of not being able to view the information provided to participants on the study website. Some felt that viewing this information would have allowed them to understand more fully, what participants were referring to in consultations. In one study,{41} GPs commented on and seemed to value the relatively 'loose' nature of the intervention design (in this case a weight management toolkit) as they considered it offered scope to enable them to tailor it to the individual and their community.



For peer review only - http://bmjopen.bmj.com/site/about/guidelines.xhtml

Similarly, nurses in another study {34} expressed frustration around the lack of flexibility of their intervention, both in terms of how they were supposed to behave (i.e. by not being directive) and in terms of the scope within the website to document individual issues. This was also a concern raised by the participants themselves. Although, providers in these two studies {34,41} apparently appreciated interventions that were more flexible in nature (and therefore could be tailored more appropriately to individual care). Personnel in a residential WMP{25} specifically designed for people with severe obesity seemed to value having a very strict programme structure (in this case participants had to attend morning meetings, group activities, and eat six meals a day at fixed times). The general feeling amongst staff was that instilling this strictness on participants would facilitate behaviours that they would then seek to maintain at home.

**Views about intervention content**

Whilst some, (but not all), participants in one study{25} found personal development classes challenging and confrontational, providers in the same study consistently argued that personal development (i.e. focussing on personal factors such as self-knowledge and self-acceptance) was essential and crucially important for maintaining lifestyle changes longer term:

> *"It is important that they become aware of what in their life makes a difference in being obese or not." (Personnel, no other sample characteristics provided).{25}*

## Discussion

*Principal findings*

This review synthesised findings from qualitative data relating to the views of adults with BMI ≥35kg/m$^2$ (and/or their health care providers) about engaging with WMPs. In summary, although there was variation expressed in views about the acceptability of various programme components (indicating the inappropriateness of a 'one size fits all' approach), there were, nevertheless, recurring themes around what both participant and programme providers described valuing and enjoying. Some of these key findings resonate with previous qualitative research with people with less severe obesity. {7,48}.

Participants in our review described being attracted to WMPs that were perceived to be novel or exciting in some key way (e.g. being different to programmes that they had tried previously), as well as perceived to have been endorsed by their health care providers (a view supported by



For peer review only - http://bmjopen.bmj.com/site/about/guidelines.xhtml

programme providers themselves). The sense of belonging to a group of people who shared similar issues relating to weight and food, and who had similar physiques and personalities, was described as being particularly important to many participants and seemed to foster a strong group identity and related ‘accountability’, which seemed to help with motivation and continuing engagement.

Good relationships with programme providers were described as being highly valued, with ongoing encouragement and monitoring apparently important for facilitating motivation and behaviour change (a view also endorsed by the programme providers themselves). Group based programme activities were apparently enjoyed by many participants along with fairly intensive support from programme providers. This observation is supported in previous qualitative research with people with less severe obesity{7,48}. However, in our review, although described by both participants and programme providers as being important for supporting engagement and positive behaviour changes, concerns were raised about the availability of continuing support post intervention, and similarly by providers who questioned the practicalities and logistics of integrating such intense support into their everyday clinical practices once the studies were completed.

Overall, both participants and programme providers valued having choice and flexibility. For example, participants welcomed flexibility around diet choices, flexibility around when face-to-face counselling sessions, and also welcomed personalised interventions. Similarly, some programme providers found the perceived lack of flexibility with various intervention components frustrating and prohibitive in terms of supporting individualised care.

Those participants who described engaging in group discussions/therapy sessions (with other participants and/or providers) and those who discussed engaging in exercises were mainly positive about their perceived benefits. For example, where it was discussed, participants very much valued the psychological input integrated into many interventions. This is a view supported in a study of user experiences of both Tier 2 and Tier 3 weight management services in England{48}. However, it is worth noting that our review also highlighted that some participants did describe struggling with these aspects, with some describing them as particularly challenging. Some participants described difficulties with the various physical activities (because of a range of physical comorbidities) and not everyone enjoyed group interaction and discussions with others (sometimes apparently because they suffered from various mental health comorbidities).

*Practice Implications*



For peer review only - http://bmjopen.bmj.com/site/about/guidelines.xhtml

For intervention developers, it was clear from our review that social interaction activities tended to be valued. It was also apparent that ongoing encouragement and monitoring by programme providers was viewed as important for facilitating motivation and behaviour change. The waning intensity of programme activities and/or programme cessation could cause problems for maintaining behaviour change patterns if group interaction and support was an integral component. Perhaps there is a need for WMPs to help consumers to establish support post intervention.

People with severe obesity might be especially vulnerable to both physical and mental comorbidities, which could inhibit engagement with certain intervention components (e.g. group based interaction; physical activities). For intervention developers, this is worthy of note. This could inhibit their engagement with much fitter peers with fewer weight-related issues, or restrict their ability to undertake certain intervention components – an observation that is less apparent in research with people with less severe obesity {7}. Perhaps WMPs could consider including a choice of interaction styles/mix of physical activities to accommodate this.

*Strengths and limitations*

To our knowledge, this is the first synthesis of key findings from qualitative studies exploring participants' perspectives of WMPs for adults with severe obesity. Our synthesis has highlighted a range of important factors that have the potential to facilitate engagement with WMPs for this group.

We were interested in ascertaining the views of participants with severe obesity (people with BMI $\geq$35kg/m$^2$). Therefore, our inclusion criteria were that papers needed to state that participants in their respective studies (i.e. either in their qualitative evaluations or the intervention studies to which their qualitative evaluations were linked) had a mean BMI $\geq$35kg/m$^2$. Of those papers that only considered programme providers' views, these had to be linked to intervention studies where we could establish that included participants had a mean BMI $\geq$35kg/m$^2$. Only two papers stated that their respective WMPs were designed *specifically* for people with BMI $\geq$35kg/m$^2$. {22,40} Thus, across the papers, some people with BMI $<$35kg/m$^2$ would have been included. Quotes from participants were not linked to specific detail regarding BMI status, and so we cannot be certain that findings reflect exclusively the views of those with severe obesity.



For peer review only - http://bmjopen.bmj.com/site/about/guidelines.xhtml

Only nine papers linked participant quotes to sex; {22,25,27,29,33,34,36,37,38} only one to age status;{34} and none to socioeconomic/demographic characteristics, making it hard for us to consider whether any issues raised were particularly sensitive or pertinent to these aspects.

We know from a recent review of Tier 3 weight management interventions for adults with severe obesity that drop-out rates are very high (43-63%). {49} Only four of our included papers stated that some of the participants in their qualitative evaluations had been ‘low users’, ‘quitters’ or ‘drop-outs’{15,22,23,34} and only one of these papers linked quotes directly to intervention usage status. {34} Although our findings highlighted a range of views with regard to the usefulness or otherwise of various intervention components, it is worth noting that participant sample characteristics within the included papers are skewed towards those who had chosen to engage and who had completed the various intervention activities.

Applying quality criteria to qualitative research remains a contentious issue and there is no consensus regarding whether and how this should be done {50,51}. Whilst authors of some qualitative evidence syntheses have chosen to exclude what they deem to be poor quality papers, we made the decision not to exclude any of the identified papers. We included 33 papers that each reported some qualitative data that met our inclusion criteria and addressed our key research questions. Although all included qualitative data, in terms of ‘quality,’ some were deemed richer than others in terms of data and insights - some ranged from being exclusively qualitative studies providing rich data in our areas of interest, through to studies that were actually primarily quantitative with responses to open-ended survey questions. The five studies providing qualitative data in the form of responses to open-ended survey questions within structured questionnaires{20,30,35,44,47} were deemed less useful in terms of presenting only very limited qualitative data and insights. Despite this variation in the overall level of quality, we felt it was more important to retain any relevant findings rather than disregard based on study quality. In doing so, we would argue that all 33 papers contributed useful elements to the collective whole and enabled us to develop our understanding of the issues of importance to people with BMI $\geq$35kg/m$^2$. We cannot exclude the possibility that unpublished service evaluations from within the NHS, that we failed to locate, might have been sources of rich data.

*Implications for research*



For peer review only - http://bmjopen.bmj.com/site/about/guidelines.xhtml

No papers included in our review provided qualitative data from those who had been invited to join a WMP but who had declined to take part, and only four papers reported including participants who had not fully engaged with all programme activities to varying degrees. Clearly the views of those who do not engage are important and should be a focus of future research. Therefore, in terms of pointers for effective interventions, it is worth acknowledging that key findings will be skewed towards those who had chosen to engage and who had completed the various intervention activities. In terms of implications for research, it is clear that the qualitative research literature focusing specifically on lifestyle WMPs for people with very high BMIs is limited, particularly for people who are low-users or do not wish to engage with such services.

*Conclusions*

WMPs that are perceived to be novel or exciting and WMPs that are perceived to be endorsed by health care providers tend to be valued by participants. The sense of belonging to a group of people who share similar issues and characteristics seems particularly important, helping to foster a strong group identity and related 'accountability', which aids motivation and continuing engagement. In person group based programme activities tend to be valued (over more remote forms of support), along with fairly intensive support from programme providers. However, intervention developers should bear in mind that people with severe obesity might be especially vulnerable to both physical and mental co-morbidities that could inhibit engagement with certain intervention components.

Supporting Information

S1 Appendix: Search strategies

S1 ENTREQ Checklist

S1 Table: Characteristics of included studies

S1 Figure

S1 Conceptual Diagram

**Acknowledgements**

*REBALANCE Project management team Elisabet Jacobsen[1], Dwayne Boyers[2], David Cooper[1], Lise Retat[3], Paul Aveyard[4], Fiona Stewart[1], Graeme MacLennan[1], Laura Webber[3], Emily Corbould[3], Benshuai Xu[3], Abbygail Jaccard[3], Bonnie Boyle[1], Eilidh Duncan[1], Michal Shimonovich[1], Cynthia Fraser[1], Lara Kemp[1].



For peer review only - http://bmjopen.bmj.com/site/about/guidelines.xhtml

REBALANCE Advisory Group for all their advice and support during this project: Margaret Watson, Lorna Van Lierop, Richard Clarke, Jennifer Logue, Laura Stewart, Richard Welbourn, Jamie Blackshaw, Su Sethi.

[1]Health Economics Research Unit, University of Aberdeen, Aberdeen, UK

[2]Health Services Research Unit, University of Aberdeen, Aberdeen, UK

[3]UK Health Forum, Fleetbank House, Salisbury Square, London, UK

[4]Nuffield Department of Primary Care Health Sciences, Oxford University, Oxford, UK

**Contributors**

AA and ZCS conceived the study idea for the qualitative synthesis. ZCS and MAM screened all titles and abstracts. ZCS and MAM conducted the data analysis and ZCS wrote the initial and subsequent manuscript drafts. AA, ZCS, MAM, CR, MdB contributed critically to discussions about interpretation of data and revisions of manuscript drafts. AA, ZCS, MAM, CR, MdB approved the final version.

**Funding**

The project was funded by the NIHR Health Technology Assessment Programme (Project number: 15/09/04). See the HTA Programme website for further project information.

The views and opinions expressed therein are those of the authors and do not necessarily reflect those of the Department of Health, or the funders that provide institutional support for the authors of this report.

**Data sharing statement**

This is a review of published studies which are available to access through the relevant journals.

**Competing interests statement**

There are no competing interests for any author.

1. National Institute for Health and Care Excellence. Obesity: identification, assessment and management of overweight and obesity in children, young people and adults. (Partial update of CG43). 2014. http://www.nice.org.uk/guidance/cg189
2. Royal College of Surgeons. Commissioning guide: weight assessment and management clinics (tier 3), 2014. https://www.rcseng.ac.uk/healthcare-bodies/docs/weight-assessment-and-management-tier-3-services.
3. The Look AHEAD Research Group. Cardiovascular effects of intensive lifestyle intervention in type 2 diabetes. N Eng J Med 2013;369:145-54.
4. Scottish Intercollegiate Guidelines Network. Management of obesity. Edinburgh; SIGN: 2010. http://www.sign.ac.uk/guidelines/fulltext/115/index.html
5. National Institute for Health and Care Excellence. Managing overweight and obesity in



For peer review only - http://bmjopen.bmj.com/site/about/guidelines.xhtml

adults—lifestyle weight management services. (Public Health Guideline PH53).2014

6. Avenell A, Robertson C, Skea ZC, Jacobsen E, Boyers D, Cooper D et al. Bariatric surgery, lifestyle interventions and orlistat for severe obesity: The REBALANCE mixed-methods systematic review and economic evaluation. Health Technology Assessment, vol 22,no.68.
7. Sutcliffe K, Richardson M, Rees R, Burchett H, Melendez-Torres GJ, Stansfield C, Thomas J (2016) What are the critical features of successful Tier 2 weight management programmes for adults? A systematic review to identify the programme characteristics, and combinations of characteristics, that are associated with successful weight loss. London: EPPI-Centre, Social Science Research Unit, UCL Institute of Education, University College London.
8. Robertson C, Archibald D, Avenell A, Douglas F, Hoddinott P, van Teijlingen E, et al. Systematic reviews of and integrated report on the quantitative, qualitative and economic evidence base for the management of obesity in men. Health Technol Assess 2014;13:35.
9. Barnett-Page E, Thomas J. Methods for the synthesis of qualitative research: A critical review. *BMC Med Res Method* 2009;**9:**59**.**
10. Spencer L, Ritchie J, Lewis J, Dillon L. Quality in qualitative evaluation: a framework for assessing research evidence. London: Cabinet Office; 2003
11. Pawson R. Evidence-based policy: a realist perspective. London: Sage; 2006
12. Thomas J, Harden A. Methods for the thematic synthesis of qualitative research in systematic reviews. *BMC Med Res Method* 2008;**8:**45.
13. Harden A, Brunton G, Fletcher A, Oakley A. Teenage pregnancy and social disadvantage: systematic review integrating controlled trials and qualitative studies. *BMJ* 2009;**339:**b4254.
14. Toye F, Seers K, Allcock N, Briggs M, Carr E, Andrews J, et al. 'Trying to pin down jelly' - exploring intuitive processes in quality assessment for meta-ethnography. *BMC Med Res Method* 2013;**13:**46.
15. Abildso C, Zizzi S, Gilleland D, Thomas J, Bonner D. A mixed methods evaluation of a 12-week insurance-sponsored weight management program incorporating cognitive-behavioral counseling. *J Mix Methods Res* 2010;**4:**278-94.
16. Amy Janke E, Kozak AT. "The more pain I have, the more I want to eat": obesity in the context of chronic pain. *Obesity* 2012;**20:**2027-34.
17. Aschbrenner KA, Naslund JA, Bartels SJ. A Mixed Methods Study of Peer-to-Peer Support in a Group-Based Lifestyle Intervention for Adults With Serious Mental Illness. *Psychiatr Rehabil J* 2016;**39:**328-34.
18. Asselin J, Osunlana AM, Ogunleye AA, Sharma AM, Campbell-Scherer D. Missing an opportunity: the embedded nature of weight management in primary care. *Clin Obes* 2015;**5:**325-32.
19. Asselin J, Osunlana AM, Ogunleye AA, Sharma AM, Campbell-Scherer D. Challenges in interdisciplinary weight management in primary care: lessons learned from the 5As Team study. *Clin Obes* 2016;**6:**124-32.
20. Barham K, West S, Trief P, Morrow C, Wade M, Weinstock RS. Diabetes prevention and control in the workplace: a pilot project for county employees. *JPHMP* 2011;**17:**233-41.



For peer review only - http://bmjopen.bmj.com/site/about/guidelines.xhtml

21. Bennett WL, Gudzune KA, Appel LJ, Clark JM. Insights from the POWER practice-based weight loss trial: a focus group study on the PCP's role in weight management. *J Gen Intern Med* 2014;**29:**50-8.
22. Borkoles E, Carroll S, Clough P, Polman RCJ. Effect of a non-dieting lifestyle randomised control trial on psychological well-being and weight management in morbidly obese pre-menopausal women. *Maturitas* 2016;**83:**51-8.
23. Bradbury K, Dennison L, Little P, Yardley L. Using mixed methods to develop and evaluate an online weight management intervention. *Br J Health Psychol* 2015;**20:**45-55.
24. Counterweight Project T, McQuigg M, Brown JE, Broom JI, Laws RA, Reckless JP, et al. Engaging patients, clinicians and health funders in weight management: the Counterweight Programme. *Fam Pract* 2008;**25 Suppl 1:**i79-86.
25. Dahl U, Rise MB, Kulseng B, Steinsbekk A. Personnel and participant experiences of a residential weight-loss program. A qualitative study. *PLoS ONE* 2014;**9:**e100226.
26. Danielsen KK, Sundgot-Borgen J, Rugseth G. Severe Obesity and the Ambivalence of Attending Physical Activity: Exploring Lived Experiences. *Qual Health Res* 2016;**26:**685-96.
27. Groven KS, Engelsrud G. Dilemmas in the process of weight reduction: Exploring how women experience training as a means of losing weight. *Int J Qual Stud Health Well-being* 2010;**5**.
28. Gudzune KA, Clark JM, Appel LJ, Bennett WL. Primary care providers' communication with patients during weight counseling: a focus group study. *Patient Educ Couns* 2012;**89:**152-7.
29. Hunt K, Gray CM, Maclean A, Smillie S, Bunn C, Wyke S. Do weight management programmes delivered at professional football clubs attract and engage high risk men? A mixed-methods study. *BMC Public Health* 2014;**14:**50.
30. Jackson C, Coe A, Cheater FM, Wroe S. Specialist health visitor-led weight management intervention in primary care: exploratory evaluation. *J Adv Nurs* 2007;**58:**23-34.
31. Jennings A, Hughes CA, Kumaravel B, Bachmann MO, Steel N, Capehorn M, et al. Evaluation of a multidisciplinary Tier 3 weight management service for adults with morbid obesity, or obesity and comorbidities, based in primary care. *Clin Obes* 2014;**4:**254-66.
32. Jimenez-Lopez JL, Maldonado-Guzman ME, Flores-Perez Pasten L, Deciga-Garcia E. [Reasons for losing weight: Why have programed support?]. *Rev Med Inst Mex Seguro Soc* 2012;**50:**407-12.
33. Kidd LI, Graor CH, Murrock CJ. A mindful eating group intervention for obese women: a mixed methods feasibility study. *Arch Psychiatric Nurs* 2013;**27:**211-8.
34. Little P, Stuart B, Richard Hobbs FD, Kelly J, Smith ER, Bradbury KJ, et al. Randomised controlled trial and economic analysis of an internet-based weight management programme: POWeR+ (Positive Online Weight Reduction). *Health Technol Assess* 2017;**21:**1-61.
35. McRobbie H, Hajek P, Peerbux S, Kahan BC, Eldridge S, Trepel D, et al. Tackling obesity in areas of high social deprivation: clinical effectiveness and cost-effectiveness of a task-based weight management group programme - a randomised controlled trial and economic evaluation. *Health Technol Assess* 2016;**20:**79.
36. Owen-Smith A, Donovan J, Coast J. "Vicious circles": the development of morbid obesity. *Qual Health Res* 2014;**24:**1212-20.
37. Owen-Smith A, Donovan J, Coast J. Experiences of accessing obesity surgery on the NHS: a qualitative study. *J Public Health* 2016;**31:**31.
38. Pera PI, Ferrer MCO, Juarez MN, Juarez EN, Soler LM, Matheu CL, et al. Obesity, knee osteoarthritis, and polypathology: Factors favoring weight loss in older people. *Patient Prefer Adher* 2016;**10:**957-65.
39. Shaw RJ, Bosworth HB, Silva SS, Lipkus IM, Davis LL, Sha RS, et al. Mobile health messages help sustain recent weight loss. *Am J Med* 2013;**126:**1002-9.

For peer review only



For peer review only - http://bmjopen.bmj.com/site/about/guidelines.xhtml

40. Sturgiss E, Haesler E, Elmitt N, van Weel C, Douglas K. Increasing general practitioners' confidence and self-efficacy in managing obesity: a mixed methods study. *BMJ Open* 2017;**7:**e014314.
41. Sturgiss EA, Douglas K. A collaborative process for developing a weight management toolkit for general practitioners in Australia-an intervention development study using the Knowledge To Action framework. *Pilot Feasib Stud* 2016;**2:**20.
42. Sturgiss EA, Elmitt N, Haesler E, van Weelc C, Douglas K. Feasibility and acceptability of a physician delivered weight management programme. *Fam Pract* 2017;**34:**43-8.
43. Turner D, Haboubi N. Qualitative and Quantitative Outcomes of a 1:1 Multidisciplinary Weight Management Clinic. *Healthcare* 2015;**3:**429-51.
44. VanWormer JJ, Martinez AM, Cosentino D, Pronk NP. Satisfaction with a weight loss program: what matters? *Am J Health Promot* 2010;**24:**238-45.
45. Yarborough BJ, Stumbo SP, Yarborough MT, Young TJ, Green CA. Improving lifestyle interventions for people with serious mental illnesses: Qualitative results from the STRIDE study. *Psychiatr Rehabil J* 2016;**39:**33-41.
46. Young AS, Cohen AN, Goldberg R, Hellemann G, Kreyenbuhl J, Niv N, et al. Improving Weight in People with Serious Mental Illness: The Effectiveness of Computerized Services with Peer Coaches. *J Gen Intern Med* 2017;**32:**48-55.
47. Zizzi S, Kadushin P, Michel J, Abildso C. Client experiences with dietary, exercise, and behavioral services in a community-based weight management program. *Health Promot Pract* 2016;**17:**98-106.
48. Guzelegun N, Sissoko F, Jeong Ko U, Harrington J, Lanford K, Coulton V, et al. Qualitative opportunities into user experiences of tier 2 and tier 3 weight management services: Public Health England; 2017. https://www.gov.uk/government/publications/weight-management-services-insights-into-user-experiences [Accessed April 2018]
49. Brown 2017 Brown TJ, O'Malley C, Blackshaw J, Coulton V, Tedstone A, Summerbell C, et al. Exploring the evidence base for Tier 3 weight management interventions for adults: a systematic review. *Clin Obes* 2017;**7:**260-72.
50. Campbell R, Pound P, Morgan M, Daker-White G, Britten N, Pill R, Yardley L, Pope C, Donovan J: Evaluating meta-ethnography: systematic analysis and synthesis of qualitative research. Health Technol Assess 2011, 15(43):1–164. 3.
51. Dixon-Woods M, Sutton A, Shaw R, Miller T, Smith J, Young B, Bonas S, Booth A, Jones D: Appraising qualitative research for inclusion in systematic reviews: a quantitative and qualitative comparison of three methods. J Health Serv Res Policy 2007, 12(1):42–47



For peer review only - http://bmjopen.bmj.com/site/about/guidelines.xhtml

**S1 Table Characteristics of the included qualitative studies**

| Study | Aim (as described within the papers) | Condition of Focus | Participants Characteristics | Details of intervention | Qualitative data collection methods |
|---|---|---|---|---|---|
| *First Author*: Bennett<br>*Year:* 2014<br>*Category*: A<br>*Country*: USA | To understand primary care providers' (PCPs) perspectives about their role in the intervention and in their patients' weight loss, thereby providing insights to inform best practices in developing practice-based weight management programmes. | Patients with obesity in their usual care practices. | *Role:* Provider<br>*Number providers interviewed*: 26 PCPs<br>*Providers' characteristics*: 15 female, 11 male, 24 physicians, 2 nurse practitioners, and 20 had internal medicine training. The mean time in practice was 16 years (SD ± 11.7), and mean number of patients in the trial was 11.1 (SD ± 6.8)<br>*Socioeconomic and demographic characteristics*: 15 White, 6 Asian/Pacific Islander, 3 Black, 2 Other | The Practice-based Opportunities for Weight Reduction (POWER) was a 24 month trial that had two intervention groups (by phone and face-to-face) in which weight-loss health coaches (not PCPs) provided education and positive reinforcement. Participants in both intervention arms had access to the same online educational modules, self-monitoring tools and received both automated and individualized e-mails. Participants in the control arm met with a weight loss health coach at the time of randomization and, if desired, after the final data collection visit. They also received brochures along with a list of recommended weight loss websites. | Focus groups |
| *First Author*: Bradbury<br>*Year:* 2015<br>*Category*: A | To explore helpful (and unhelpful) aspects of coaching; | Participants with obesity. | *Role:* Participant<br>*Number of participants*: 58. | Positive Online Weight Reduction (POWeR) is an e-health intervention designed to produce sustainable weight | Interviews |

| | | | | | |
|---|---|---|---|---|---|
| *Country*: UK | the experiences of POWeR and the accompanying coaching, including what aspects people found most helpful, unhelpful, appealing or unappealing, and what factors seemed to influence whether participants continued to follow POWeR. | | Planning and development stages: 16 participants;<br>Feasibility stage: 23 participants;<br>Community trial 19 participants.<br>*Participants' characteristics*: From the community trial: age range 34-68, Participants were sampled from both the coaching arm (10 female, four male) and Web only arm (four female, one male) and varied in their usage of POWeR.<br>*Socioeconomic and demographic characteristics*: NR<br>*Comorbidities*: NR | management. POWeR consisted of 12 sessions which taught users self-regulation skills in order for them to become their own personal health trainer. Patients were randomized to either usual care, the POWeR website, POWeR accompanied by basic nurse support, or POWeR with regular nurse support. The nurse support was mainly delivered face to face, although telephone and email support could also be provided. | |
| *First Author*: Gudzune<br>*Year:* 2012<br>*Category*: A<br>*Country*: USA | To explore PCPs' usual practices as part of weight counselling to identify how PCPs communicate with their patients about weight loss. | Patients with obesity in their usual care practices | See *Bennett 2014* | See *Bennett 2014* | Focus groups |
| *First Author*: Hunt<br>*Year:* 2014<br>*Category*: A<br>*Country*: UK | To report the characteristics of men participating in a randomised | Men with obesity (BMI > 28kg/m$^2$), age 35–65 at high | *Role:* Participant<br>*Number of participants*: 63 men (who had attended at least six FFIT sessions of the programme). | Football Fans in Training (FFIT) is a men-only, evidence-based, 12-session, weight management and physical activity group programme with subsequent | Focus groups |

For peer review only

| | | | | | |
|---|---|---|---|---|---|
| | controlled trial of a weight management programme designed specifically to attract men, and, secondly, their accounts of why they decided to participate in the programme. | risk of ill-health due to obesity | *Participants characteristics*: No specific data for qualitative analysed participants<br>*Socioeconomic and demographic characteristics*: NR<br>*Comorbidities reported*: NR | minimal-contact weight loss maintenance support delivered free of charge at Scotland's top professional football clubs by community coaches trained in diet, nutrition, physical activity and behaviour change techniques to a standard programme delivery protocol. | |
| *First Author*: Little<br>*Year:* 2017<br>*Category*: A<br>*Country*: UK | To explore patients' expectations of POWeR+, experiences of the POWeR+ programme, experiences of using the POWeR+ website and experiences of nurse support. | Participants with obesity (BMI $\geq$30kg/m$^2$, or $\geq$28kg/m$^2$ with comorbidities) from general practice | *Role:* Participant and Provider<br>*Number of providers*: 13 nurses (HCPs who supported POWeR+ were included in qualitative evaluation)<br>*Number of participants:* 31 POWeR+ programme users. 14 remote support (3 low users/11 high users) and 17 face-to-face support patients (2 low users/15 high users).<br>*Participants' characteristics*: 15 female, 16 male, mean age 61 years (range 45-88 years).<br>*Socioeconomic and demographic characteristics*: No specific data for qualitative analysed participants.<br>*Comorbidities reported*: No specific | This is a 24-session web-based weight management intervention consisting of a series of 24 brief maintenance-oriented sessions for up to 6 months and links to encourage patients to continue to use the website to track their weight at least fortnightly until they have formed healthy eating habits that sustain weight management. | Interviews |

For peer review only

| | | | | | |
|---|---|---|---|---|---|
| | | | data for qualitative analysed participants. | | |
| *First Author*: McRobbie<br>*Year:* 2016<br>*Category*: A<br>*Country*: UK | To explore the many components of the WAP. By providing a summary of participant feedback on the overall helpfulness of the programme. | Adults (aged ≥ 18 years) with obesity (BMI of ≥ 30 kg/m$^2$ or a BMI of ≥ 28 kg/m$^2$ plus comorbidities) who wanted to lose weight | *Role:* Participant<br>*Number of participants:* 177. Participants who reported helpfulness of the programme at 12-months follow up; 48 in the nurse arm and 129 in the WAP arm. People who dropped out of treatment were called; only 19 provided a reason for dropping out.<br>*Participants' characteristics*: Not reported<br>*Socioeconomic and demographic characteristics:* Not reported.<br>*Comorbidities:* Not reported | The WAP is a multicomponent programme that includes a range of concrete and verifiable tasks agreed individually with each participant and also includes monthly 'maintenance' sessions that targeted to improve participant motivation, allowing participants to discuss the challenges they have faced since the last session, and to anticipate challenges of the month ahead. | Anonymous feedback questionnaire |
| *First Author*: Yarborough<br>*Year:* 2016<br>*Category*: A<br>*Country*: USA | To assess lifestyle change barriers and facilitators across the first 18 months of study participation and to identify modifiable factors associated with making and maintaining healthy | Adults (aged ≥ 18 years) with obesity (BMI ≥27kg/m$^2$) taking antipsychotic medications (stable on antipsychotic medications for at least 30 days) | *Role:* Participant<br>*Number of participants*: 84. Participants in the control arm were interviewed once; 17 intervention participants were interviewed more than once to ensure that all cohorts were represented in each interview wave.<br>*Participants' characteristics*: Mean age 48.1 (SD ± 10.1), 30 male, 54 | This was a 24-month study of the STRIDE comprehensive weight loss and lifestyle-change intervention that consisted of 24 weekly meetings that targeted readiness to change; included interactive, participant-centred delivery of lifestyle education information along with a 20-min walk; encouraged skills practice, self-monitoring and feedback; and facilitated group interactions and | Interviews |

For peer review only

| | | | | | |
|---|---|---|---|---|---|
| | lifestyle changes in order to inform clinicians and improve the development of future interventions for individuals with serious mental illnesses. | | female. 18 were members of ethnic or racial minorities.<br>*Socioeconomic and demographic characteristics*: 34 married or living with partner, 27 had an income of $30,000 or higher, 18 were college graduate or higher, 28 were retired, unemployed, student, homemaker or temporarily laid off.<br>*Comorbidities*: 34 Schizophrenia, 17 bipolar disorder, 31 affective psychoses, 2 PTSD | support. Intervention participants could consult with interventionists by telephone as needed. | |
| *First Author*: Abildso<br>*Year:* 2010<br>*Category*: B<br>*Country*: USA | To examine physical and psychosocial differences at baseline between completers of and dropouts from a 12-week weight management program; to assess the physical, behavioural, and psychosocial impact on program completers; to | Adults with obesity (BMI ≥ 30kg/m$^2$ alone or a BMI of 25 to 29.9kg/m$^2$ with comorbidities) | *Role:* Participant<br>*Number of participants*: 11<br>*Participants characteristics*: Mean age 46.2 (SD ± 8.5), 8 female, 3 male. Seven were successful program completers (three high weight losers, four moderate weight losers), and four were program dropouts or completers with low weight loss).<br>*Socioeconomic and demographic characteristics*: 7 married, number of children 1.5 (SD ± 1.1)<br>*Comorbidities*: Not reported | Weight loss is encouraged in the weight management program (WMP) through increasing physical activity and decreasing caloric intake. For a $45 monthly co-payment, the WMP benefit during Phase 1 (12 weeks) included assessment and follow-up meetings with an exercise physiologist and registered dietitian, monthly personal training sessions, and periodic phone calls from the insurance agency to track progress. | Interviews |

For peer review only

| | | | | | |
|---|---|---|---|---|---|
| | compare the psychosocial changes of high and moderate weight losers; and to qualitatively explore factors associated with program adherence and weight loss. | | | | |
| *First Author*: Aschbrenner<br>*Year:* 2016<br>*Category*: B<br>*Country*: USA | To explore participants' perceptions and experiences with peer interactions during the lifestyle intervention. | Obese (BMI ≥ 30kg/m$^2$) adults (aged 21 or older) with serious mental illness (diagnosis of schizophrenia, schizoaffective disorder, major depressive disorder, or bipolar disorder) on stable pharmacological treatment | *Role:* Participant<br>*Number of participants*: 17<br>*Participants' characteristics*: No specific data for qualitative analysed participants<br>*Socioeconomic and demographic characteristics*: Not reported<br>*Comorbidities:* Not reported | A 24-week group-based lifestyle intervention that consisted of once weekly 1-hr group weight management sessions facilitated by a psychologist and a public health professional; twice weekly (optional) 1-hr group exercise sessions led by a certified fitness trainer; and mobile technology and use of social media to increase motivation and facilitate self-monitoring and peer-to-peer support outside of in person group treatment or exercise sessions. | Focus groups |

For peer review only

| | | | | | |
|---|---|---|---|---|---|
| *First Author*: Asselin<br>*Year:* 2015<br>*Category*: B<br>*Country*: Canada | To explore how primary care providers incorporate weight management in their practice. | Obesity prevention and weight management at interdisciplinary primary care environment | *Role:* Provider<br>*Number of providers interviewed:* 29<br>*Providers' characteristics*: 7 mental healthcare workers, 7 registered dietitians, 15 registered nurses or nurse practitioners.<br>*Socioeconomic and demographic characteristics:* NR | The 5 As Team (5AsT) study was designed to create, implement and evaluate a flexible intervention to improve the quality and quantity of weight management visits in primary care. 5AsT is a randomized controlled trial on the implementation of a 6-month 5AsT intervention designed to operationalize the 5As of obesity management in primary care. | Interviews and field notes of intervention sessions |
| *First Author*: Asselin<br>*Year:* 2016<br>*Category*: B<br>*Country*: Canada | To describe the intervention, provide continual intervention monitoring and to identify contextual factors that could influence the primary outcome measure. | See *Asselin 2015* | See *Asselin 2015* | See *Asselin 2015* | See *Asselin 2015* |
| *First Author*: Barham<br>*Year:* 2011<br>*Category*: B<br>*Country*: USA | To improve nutrition and physical activity of county employees and promote weight loss (*There was no* | Adults at highest risk for the development of diabetes or who already have been | *Role:* Participant<br>*Number of participants*: Unclear how many of 45 programme participants provided written responses on the end of study programme evaluations. | There were 2 waves of enrolment and 4 intervention groups (up to 12 participants/ group). The intervention was a 3-month program (12 one hour weekly midday group sessions) that targeted healthy diet, physical activity, | Written responses to end of programme participant evaluations |

For peer review only

| | | | | | |
|---|---|---|---|---|---|
| | *qualitative aim stated).* | diagnosed with type 2 diabetes | *Participants characteristics*: No specific data for those who provided written responses<br>*Socioeconomic and demographic characteristics*: Not reported<br>*Comorbidities reported*: Not reported | and stress reduction, followed by a monthly maintenance program with the groups choosing topics that they considered of greatest benefit. Most of the sessions were led by a nurse educator, but individual sessions were also conducted by a dietitian, psychologist, and physical therapist all employees of Upstate Medical University, Syracuse, NY. | |
| *First Author*: Borkoles<br>*Year:* 2016<br>*Category*: B<br>*Country*: UK | To examine the effects of a non-dieting lifestyle intervention approach for women with morbid obesity designed in the framework of the self-determination theory and Health at Every Size on weight maintenance and psychological functioning. | Pre-menopausal females with morbid obesity (BMI ≥30kg/m$^2$) older than 18 years of age free of obesity-related diseases and fit for exercise | *Role:* Participant<br>*Number of participants*: 62 (62 interviews at baseline with 36 follow-up interviews, including 12 drop-outs).<br>*Participants' characteristics*: Pre-menopausal women predominantly white Caucasian (97%), with a mean age of 40.2 years<br>*Socioeconomic and demographic characteristics*: most were from the lower SES background, 21% had a degree and 57% left school at 16, 66.1% worked full time and 11% worked part-time, in mainly manual | The WHEEL (Weight, Healthy Eating and Exercise in Leeds) study was a delayed-start, 12 weeks of intensive intervention and 40-week maintenance phase RCT comprising of community-based supervised exercise, lifestyle physical activity and psycho-educational classes on healthy eating and weight management. | Interviews |

For peer review only

| | | | | | |
|---|---|---|---|---|---|
| | | | (29%) and administrative jobs (46.8%)<br>*Comorbidities*: 50% met the International Diabetes Federation metabolic syndrome criteria, 42% reported to have depression often or very often, and 36% used medication related to psychological problems | | |
| *First Author*: Dahl<br>*Year:* 2014<br>*Category*: B<br>*Country*: Norway | To describe how personnel argued for and perceived a residential weight-loss program, to investigate how the participants experienced the program, and to contrast these perspectives. | Adults (between 18 and 60 years old) with obesity (BMI > 40kg/$m^2$ or >35kg/$m^2$ including comorbidities)<br>*Providers*:<br>The personnel were recruited among the staff at the centre | *Role:* Participant and Provider<br>*Number of participants*: 10<br>*Participants' characteristics*: 10 Norwegian participants took part in interviews (8 in focus groups and 2 individually). The age and weight range for these 10 persons were the same as for the total sample (n=30). Age between 22 and 56 years old, their BMI was between 40 and 63, and the group's mean body weight was 144kg<br>*Socioeconomic and demographic characteristics*: NR<br>*Comorbidities:* NR<br>*Number of providers interviewed*: 6<br>*Providers' characteristics*: 2 males and 4 females, considered to be key | This 18-week on-site program intervention took place at the Danish residential weight-loss centre. The program consisted of group-based intensive structured group exercise and educational sessions exercise, diet (individual calorie intake was based on energy calculations for a normal weight person with a sedentary activity level), and an educational program. The educational program comprised lessons about nutrition, monitoring of food intake and instruction in behavioural techniques from cognitive therapy. The personal development component included a minimum of two individual conversations with one of the | Focus groups and interviews |

For peer review only

| | | | | | |
|---|---|---|---|---|---|
| | | | personnel; the director, the administrative executive, and the leaders of the main areas diet, exercise and personal development | psychotherapists, motivational meetings for all participants. | |
| *First Author*: Danielsen<br>*Year:* 2016<br>*Category*: B<br>*Country*: Norway | To explore the experiences of physical activity from a participant perspective prior to, during, and after an intensive inpatient lifestyle modification program, including a high volume of adapted physical activity for the treatment of severe obesity. | Both genders, with a variety in age, degree of obesity (BMI ≥ 40 or 35.0–39.9 with comorbidities), and weight loss during the inpatient stay, as well as variation in weight-loss maintenance and lack of maintenance | *Role:* Participant<br>*Number of participants*: 8<br>*Participants' characteristics*: 5 female, 3 male, aged 35 to 63 years; 6 married/cohabitants and 2 single; BMI ranged from 37 to 60 and body weight from 96 to 185 kg<br>*Socioeconomic and demographic characteristics*: NR<br>*Co-morbidities:* NR | The study was supplementary to a clinical controlled trial with a 1-year prospective follow-up study examining the effects of a 10- to 14-week inpatient lifestyle modification program for subjects with severe obesity. Two to three group-exercise sessions 5 days a week during the inpatient period, each lasting for a minimum of 45 minutes. Aiming to increase compliance, the activity was supervised by exercise scientists and physiotherapists, and the participants were introduced to adapted physical activity and equipment, and exercised together with other individuals with severe obesity. | Interviews |
| *First Author*: Groven<br>*Year:* 2010<br>*Category*: B<br>*Country*: Norway | To show how the training is experienced from a first-person perspective, namely | Female participants with obesity (BMI >35kg/m$^2$) from the weight-loss program in | *Role:* Participants<br>*Number of participants*: 5<br>*Participants' characteristics*: Aged 35-63 years and had been overweight for more than 10 years | Group-based weight-loss program in Norway, a program organized by physiotherapists in the primary health system. Offered to eight women struggling with obesity problems in a particular district of Norway for one | Interviews |

For peer review only

| | | | | | |
|---|---|---|---|---|---|
| | the patients themselves. | Norway | *Socioeconomic and demographic characteristics*: 3 married, 1 divorced and 1 widowed, 1 had a university degree, 2 had a college degree, and 2 had no formal education after high school. The women were at present or previously working in professions providing a service, or care, doing office work, or an academic job on various levels.<br>*Comorbidities*: Not reported | year. Total of 12 exercises were performed throughout the one-hour exercise program. The treatment also included group discussion for 1 hour per month. | |
| First Author: Jackson<br>Year: 2007<br>Category: B<br>Country: UK | To evaluate the effectiveness and acceptability of a specialist health visitor-led weight management clinic in primary care. | Patients with a BMI ≥30 | *Role: Participants*<br>*Number of participants: Unclear how many of 25 questionnaires returned provided written responses*<br>*Participants' characteristics: Not reported*<br>*Socioeconomic and demographic characteristics: Not reported*<br>*Comorbidities: Not reported* | Specialist health visitor-led intervention based on the Jan Felgens '12E2' model. The specialist health visitor sought to inspire participants through a combination of shared goal setting, reflection, problem-solving, positive affirmation and reinforcement. Consultations took place at the health centre and a relaxed, unhurried atmosphere was created. The average consultation time was 20 minutes (range 10–30 minutes), although the first appointment took approximately 1 hour and gave participants time to reflect on their lifestyles and to plan realistic goals | Open ended response options to questionnaire |

For peer review only

| | | | | | |
|---|---|---|---|---|---|
| | | | | for healthy eating and physical activity with the specialist health visitor. | |
| *First Author*: Janke<br>*Year:* 2012<br>*Category*: B<br>*Country*: USA | To gain insight into the patient's experience of comorbid chronic pain and obesity and to improve understanding of the behavioural linkages between the experience of pain, engagement in health behaviours, and obesity treatment outcomes. | Patients attending primary care clinics at a large Midwestern Veteran's Affairs hospital, > 18 years, BMI ≥25; weekly pain at an intensity ≥4 during the prior 3 months; and current diagnosis of a medical complaint associated with persistent pain | *Role:* Participant<br>*Number of participants*: 30<br>*Participants characteristics*: 24 male, 6 female<br>26 were age 50 or older, mean BMI was 36.8 (SD ± 8.9)<br>*Socioeconomic and demographic characteristics*: 22 were white, 20 had greater than a high school education, and 14 were unemployed or disabled while 13 were retired<br>*Comorbidities*: Measured on a scale of 0 to 10 (0 = none, 10 = worst imaginable), average pain intensity was 5.6 (SD ± 1.9) and average pain interference was 3.6 (SD ± 2.1) | The qualitative research project was designed to identify perceptions of those with both overweight/obesity and chronic pain regarding their experience of the course, impact, and treatment history of pain and weight symptoms; factors that might either ease or limit their ability to engage in health-promoting behaviours; and factors that facilitate or hinder engagement in treatments designed to achieve weight and/or pain control. | Focus groups and interviews |
| *First Author*: Jennings<br>*Year:* 2014<br>*Category*: B<br>*Country*: UK | To facilitate weight loss by implementing progressive and sustainable lifestyle changes, based on individually agreed goals over a 1-year | Adults (over 18 years) with obesity (BMI ≥40, or BMI ≥30 with obesity-related comorbidities and/or waist | *Role:* Participant<br>*Number of participants*: 12<br>*Participants' characteristics*: No specific data for qualitative analysed participants<br>*Socioeconomic and demographic characteristics*: No specific data for qualitative analysed participants. | The Fakenham weight management service (FWMS) provides Tier 3 services. This paper was service evaluation and had a cohort design recruited patients to a 1-year programme. | Focus groups |

| | | | | | |
|---|---|---|---|---|---|
| | programme. Focus groups were conducted to explore participants' experiences. | circumference ≥102 cm in men or ≥88 cm in women) | *Comorbidities*: No specific data for qualitative analysed participants. | | |
| *First Author*: Jimenez Lopez<br>*Year:* 2012<br>*Category*: B<br>*Country*: Mexico | To explore the motivations of patients involved in a with reduction programme, by analysing their experiences. | Patients with obesity included in a waiting list for bariatric surgery at a public hospital | *Role:* Participant<br>*Number of participants*: 10<br>*Participants' characteristics*: 2 Male, 8 women, mean age 45.2, mean BMI 41.3<br>*Socioeconomic and demographic characteristics*: NR<br>*Comorbidities*: NR | The dynamic of the intervention included the modification of dietary habits by a psychologic intervention, as recommended by the federal law of obesity management The focus group included ten patients with one investigator as an active observer, and 12 weekly sessions. | Focus groups |
| *First Author*: Kidd<br>*Year:* 2013<br>*Category*: B<br>*Country*: USA | To describe the effect of an 8-week mindful eating intervention on mindful eating, weight loss self-efficacy, depression, and biomarkers of weight in urban, underserved, women | Females (aged 30 years and older) with obesity (BMI ≥30kg/m$^2$) | *Role:* Participant<br>*Number of participants*:12<br>*Participants' characteristics*: Mean weight was 119.7kg (SD ± 16.87), BMI 44.7 (SD ±6.9) , Age ranged from 31–61 and averaged 51.8 years (SD ± 9.1)<br>*Socioeconomic and demographic characteristics*: 7 African American, 5 unemployed, and 4 married; 11 | The study used a mixed methods design. A one group pre-test/ post-test design examined the effect of an 8-week mindful eating intervention on the psychosocial variables and biomarkers. Weekly group sessions lasted 60 to 90 minutes and consisted of education and application of mindful eating principles. | Focus groups |

For peer review only

| | | | | | |
|---|---|---|---|---|---|
| | with obesity; and to identify themes of the lived experience of mindful eating. | | graduated from high school, 6 had college degrees<br>*Comorbidities*: Not reported | | |
| *First Author*: Pera<br>*Year:* 2016<br>*Category*: B<br>*Country*: Spain | To explore the meaning of obesity in elderly persons with knee osteoarthritis and to determine the factors that encourage or discourage weight loss. | Participants with obesity, knee osteoarthritis, and polypathology | *Role:* Participant<br>*Number of participants*: 10<br>*Participants characteristics*: 2 male, 8 female, mean age 67.23 (SD ±7.87), BMI 40.47 (SD ± 4.22), mean weight 92.35 kg (SD ± 8.93)<br>*Socioeconomic characteristics*:: 1 No education, 5 Primary (<5 years), 3 Secondary (<10 years), 1 Higher (>10 years), 2 Housewife, 8 Retired<br>*Comorbidities*: Mean number of co-morbidities 7.02 (SD ± 3.08) | The therapeutic education and functional preadaptation program was a 4-month program consisted of two 40-minute individual visits and three 90-minute group sessions for participants with obesity, knee osteoarthritis and polypathology. The program was designed following the methodology established for this type of program and was based on social learning theories. | Focus group |
| *First Author*: Counterweight<br>*Year:* 2008<br>*Category*: B<br>*Country*: UK | To explore key barriers and facilitators of practice and patient engagement in the Counterweight Programme and to describe key strategies used to | Patients with obesity in routine primary care | *Role:* Participant and Provider<br>*Number of participants*: 37 patients<br>*Number of providers*: weight management advisers (n = 7) in a focus group. In depth interviews were conducted with 15 PNs and 7 GPs across 11 practices.<br>*Participants' and/or providers characteristics*: Not reported | The Counterweight Project was set up to establish and improve obesity management in primary care by implementing an evidence-based weight management intervention that is practice focused. It was developed using theoretical models of behavioural change and, the best available methods from the published evidence. | *Participants*:<br>Interviews and focus groups<br><br>*Providers*:<br>Interviews and focus groups |

For peer review only

| | | | | | |
|---|---|---|---|---|---|
| | address barriers in the wider implementation of this weight management programme in UK primary care. | | *Socioeconomic and demographic characteristics*: Not reported<br>*Comorbidities reported*: Not reported | | |
| *First Author*: Shaw<br>*Year:* 2013<br>*Category*: B<br>*Country*: USA | To evaluate the acceptability, feasibility, and efficacy of daily text messages using regulatory focus theory to help individuals sustain weight loss. | Individuals had to own a mobile phone, be able to receive text messages, and have lost 5% of their body weight since entering the Duke Diet and Fitness Centre | *Role:* Participant<br>*Number of participants*: 60<br>*Participants' characteristics*: No specific data for qualitative analysed participants<br>*Socioeconomic and demographic characteristics*: No specific data for qualitative analysed participants.<br>*Comorbidities*: Not reported | Clients who received treatment at a residential weight loss management program that provides education, practical behavioural strategies, and ongoing support to make long-term changes at the Duke Diet and Fitness Centre (DFC), participated in this study. Participants were randomized to a promotion, prevention, or an attention control text message group after completion of a weight loss program. | Interviews |
| *First Author*: Sturgiss<br>*Year:* 2016<br>*Category*: B<br>*Country*: Australia | To describe the collaborative process used to develop an obesity management programme based on current Australian guidelines for GPs and their patients to | Health professionals involved in obesity management programme based on current Australian | *Role:* Provider<br>*Number of providers*: 38<br>*Providers' characteristics*: 15 GPs, 14 GPs registrar, 5 healthcare consumer representative, 2 representative bodies for chronic illness, 1 dietician, 1 psychologist | The Change Programme is a GP-delivered weight management programme that was developed based on Australian guidelines for the management of obesity in primary healthcare. It is based on one of the pillars of general practice—'patient centeredness'. No directive patient goals | Interviews and focus groups |

For peer review only

| | | | | | |
|---|---|---|---|---|---|
| | be used in primary care. | guidelines for GPs and their patients to be used in primary care | *Socioeconomic and demographic characteristics*: Not reported | were stated and the work was individualized. The programme consists of a GP handbook, patient workbook and computer template. This programme. The patients initially attended appointments every 2 weeks, with less frequent appointments as the programme continued. | |
| *First Author*: Sturgiss<br>*Year:* 2017<br>*Category*: B<br>*Country*: Australia | To assess the acceptability and feasibility of a GP-delivered weight management programme. | *Providers*: Fully qualified GPs from the Australian Capital Territory and New South Wales. | *Role:* Participant and Provider<br>*Number of providers*: 12<br>*Providers' characteristics*: The recruited GPs had an average 12 years of experience (range 4–30 years). The GPs worked in four urban practices and one rural practice.<br>*Number of patient participants*: 15 interviewed<br>*Participants' characteristics*: No specific data for qualitative analysed participants.<br>*Socioeconomic and demographic characteristics*: NR<br>*Comorbidities*: Not reported | See *Sturgiss 2016a* | Interviews |
| *First Author*: Sturgiss<br>*Year:* 2017<br>*Category*: B | To assess the self-efficacy and confidence of GPs | GPs working in 5 different general practices | *Role:* Provider<br>*Number of providers*: 12 | See *Sturgiss 2016a* | Interviews |

For peer review only

| | | | | | |
|---|---|---|---|---|---|
| *Country*: Australia | before and after implementing a weight management programme in their practice. | | *Providers' characteristics*: 12 GPs practised in 5 different general practices, 1 rural and 4 urban, and had between 4 and 30 years clinical experience<br>*Socioeconomic and demographic characteristics*: Not reported | | |
| *First Author*: Turner<br>*Year:* 2015<br>*Category*: B<br>*Country*: UK | To determine both physiological benefits and qualitative information, namely patient satisfaction, associated with the service. | Patients with obesity attending Multidisciplinary Weight Management Clinic (MDWMC) at Aneurin Bevan Hospital, Wales | *Role:* Participant<br>*Number of participants*: 180<br>*Participants characteristics*: 131 female, 49 male, ages ranged between 19 and 74<br>*Socioeconomic and demographic characteristics*: Not reported<br>*Comorbidities*: Not reported | Obesity management in Wales includes the provision of a 1:1 MDWMC. Strategic management of obesity in Wales is guided by The All Wales Obesity Pathway and recommends MDWMCs for people with obesity who have one or more co morbidities and who have tried several interventions without success, or who have complex emotional relationships with food. | Interviews |
| *First Author*: VanWormer<br>*Year:* 2010<br>*Category*: B<br>*Country*: USA | To examine the association between participant and program experiences and satisfaction with a weight loss intervention. | Adults (18 years or older) with obesity (BMI ≥ 32kg/m$^2$) employees of a managed care organization | *Role:* Participant<br>*Number of participants*: 78 (*not clear if all of these provided qualitative information*)<br>*Participants' characteristics*: Mean age 46.9 (SD ± 8.3), 70 female, 8 male, 55 married or living with a partner, 23 not married; body weight | Participants were randomly assigned to either an immediate or delayed start group. The intervention lasted 6 months. During treatment, participants received a telephone-based behavioural weight loss counselling intervention. The intervention included a course manual, behaviour change tools (e.g., food/ activity log, weight chart, pedometer), | Written responses to open ended response options within a questionnaire |

| | | | | | |
|---|---|---|---|---|---|
| | | | (kg) 106.2 (SD ± 16.32), BMI 38.3 (SD ± 5.2)<br>*Socioeconomic and demographic characteristics*: 36 college or graduate degree, 42 had less than college degree<br>*Comorbidities*: Not reported | and up to 10 telephone counselling calls from a registered dietitian and/or health educator. In addition, participants received a home tele monitoring scale and were instructed to weigh themselves daily. | |
| *First Author*: Young<br>*Year:* 2017<br>*Category*: B<br>*Country*: USA | To determine whether computerized provision of weight management with peer coaching is feasible to deliver, is acceptable to patients, and is more effective than in-person delivery or usual care. | Adults (18 years or older) with obesity (BMI > 30 or 28–30kg/m$^2$ with self-reported weight gain of at least 10 pounds in the last 3 months), with diagnosis of schizophrenia, schizoaffective disorder, bipolar disorder, major depressive disorder with psychosis, or posttraumatic | *Role:* Participant<br>*Number of participants*: 48 (24 randomized to WebMOVE and 24 randomized to MOVE SMI)<br>*Participants' characteristics*: No specific data for qualitative analysed participants<br>*Socioeconomic and demographic characteristics*: No specific data for qualitative analysed participants<br>*Comorbidities*: Not reported | Patients were randomized to a computerized weight management with peer coaching (Web- MOVE) or in-person clinician-led weight services, or usual care. Both active interventions offered the same educational content. WebMOVE weekly manualized peer coaching was delivered by phone and emphasized a strengths-based approach with motivational interviewing. MOVE SMI is an in-person weight management program led by a master's level mental health clinician. The program includes 24 sessions (8 individual and 16 group), each lasting 60 min. Usual care consisted of one educational handout on the benefits of weight loss, given to participants after randomization | Interviews |

| | | | | | |
|---|---|---|---|---|---|
| | | stress disorder; with prescribed an antipsychotic medication | | | |
| *First Author*: Zizzi<br>*Year:* 2016<br>*Category*: B<br>*Country*: USA | To explain how these services are perceived and received by participants in a community-based intervention so that specific recommendations can be made to health professionals working with similar populations and in similar settings. | West Virginia public employees' insurance agency weight management program (WMP), which is open to insured members that have a BMI >25 | *Role:* Participant<br>*Number of participants*: 567 (not clear how many provided qualitative data within the questionnaire<br>*Participants' characteristics*: 437 female, 130 male<br>*Socioeconomic and demographic characteristics*: Not reported<br>*Comorbidities*: Self-reported medication usage for 36% heart disease or high blood pressure, 31% anxiety or depression 21% high cholesterol, 12.7% diabetes, 9% sleep apnea | The WMP was a 2-year long benefit, and a $20 monthly co-payment that allowed participants to meet with a registered dietitian, exercise physiologist, and certified personal trainer at various point throughout their time in the program. The majority of individuals in the program also spoke with a health behaviour counsellor via telephone every 6 to 8 weeks. The WMP was offered at approximately 60 approved exercise facilities in West Virginia, such as YMCAs, wellness centres, fitness centres, and physical therapy clinics. | Written responses to open ended response options within a questionnaire |
| *First Author*: Owen Smith<br>*Year:* 2014<br>*Category*: C<br>*Country*: UK | To present a synthesis of data from two qualitative studies in which both the development and the experience of living with morbid obesity in men and | Individuals who met the United Kingdom NICE criteria for a morbid obesity (BMI ≥ 40, or 35 kg/m$^2$ with comorbidity), and | *Role:* Participant<br>*Number of participants*: 31 (Study 1 n = 13; Study 2 n = 18)<br>*Participants characteristics*: 9 males, 3 age group 20–29, 11 age group 30–39, 7 age group 40–49, 9 age group 50–59, 1 60+ age group | The qualitative approach to both studies, to investigate individual experiences of developing and living with morbid obesity. The first study (Study 1) as part of a broader investigation into patients' experiences of implicit and explicit rationing. The core results the second study (Study 2) as part of an ongoing | Interviews |

For peer review only

| | | | | | |
|---|---|---|---|---|---|
| | women were explored in depth. | sought access to treatment for their condition | *Socioeconomic and demographic characteristics*: 15 non manual employment, 5 manual employment, 5 homeworker/carer, 1 retired, 4 unemployed<br>*Comorbidities*: Not reported | longitudinal study investigating how clinicians communicate with patients about the availability of treatment in the context of resource scarcity. | |
| *First Author*: Owen Smith<br>*Year:* 2016<br>*Category*: C<br>*Country*: UK | To focus on experiences of accessing treatment for morbid obesity in primary care. | Patients and providers at a weight management clinic at a general hospital in the South West of England | *Role:* Participant and providers<br>*Number of participants*: 22 patients<br>*Number of providers*: 11<br>*Participants' characteristics*: 7 male, 15 female, 9 age group 20-39, 12 age group 40-59, 1 age 60+<br>*Socioeconomic and demographic characteristics*: 21 white British, 4 professional, 8 other non-manual, 3 manual, 6 unemployed, 1 retired<br>*Comorbidities*: 19 joint pain/mobility issues, 11 depression/other depressive disorder, 10 breathlessness/respiratory difficulties, 9 diabetes, 8 hypertension, 4 sleep apnoea, 4 cardiac problems, 3 fertility issues<br>*Number of providers*: 11 clinicians<br>*Providers' characteristics*: Clinician informants included consultants and | Data collection was undertaken using in-depth interviews with patients and clinicians working in a specialist secondary care facility, and analysis took a constant comparative approach. Patients were followed from before their first consultation in secondary care up to 36 months after referral. | Interviews |

For peer review only

| | | | | | |
|---|---|---|---|---|---|
| | | | three allied medical professionals who worked within the weight management service.<br>*Socioeconomic and demographic characteristics*: Not Reported | | |

Categories: A= Qualitative and mixed-methods studies linked to eligible RCTs, including any qualitative data reported as part of papers reporting quantitative outcomes; B= Qualitative and mixed-methods studies linked to ineligible RCTs and identified non-randomised intervention studies including any reported qualitative data; C= UK-based qualitative studies not linked to any specific interventions that draw on the experiences and perceptions of adults with BMI ≥35 (and/or providers involved in their care). ¥=Studies included in review 2 (long-term randomised and non-randomised studies conducted in UK). BMI= Body Mass Index, calculated weight (kg) / height (m2)

For peer review only

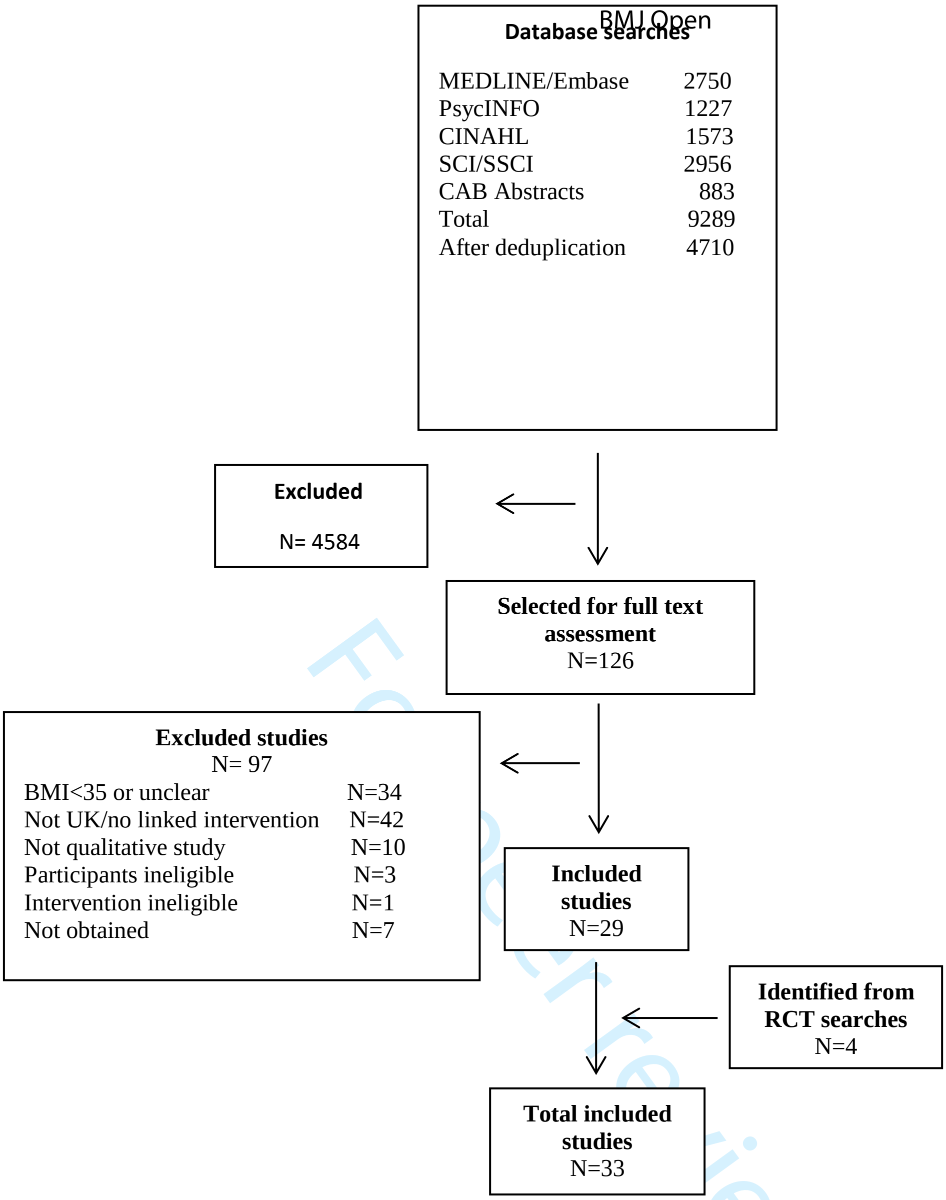


**S1 Figure Flow chart of included studies**

For peer review only

**REVIEW: Qualitative Studies**

**MEDLINE and EMBASE**

Ovid multifile search: http://shibboleth.ovid.com/

Database: Embase <1980 to 2017 Week 31>, Epub Ahead of Print, In-Process & Other Non-Indexed Citations, Ovid MEDLINE(R) Daily and Ovid MEDLINE(R) <1946 to Present>
26th April 2017

**Date of Search 26th April 2017**

1 qualitative research/
2 exp interviews as topic/ use ppez
3 exp interview/ use emez
4 focus groups/ use ppez
5 grounded theory/
6 (qualitative or interview$ or focus group?).tw,kw.
7 (ethno$ or grounded or thematic or realist or interpretive or narrative or discourse analysis or discursive or mixed method$).tw,kw.
8 or/1-7
9 *obesity/
10 morbid obesity/ use emez
11 exp obesity, morbid/ use ppez
12 (obese or obesity).tw,kw
13 or/9-12
14 Weight Loss/ use ppez
15 weight reduction/ use emez
16 (weight adj1 (los$ or reduc$ or maint$ or control$ or manag$)).tw,kw.
17 (reduc$ adj2 (bmi or body mass index)).tw.
18 (reduc$ adj2 (waist adj3 (ratio$ or circumference))).tw.
19 (obesity adj1 manag$).tw,kw
20 anti obesity.tw,kw
21 or/14-20

For peer review only

22 8 and 13 and 21

23 (obes$ adj3 (morbid$ or severe$ or extreme$)).tw,kw.

24 8 and (10 or 11 or 23)

25 22 or 24

26 25 not (abstract or letter or note or comment).pt.

27 remove duplicates from 26

**PsycINFO**

Ovid: http://shibboleth.ovid.com/

Database: PsycINFO <1987 to April Week 3 2017>

**Date of Search: 26th April 2017**

1 qualitative research/

2 interviews/

3 grounded theory/

4 discourse analysis/

5 ethnography/

6 (qualitative or interview$ or focus group?).tw,kw.

7 (ethno$ or grounded or thematic or realist or interpretive or narrative or discourse analysis or discursive or mixed method$).tw,kw.

8 or/1-7

9 obesity/ or body weight/

10 (obese or obesity).tw,kw

11 9 or 10

12 Weight Loss/ or weight control/

13 (weight adj1 (los$ or reduc$ or maint$ or control$ or manag$)).tw,kw.

14 (reduc$ adj2 (bmi or body mass index)).tw.

15 (reduc$ adj2 (waist adj3 (ratio$ or circumference))).tw

16 anti obesity.tw,kw.

17 (obesity adj1 manag$).tw,kw

18 or/12-17

19 8 and 11 and 18

20 (obes$ adj3 (morbid$ or severe$ or extreme$)).tw,kw.

For peer review only

21 8 and 20

22 "obesity (attitudes toward)"/

23 19 or 21 or 22

**CINAHL**

http://search.ebscohost.com

!981- 25th April 2017

**Date of Search: 25th April 2017**

S1 (MH "Qualitative Studies+")

S2 (MH "Interviews") OR (MH "Semi-Structured Interview") OR (MH "Structured Interview")

S3 (MH "Focus Groups")

S4 (MH "Narratives")

S5 TX qualitative OR TX interview* OR TX focus group*

S6 TX ( ethno* or grounded or thematic ) OR TX ( realist or interpretive or narrative ) OR TX ( discourse analysis or discursive or mixed method* )

S7 S1 OR S2 OR S3 OR S4 OR S5 OR S6

S8 (MH "Obesity") OR (MH "Obesity, Morbid")

S9 (MH "Body Weight")

S10 TX obese OR TX obesity

S11 S8 OR S9 OR S10

S12 (MH "Weight Control")

S13 (MH "Weight Loss")

S14 TX weight N1 los* OR TX weight N1 reduc* OR TX weight N1 maint* OR TX weight N1 control

S15 TX weight N1 manag* OR TX reduc* N2 bmi OR TX reduc* N2 body mass

S16 reduc* N2 waist ratio* OR TX reduc* N2 waist circumference TX

S17 S12 OR S13 OR S14 OR S15 OR S16

S18 (S7 AND S11 AND S17)

S19 (MH "Obesity, Morbid")

S20 TX obes* N3 morbid* OR TX obes* N3 severe OR TX obes* N3 extreme*

S21 S19 OR S20

For peer review only

S22  S7 AND S21

S23 (MH "Attitude to Obesity")

S24  S18 OR S22 OR S23

**Science Citation Index and Social Science Citation Index**

www.webofknowledge.com

1980 -  28th April 2017

**Date of Search: 28th April 2017**

# 1  TS=(qualitative or interview* or focus group)

# 2  TS=(ethno* or grounded or thematic or realist or interpretive or narrative or discourse analysis or discursive or mixed method*).

# 3  #1 OR #2

# 4  TS=(obesity or obese)

# 5   TS=(weight NEAR/1 los*) or TS=(weight NEAR/1 reduc*) or TS=(weight NEAR/1 maint*) or TS=(weight NEAR/1 control*) or TS=(weight NEAR/1 manag*).

# 6  TS=(reduc* NEAR/2 BMI) OR TS=(reduc* NEAR/2 body mass index)

# 7  TS=anti obesity

# 8  TS= (obesity NEAR/1 manag*)

# 9  #5 or #6 or #7 or #8

10  #3 AND #4 AND #9  *))) AND DOCUMENT TYPES: (Article)

**CAB Abstracts**

Ovid search: http://shibboleth.ovid.com/

Database: CAB Abstracts <1984 to 2017 Week 15>

**Date of Search: 26th April 2017**

1     qualitative analysis/

2     qualitative techniques/

3     (qualitative or interview$ or focus group?).tw.

4     (ethno$ or grounded or thematic or realist or interpretive or narrative or discourse analysis or discursive or mixed method$).tw.

5     or/1-4

For peer review only

6 obesity/

7 (obese or obesity).tw.

8 6 or 7

9 weight reduction/

10 (weight adj1 (los$ or reduc$ or maint$ or control$ or manag$)).tw.

11 (reduc$ adj2 (bmi or body mass index)).tw.

12 (reduc$ adj2 (waist adj3 (ratio$ or circumference))).tw.

13 (obesity adj1 manag$).tw

14 anti obesity.tw.

15 or/9-14

16 5 and 8 and 15

17 (obes$ adj3 (morbid$ or severe$ or extreme$)).tw.

18 5 and 17

19 16 or 18

For peer review only

# Enhancing transparency in reporting the synthesis of qualitative research: ENTREQ

**ENTREQ Statement: content and rationale**

The ENTREQ statement consists of 21 items grouped into five main domains: introduction, methods and methodology, literature search and selection, appraisal, and synthesis of findings (Table 1). For each item, a descriptor and examples are provided. Below we present a rationale for each domain and its associated items.

Table 1

**Enhancing transparency in reporting the synthesis of qualitative research: the ENTREQ statement**

| **No** | **Item** | **Guide and description** | |
|---|---|---|---|
| **1** | Aim | State the research question the synthesis addresses. | See Page 3 |
| **2** | Synthesis methodology | Identify the synthesis methodology or theoretical framework which underpins the synthesis, and describe the rationale for choice of methodology *(e.g. meta-ethnography, thematic synthesis, critical interpretive synthesis, grounded theory synthesis, realist synthesis, meta-aggregation, meta-study, framework synthesis).* | See Page 4 |
| **3** | Approach to searching | Indicate whether the search was pre-planned (*comprehensive search strategies to seek all available studies)* or iterative (*to seek all available concepts until they theoretical saturation is achieved)*. | See Page 3/4 |
| **4** | Inclusion criteria | Specify the inclusion/exclusion criteria *(e.g. in terms of population, language, year limits, type of publication, study type).* | See Page 3 |
| **5** | Data sources | Describe the information sources used (e.g. *electronic databases (MEDLINE, EMBASE, CINAHL, psycINFO, Econlit), grey literature databases (digital thesis, policy reports), relevant organisational websites,* | See Page 3 |

| No | Item | Guide and description | |
|---|---|---|---|
| | | *experts, information specialists, generic web searches (Google Scholar) hand searching, reference lists)* and when the searches conducted; provide the rationale for using the data sources. | |
| **6** | Electronic Search strategy | Describe the literature search *(e.g. provide electronic search strategies with population terms, clinical or health topic terms, experiential or social phenomena related terms, filters for qualitative research, and search limits).* | See Page 3 and S1 Appendix |
| **7** | Study screening methods | Describe the process of study screening and sifting *(e.g. title, abstract and full text review, number of independent reviewers who screened studies).* | See Page 3/4 |
| **8** | Study characteristics | Present the characteristics of the included studies *(e.g. year of publication, country, population, number of participants, data collection, methodology, analysis, research questions).* | See Page 6/7 and S1 Table |
| **9** | Study selection results | Identify the number of studies screened and provide reasons for study exclusion *(e,g, for comprehensive searching, provide numbers of studies screened and reasons for exclusion indicated in a figure/flowchart; for iterative searching describe reasons for study exclusion and inclusion based on modifications t the research question and/or contribution to theory development).* | See Figure 1, page 5 |
| **10** | Rationale for appraisal | Describe the rationale and approach used to appraise the included studies or selected findings *(e.g. assessment of conduct (validity and robustness), assessment of reporting (transparency), assessment of content and utility of the findings).* | See Page 5 |

| No | Item | Guide and description | |
|---|---|---|---|
| **11** | Appraisal items | State the tools, frameworks and criteria used to appraise the studies or selected findings *(e.g. Existing tools: CASP, QARI, COREQ, Mays and Pope* [25]*; reviewer developed tools; describe the domains assessed: research team, study design, data analysis and interpretations, reporting).* | See Page 5 |
| **12** | Appraisal process | Indicate whether the appraisal was conducted independently by more than one reviewer and if consensus was required. | See Page 5. Two reviewers initially assessed quality of included studies using the criteria proposed by Toye et al. During subsequent group discussions we continued to discuss and reflect on key aspects of quality. |
| **13** | Appraisal results | Present results of the quality assessment and indicate which articles, if any, were weighted/excluded based on the assessment and give the rationale. | Please see detail provided on pages 22-23 |
| **14** | Data extraction | Indicate which sections of the primary studies were analysed and how were the data extracted from the primary studies? *(e.g. all text under the headings "results /conclusions" were extracted electronically and entered into a computer software).* | See Page 4 ans S1 Table |
| **15** | Software | State the computer software used, if any. | N/A |
| **16** | Number of reviewers | Identify who was involved in coding and analysis. | See Pages 4 |
| **17** | Coding | Describe the process for coding of data *(e.g. line by line coding to search for concepts).* | See Page 4 |
| **18** | Study comparison | Describe how were comparisons made within and across studies *(e.g. subsequent studies were coded into pre-existing concepts, and new concepts were created when deemed necessary).* | See Page 4 and S1 Table |

| No | Item | Guide and description | |
|---|---|---|---|
| **19** | Derivation of themes | Explain whether the process of deriving the themes or constructs was inductive or deductive. | See page 4 |
| **20** | Quotations | Provide quotations from the primary studies to illustrate themes/constructs, and identify whether the quotations were participant quotations of the author's interpretation. | See Results section |
| **21** | Synthesis output | Present rich, compelling and useful results that go beyond a summary of the primary studies (e.g. *new interpretation, models of evidence, conceptual models, analytical framework, development of a new theory or construct).* | See Results and discussion section. |

For peer review only

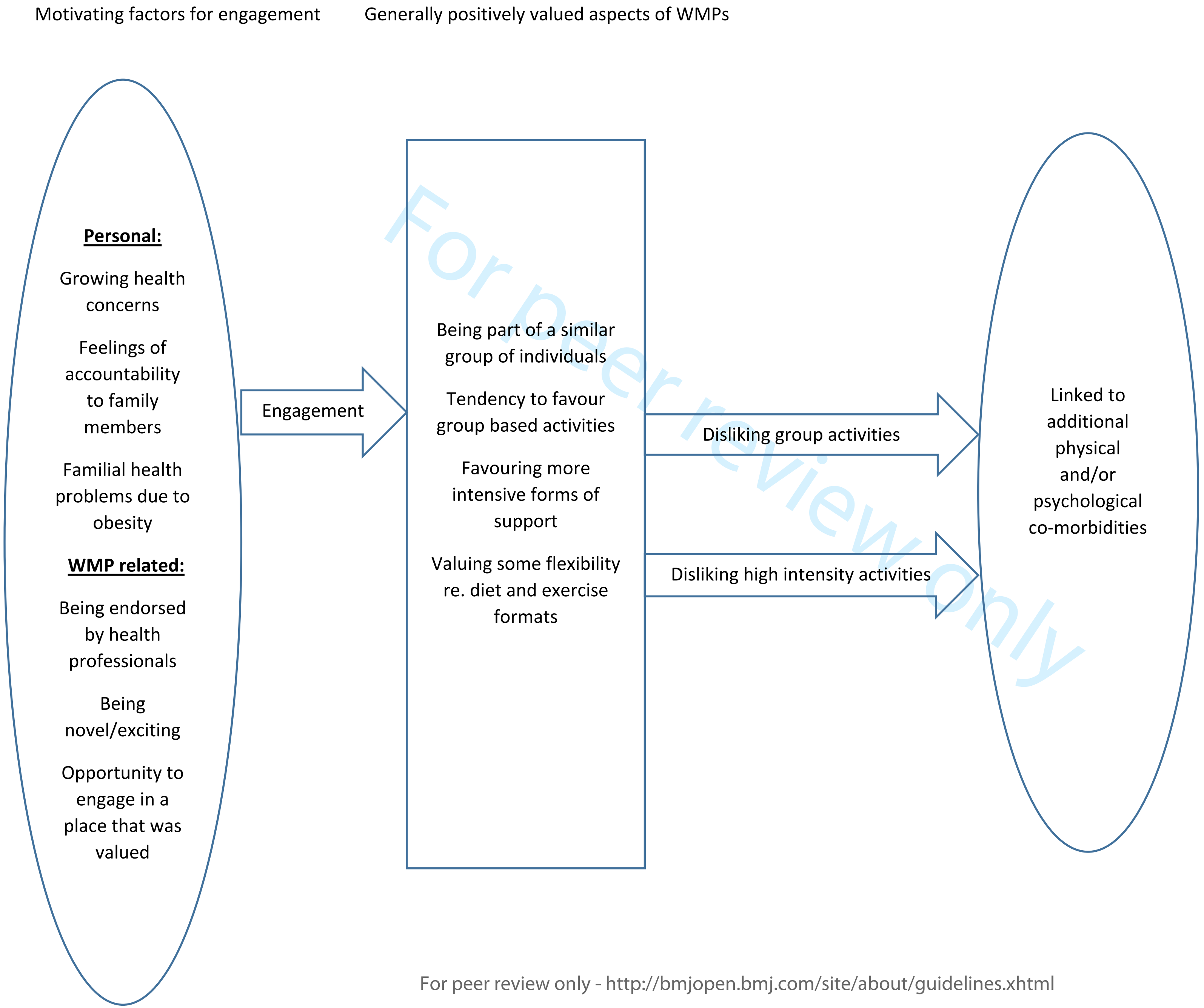
Motivating factors for engagement
Generally positively valued aspects of WMPs
Personal:
Growing health concerns
Feelings of accountability to family members
Familial health problems due to obesity
WMP related:
Being endorsed by health professionals
Being novel/exciting
Opportunity to engage in a place that was valued
Engagement
Being part of a similar group of individuals
Tendency to favour group based activities
Favouring more intensive forms of support
Valuing some flexibility re. diet and exercise formats
Disliking group activities
Disliking high intensity activities
Linked to additional physical and/or psychological co-morbidities

BMJ Open

# The acceptability and feasibility of weight management programmes for adults with severe obesity: A qualitative systematic review

| | |
|---|---|
| Journal: | *BMJ Open* |
| Manuscript ID | bmjopen-2019-029473.R2 |
| Article Type: | Research |
| Date Submitted by the Author: | 07-Aug-2019 |
| Complete List of Authors: | Skea, Zoë; Universit of Aberdeen, Health Services Research Unit<br>Aceves-Martins, Magaly; University of Aberdeen, Health Services Research Unit<br>Robertson, Clare; University of Aberdeen, Health Services Research Unit<br>De Bruin, M; University of Aberdeen,<br>Avenell, Alison; University of Aberdeen, Health Services Research Unit |
| <b>Primary Subject Heading</b>: | Public health |
| Secondary Subject Heading: | Nutrition and metabolism |
| Keywords: | QUALITATIVE RESEARCH, PUBLIC HEALTH, NUTRITION & DIETETICS |
| | |

SCHOLARONE™
Manuscripts

# The acceptability and feasibility of weight management programmes for adults with severe obesity: A qualitative systematic review

**Skea ZC, Aceves-Martins M, Robertson C, de Bruin M, Avenell A, and the REBALANCE team ***

**Author: Dr Zoë Christina Skea**

**Corresponding author: Dr Zoë Christina Skea, Health Services Research Unit, 3rd Floor, Health Sciences Building, University of Aberdeen, Aberdeen, AB25 2ZD, Tel: +44 (0) 1224 438153. email:z.skea@abdn.ac.uk. Fax: +44 (0) 1224 438165.**

**Co-authors: Dr Magaly Aceves-Martins, University of Aberdeen, Aberdeen, UK; Ms Clare Robertson, University of Aberdeen, Aberdeen, UK; Professor Marijn de Bruin, University of Aberdeen, Aberdeen; UK, and Radboud Institute for Health Sciences, The Netherlands; Professor Alison Avenell, University of Aberdeen, Aberdeen, UK.**

**Key words: Qualitative research; Severe obesity; Weight management programmes.**

**Word count: 7165 words**

## Abstract

### Objectives

To improve our understanding of the acceptability of behavioural weight management programmes (WMPs) for adults with severe obesity.

### Design

A systematic review of qualitative evidence.

### Data Sources

Medline, Embase, PsycINFO, CINAHL, SCI, SSCI and CAB abstracts were searched from 1964-May 2017.

### Eligibility Criteria

Papers that contained qualitative data from adults with BMI ≥ 35kg/m$^2$, (and/or the views of providers involved in their care) and considered issues about weight management.

### Data extraction and synthesis

Two reviewers read and systematically extracted data from the included papers which were compared, and contrasted according to emerging issues and themes. Papers were appraised for methodological rigour and theoretical relevance using Toye's proposed criteria for quality in relation to meta-ethnography.



For peer review only - http://bmjopen.bmj.com/site/about/guidelines.xhtml

**Results**

33 papers met our inclusion criteria from seven countries published 2007-2017. Findings were presented from a total of 644 participants and 153 programme providers.

Participants described being attracted to programmes that were perceived to be novel or exciting, as well as being endorsed by their health care provider. The sense of belonging to a group who shared similar issues, and who had similar physiques and personalities, was particularly important and seemed to foster a strong group identity and related accountability. Group based activities were enjoyed by many and participants preferred WMPs with more intensive support. However, some described struggling with physical activities (due to a range of physical co-morbidities) and not everyone enjoyed group interaction with others (sometimes due to various mental health co-morbidities). Although the mean BMI reported across the papers ranged from 36.8 - 44.7kg/m$^2$, no quotes from participants in any of the included papers were linked to specific detail regarding BMI status.

**Conclusions**

Although group-based interventions were favoured, people with severe obesity might be especially vulnerable to physical and mental co-morbidities which could inhibit engagement with certain intervention components.

**Strengths and limitations of this study**

- This is the first review of key findings from qualitative studies exploring views of Weight Management Programmes for adults with severe obesity (body mass index ≥35kg/m$^2$).
- Qualitative studies have a key role to play in understanding how factors facilitate or hinder the effectiveness of interventions, and how the process of interventions are perceived and implemented by users.
- Across the 33 papers, specific participant characteristics were inconsistently and poorly reported (if at all).
- Although the mean BMI reported across the papers ranged from 36.8 - 44.7kg/m$^2$, no quotes from participants in any of the included papers were linked to specific detail regarding BMI status.



For peer review only - http://bmjopen.bmj.com/site/about/guidelines.xhtml

## Introduction

There has been a continued increase in body mass index ≥35kg/m$^2$ (denoted here by the term 'severe obesity') in adults in the UK {1, 2}. As BMI increases, obesity-related comorbidities, social, psychological and economic consequences increase, with the potential need for greater support for help with weight loss. In the UK, having severe obesity, with or without comorbidities, may be a referral criterion for Tier 3 specialist weight management services in the obesity pathway, prior to Tier 4 services for bariatric surgery {3,4}. Effective weight-loss services may reduce the need for bariatric surgery, and could also increase the effectiveness of subsequent bariatric surgery {5}.Current NICE and SIGN guidance on weight management for obesity does not distinguish between obesity (BMI 30 to <35kg/m$^2$) and severe obesity (BMI ≥35kg/m$^2$); and public health guidance excludes evidence on weight-loss programmes for obese people with co-morbidities in the UK.{3,6,7} This implies that Tier 3 services are being created and money is being spent without an appropriate systematic review that clarifies what works for people with severe obesity (and their co-morbidities).

Qualitative studies have a key role to play in understanding how factors facilitate or hinder the effectiveness of interventions, and how the process of interventions are perceived and implemented by participants. This qualitative systematic review was conducted as part of a larger systematic review funded by the UK's National Institute for Health Research Health Technology Assessment Programme {8} and aimed to improve our understanding of the feasibility and acceptability of non-surgical weight management programmes (WMPs) for adults with severe obesity and programme providers. Previous qualitative reviews have been undertaken {9,10} but these have not focussed on WMPs that are designed for or include people with severe obesity.

Our broad initial research questions included "What is it like to engage with (or be a provider of) weight-loss interventions for adults with severe obesity?" and "What is it about interventions for adults with severe obesity that makes them helpful or unhelpful? Our review also considered issues around what might motivate people to decide to engage in such programmes.



For peer review only - http://bmjopen.bmj.com/site/about/guidelines.xhtml

This paper focuses on the main themes that emerged from the qualitative review of included studies. These themes shed light on 1) motivating factors for engagement; 2) components of WMPs participants described valuing; and 3) general challenges for engagement.

## Methods

### *Searching and identification of relevant studies*

A systematic search was conducted in June 2016 and updated during April/May 2017 for published papers that contained qualitative data from adults with BMI ≥ 35kg/m$^2$ (and/or the views of providers involved in their care) and considered issues relating to weight management (See S1 Appendix for search strategies and S1 ENTREQ Checklist). Two researchers (ZCS and MAM) independently screened titles, abstracts and selected full text papers. Where consensus could not be reached regarding eligibility, a discussion at a research team meeting took place.

We included studies that fitted into the following broad categories:

A. Qualitative and mixed-methods studies linked to eligible RCTs (from our other review), including any qualitative data reported as part of papers reporting quantitative outcomes;
B. Qualitative and mixed-methods studies linked to ineligible RCTs and identified non-randomised intervention studies including any reported qualitative data;
C. Qualitative studies not linked to specific interventions that drew on the experiences and perceptions of adults with BMI ≥35kg/m$^2$ (and/or providers involved in their care) providing they reported data specifically relating to views/experiences of strategies for weight loss.

### *Analysis and synthesis*

There are several approaches that can be used for synthesising the findings of qualitative studies.{11,12} Whilst being aware of the differing philosophical stances underlying various approaches to qualitative synthesis, we chose to adopt a pragmatic approach to our work in this area, which specifically aims to synthesise data that are relevant to informing policy and practice.{10} Our pragmatic approach drew on a ‘realist’ perspective{12,13} as we were concerned with trying to find out not only ‘what works’ for weight management for this group of adults and intervention providers, but also ‘for whom, and under what circumstances’. At the same time, our approach was informed by and used aspects of review methods such as thematic synthesis{14,15} and analytical approaches developed from methods of inquiry such as grounded theory.{15}



For peer review only - http://bmjopen.bmj.com/site/about/guidelines.xhtml

In order to collate and synthesise the available primary research, two authors (ZS, MAM) each read and systematically extracted data from the included papers, shared notes and discussed study findings and interpretations during a series of group meetings. The papers were initially organised according to the categories described above but, as inductive analysis progressed, papers were grouped, compared, and contrasted according to emerging issues and themes. We used a data extraction form, which summarised the main findings and original authors' discussion points and to note our own critical and interpretive comments on the papers. We then used these to facilitate the process of comparing and contrasting themes both within and across papers in order to develop cumulative insights into the mechanisms that are likely to impact on decisions to join and decisions to stay in or drop out of WMPs.

***Study quality***

The retrieved publications were appraised for methodological rigour and theoretical relevance independently by two reviewers using Toye's recently proposed criteria for quality in relation to meta-ethnography.{16} They suggest two core facets of quality for inclusion in syntheses of qualitative evidence, namely (1) Conceptual clarity: how clearly has the author articulated a concept that facilitates theoretical insight; (2) Interpretive rigour: what is the context of the interpretation; how inductive are the findings; has the interpretation been challenged? Two reviewers made notes regarding quality and results were compared and discussed.

***Patient and Public Involvement***

The REBALANCE Advisory Group included a mix of professional and lay members identified through team contacts (a clinician; dietician; policymaker; and 3 lay people who had all experience of severe obesity and use of related services) who offered advice throughout various stages of this project including during initial discussions around the choice of appropriate research questions to attempt to answer and areas of interest for this review, and our other suite of reviews which considered issues around intervention effectiveness and cost-effectiveness {8}. Results were disseminated at a final project meeting in 2018 at which the Advisory Group were present.

**Findings**



For peer review only - http://bmjopen.bmj.com/site/about/guidelines.xhtml

***Description of studies***

The database search produced 4710 abstracts (See S1 Figure for the PRISMA diagram providing information on the flow of studies through the review). Four additional papers were identified from included RCTs. In all, 33 papers met our inclusion criteria.{17,18,19,20,21,22,23,24,25,26,27,28,29,30,31,32,33,34,35,36,37,38,39,40,41,42,43,44,45,46,47,48,49}

The focus and key study characteristics of the 33 papers are outlined in S1 Table. The identified papers reported research conducted in seven countries (USA n=12; UK n=11; Norway n=3; Spain n=1; Canada n=2; Australia n=3; Mexico n=1), and published between 2007 and 2017. Seven papers were linked to broader intervention studies: {20,21,23,30,42,43,44} Seven papers were classed as Category A; 24 Category B; and 2 Category C. As can be seen from S1Table, the studies had varying aims, but all offered insights into stakeholder's perceptions of weight-loss strategies and programmes.

Although all the included papers provided some qualitative data for analysis, five of these provided qualitative data in the form of responses to open-ended survey questions within structured questionnaires.{22,32,37,46,49} Of those studies that used qualitative methods to collect their data, findings were presented from a total of 644 participants and 153 programme providers (mostly from interviews or focus group sessions).

Across the 33 papers, specific participant characteristics were inconsistently and poorly reported (if at all). Only 16 out of 33 papers provided any details. Information on sex was provided for 588 participants (out of 644 of those who specifically took part in qualitative evaluations) – 372 female; 216 male. Age was reported across 15 papers, with the range being 19-88 years. Six of these papers provided mean age with the range being 40.2–67 years. BMI for those involved in qualitative evaluations was reported in nine papers. Of those that provided a mean, this ranged from 36.8-44.7kg/m$^2$. Only four papers gave details of participants' ethnicity; from 188 participants, 35 were reported as being from ethnic or racial minorities. Furthermore, 14 papers specifically stated that study participants had a range of additional physical and/or serious mental health problems (e.g. osteoarthritis, chronic pain, schizophrenia, post-traumatic stress disorder). It was also apparent across other included papers from quotes and/or author comments that many participants had a range of similar comorbidities.



For peer review only - http://bmjopen.bmj.com/site/about/guidelines.xhtml

Although no included papers provided qualitative data from those who had been invited to join a programme, but had declined to take part at recruitment stage, some papers reported including participants who had not fully engaged with programme activities (being described as 'low users'; 'quitters' or 'drop outs').{17,24,25,36}.

The WMPs varied in the types and formats of support offered. Some programmes involved predominantly face to face interaction and activities with other participants and/or programme staff{24,27,29,31,32,33,34,35,40,45,47}. Two involved more remote forms of support (e.g. e-mail, telephone, text contact).{41,46} Other studies included and evaluated a mix of formats that also varied in intensity.{17,19,23,25,30,36,37,42,43,44,48,49}

Programmes incorporated a variety of tools and theories designed to support behaviour change and to help people lose weight. For example, tools such as diet diaries;{24,37} workbooks;{42,43,44} pedometers;{36,37,48} food logs; {17,47} conversation maps;{22} interactive monitoring devices;{46} social media group interaction; {19} daily text messages;{41} buddying;{37}. They also included a range of behaviour change theories (BCTs) and/or psychological support {20,21,26}. For example: goal setting;{32,33,36} motivational interviewing;{33} mindfulness;{35} self-determination theory based support;{24} regulatory focus theory;{41} self-regulation and cognitive behavioural techniques; {17,23,27,30,31,33,36,42,43,44}. Readiness to change and self-monitoring and feedback was also included{47}along with psychotherapeutic sessions;{34} emotional freedom therapy;{33}; neurolinguistic programming;{33} solution focussed therapy;{33} social learning theories.{40}

**Findings from the review – participants**

This section of the paper discusses the views of participants who chose to engage with WMPs. It considers motivating factors for their initial engagement; components of the WMPs that they described valuing; and then outlines more critical reflections and challenges for engagement (See S1 Conceptual diagram for an illustrative representation of key issues). The subsequent section of the paper discusses similar issues from the perspective of WMP providers.

***Motivating factors for engagement in WMPs***

Several papers provided insights into what had motivated prospective participants to take part in a specific WMP.{24,26,27,31,33,35,47}Important 'push' factors were sometimes personal to participants. For example, expressing a desire to do something about their weight/poor physical



For peer review only - http://bmjopen.bmj.com/site/about/guidelines.xhtml

fitness for themselves (e.g. as a result of growing health concerns and/or recent personal health scares) and also feelings of accountability to their families (e.g. stating that they wanted to be more engaged in activities with family members, as well as being there for family for as long as possible). Others recounted familial past experiences of health problems due to obesity or their own sudden and rapid weight gain due to mental health medication. For example:

***Recent personal health scares***

> *"I was told I was at risk of becoming diabetic." (No sample characteristics provided) {33}*

***Feelings of accountability to their families***

> *"I've had two kids in the last three years… that was part of the motivation… just getting fitter for my kids…I need to be aboot* [about] *for as long as possible" (Male).{31}*

***Familial past experiences of health problems due to obesity***

> *"My dad was a big guy and he developed diabetes, and he had to have surgeries and all kinds of stuff. I don't want to do that later in life." (intervention arm; no other sample characteristics provided). {47}*

***Sudden and rapid weight gain due to mental health medication***

> *"When I went on Zyprexa I gained a hundred pounds, very quickly. And that was really frustrating for me." (control arm; no other sample characteristics provided). {47}*

Some participants described motivators that were apparently related to certain aspects of the programme intervention itself. For example, because it was perceived as being endorsed as credible by health professionals; perceived as being novel and exciting in some key way, and also because it provided an opportunity to engage with the intervention in a place that was valued.{26,27,31}

> *"When I first went in there I thought this is great. I am going to diet at my doctor's surgery. Knowing that it was at my doctor's surgery gave me a big 'oof'." (no sample characteristics provided). [NB: We interpreted 'oof' as meaning that a WMP being endorsed by and delivered at the surgery gave this person a boost] {26}*

Although one paper highlighted that decisions to join a WMP were sometimes difficult and that some participants had expressed initial apprehension around taking part, {31} no included studies provided data about those who were invited to join but declined to take part at recruitment stage.



For peer review only - http://bmjopen.bmj.com/site/about/guidelines.xhtml

**Components of lifestyle programmes participants described liking or valuing**

We examined various aspects of WMPs that participants described valuing. In doing so, we were interested in the range of factors that might motivate those participants to join in the first place, and to continue to stay in the programme. We were also interested in the factors that they described as having assisted them to change aspects of their behaviour or ways of thinking. All but two papers were set within the context of a WMP. The two included papers that were not linked to a specific intervention{38,39} also provided data regarding perceptions of weight-loss strategies and engagement in diet and lifestyle programmes and were useful in this context. We found there was variation in what participants described as valuing within their WMP, demonstrating that a one size fits all approach is unlikely to be appropriate. We noted some key recurring themes in relation to what participants valued, and we grouped these around aspects that related to a) the overall setting or style of the programme; b) the people (both other participants and health professionals/support staff) within the programme setting; c) the type of interaction/support offered; d) dietary elements; e) physical activities; and d) programme tools and theories designed to support behaviour change. These are discussed below.

***a) The overall setting or style of the programme***

The overall setting of the programme was important for motivating people to decide to engage. It also seemed important for motivating them to stay in and keep going with the various intervention activities. Some participants described their programmes as being exciting or novel in that they perceived them to be different to interventions they had tried previously. For example, being focussed on physical activity rather than dieting{24} or being focussed on changing overall attitudes towards eating rather dieting *per se*;{35,43}. An important consideration was the extent to which they could 'relate' to the nature of the programme (including how it was presented to them at recruitment) and how well it appeared to match with their own identities and values:{24,31,35,39}

> *"…the main thing that drew us to it was because it's* [at a football club]*" (Male). {31}*

> *"I always think somebody approaching you one-on-one is better. They can post all the weight loss you know pamphlets out there…I was hooked right away because somebody took the time to really explain it and take her time to do that." (Female). {35}*



For peer review only - http://bmjopen.bmj.com/site/about/guidelines.xhtml

Several participants positively contrasted their overall perceptions of the WMPs with previous negative views towards other WMPs they had engaged with. For example, WMPs which were perceived as being too 'feminine' or in some ways humiliating and embarrassing, or being perceived to be overly preoccupied with dieting;{24,25,29,32,33,39}

> *"If you go to a slimming class you feel that you've made a fool of yourself or you get weighed and you've put on half a pound or a pound, and then you don't want to go back the next week so you don't go back." (Coaching group arm; no other sample characteristics provided). {25}*

> *"Well, I think it's (WHEEL) appealed to me because I won't be dieting…I am obsessed with dieting me." (Female) {24}*

> *"…spent many useless years at weight watchers with various leaders but never felt confident and in control or had the motivation I have now." (No sample characteristics provided). {32}*

***b) The importance of the people within the programme setting (for fostering a sense of accountability)***

A recurring theme was the value participants placed on perceiving themselves to be part of a like-minded group of individuals – individuals that faced similar issues, and who had similar physiques and personalities.{19,22,24,25,29,31,34} For example:

> *"I do not feel so ashamed of my body here. We are all in the same situation, you see, which is really nice" (Female). {29}*

These perceptions seemed to foster a strong group identity and related 'accountability' or responsibility to other participants and programme providers. This was apparently important for people in motivating them to stick with the programmes and to not let their fellow participants down by dropping out or not sustaining behaviour changes:{17,19,24,25,31,35,36,37,47}

> *"So, you didn't want to disappoint yourself, but you didn't want to disappoint … your friends now either." (No sample characteristics provided). {35}*



For peer review only - http://bmjopen.bmj.com/site/about/guidelines.xhtml

Many participants discussed the importance of their interactions with health care staff within the programmes.{17,24,25,27,29,32,33,34,35,37,40,43,45,49} They seemed to value the positive, friendly, and non-judgemental encouragement received. They also discussed feeling accountable to programme staff which helped with motivation. These aspects seemed to act as positive 'pulls' for staying in the intervention and helping to sustain behaviour change:

*"I think I just like talking to you [programme leader]. And I suppose I feel that if I don't do it [the programme] then I'm letting you down" (Female). {24}*

*"She is my motivator… and she makes me keep a record of my diet" (Female). {29}*

***c) The type of interaction/support offered***

Although not universal, many described particularly valuing the social interactivity of group based programme activities along with intensive support from/interaction with programme staff.{17,19,24,25,28,31,32,34,35,36,40,47,48} This appeared to function strongly as a motivator to maintain engagement with the WMPs by fostering feelings of accountability and by helping to ensure the achievement of pre-set goals:

*"Oh God I haven't done what I should of done and I promised to do it and I know that isn't what's supposed to spur you on but it I think it does" (Regular support group; no other sample characteristics provided).{25}*

*"[discussing feedback from programme staff]…great encouragement when the results are positive and a way to improve if the results are not so good." (No sample characteristics provided). {32}*

Participants discussed appreciating when the timing of support offered was flexible and could fit around their needs,{25,35,37}. Several wanted more support than was offered within the programmes (e.g. more frequent contact and for a longer duration than the programme currently allowed).{25,36,46,49} Many expressed concern about support ending post-intervention{24,25,29,35,41,47} with the suggestion that diminishing intensity of programme activities and/or programme cessation could cause problems for maintaining behaviour change patterns if group interaction and support were key parts of it:



For peer review only - http://bmjopen.bmj.com/site/about/guidelines.xhtml

> *"I cannot do it without her support, it just wouldn't work" (Female). {29}*

Some WMPs involved predominantly face to face interaction and activities with other participants and/or programme staff. {24,27,29,31,32,33,34,35,40,45,47} In contrast, others involved more remote forms of support (e.g. e-mail, telephone, text contact). {41,46} Some studies included and evaluated a mix of formats that varied in intensity. {17,19,23,25,30,36,37,42,43,44,48,49} Many participants discussed valuing the social interactivity of the inperson group-based activities{19,24,25,31,35,36,47}. Where it was discussed and compared, participants tended to value and desire human contact over more remote forms of support. {36,46} This preference seemed to be linked to incentivising people to stay committed to the various programmes and was important for making participants feel accountable to a likeminded group of individuals.

***d) Dietary elements***

Some WMPs provided detailed dietary advice regarding food choices, whilst others specifically described interventions as 'non-dietary' (nevertheless, incorporating behavioural change theories to support attitudinal changes towards food and eating patterns). Participants tended to describe valuing the flexibility and variety of diet formats. {24,35,36,40} This seemed important for helping them to 'normalise' and stabilise their eating habits, particularly as many had attempted diets over a period of many years (without success) leading them to develop negative and unhealthy relationships towards food.{24,35,36,40}

> *"The other programs told you not to eat this or that and you were afraid to go back if you hadn't lost weight and …they tell you that you can eat everything but you yourself have to control the amount…You make up the diet every day and that's very motivating" (Female).{40}*

***e) Physical activities***

All of the WMPs incorporated some attention to increasing physical activity. Whilst some participants described struggling to engage in exercise for a variety of reasons, many participants described the positive psychological and physical benefits they experienced from exercising.{19,24,29,33,47}



For peer review only - http://bmjopen.bmj.com/site/about/guidelines.xhtml

*"When I first started I could hardly walk…now I can walk 300-400 yards…if this project has done nothing else it has helped me to walk (no sample characteristics provided." (No sample characteristics provided).* {33}

When it was offered as part of the WMP, participants discussed valuing the flexibility of being able to choose from a variety of exercise formats and approaches. {24,36}

***f) Programme tools and behaviour change theories designed to support behaviour change***

Although not universally popular, {17,24,36,46,47} participants described the incorporation of tools, (e.g. food logs, goal setting, regular text messages, tele-monitoring devices and conversation maps) as being motivating, and helpful for the purposes of education and learning, describing how they helped to facilitate self-awareness of and reflection on eating and other behaviour patterns. {17,22,36,37,41,46,47,48,49}

*"I found it to be very enlightening. It made me start to look at foods differently It has given me a more conscious outlook on how to control my diabetes and the importance of exercise." (No sample characteristics provided). {22}*

*"What really helped me was having somebody go over the food log every day. That was the big thing." (No sample characteristics provided). {17}*

Participants discussed the positive psychological changes they experienced with regards to their relationship to food/body image, which seemed to relate to the BCTs employed within some of the WMPs (e.g. mindfulness and self-determination theory based support).{17,24,27,35}

**General challenges for engagement in WMPs**

Despite the numerous positive comments from within the data with regard to programme engagement, participation was not straightforward for everyone who took part. General challenges resulting in decreased engagement (or success) related to a number of factors. Sometimes, these involved the timing of clinic appointments;{37} cost of travel to appointments;{33,48} general low self-efficacy;{26} family members not being on board, such that behavioural changes were difficult to sustain;{34,47}. Others described factors which could be described as life getting in the way (e.g. holidays, social events, bad weather as disincentive to exercise). {47}



For peer review only - http://bmjopen.bmj.com/site/about/guidelines.xhtml

It was apparent that participants experienced a range of comorbidities, including some serious mental health issues. {18,19,36,37,38,39,46,47,48} Sometimes these specific illnesses presented challenges for motivation and continuing engagement, for example, feeling too ill to focus on weight/feeling too ill to care or to be motivated:{33,36,39,40,47}

> *"Because of the ME* [myalgic encephalopathy] *I'm sleeping fifteen or more hours a day, and so exercise is out of the question because I can't even walk to the end of the road." (Female).{38}*

**Critical reflections on specific components of WMPs**

***The type of interaction/support offered***

The social interactivity of group-based programme activities was not universally valued by all, with some describing a reluctance to discuss issues within a group setting.{19,27,28,40,45,48} This was perhaps particularly pertinent in studies where participants had additional mental health issues:

> *"I know the importance of the program is to be together, but at the beginning you don't know these people, some of us have problems interacting with people we don't know." (No sample characteristics provided. {19}*

> *"It's just I don't like to be around people." (No sample characteristics provided). {48}*

> *"I prefer to talk in private as I suffer from panic attacks." (No sample characteristics provided). {45}*

One study{44} included data that suggested some participants were guilty about using up what they perceived to be too much of their health care provider's time (in an intervention involving regular GP visits):

> *"I must admit I felt frequently embarrassed that I was taking up a lot of my GP's time." (No sample characteristics provided). {44}*

**Dietary elements and physical activities**

Although the majority of participants tended to describe valuing the flexibility and variety of the diet formats offered.{24,36,40,49} views were sometimes mixed with regard to diets, with a few



For peer review only - http://bmjopen.bmj.com/site/about/guidelines.xhtml

wanting more prescriptive and structured eating plans than were offered. Participants often discussed appreciating when programmes apparently emphasised changing attitudes towards food and eating over promoting a specific diet *per se* {24,36,40,49}:

> *"I think [having a set meal plan to follow] would have been to a certain extent easier at the beginning, but I don't think it would of actually adjusted my attitudes and thinking which it [POWeR+] has done (Male; 64 years; face-to-face support; high user)." (No sample characteristics provided).{36}*

However, sometimes participants stated that their programme (or their primary care providers) tended to over emphasise diet rather than, for example, addressing issues around exercise, sleep or addiction problems.{39,47}

> *"…there was no support counselling-wise as to why I have the issues I have with food…" (Male).{39}*

Whilst many participants described the positive psychological and physical benefits they experienced from exercising, {19,24,47} others described struggling to engage in exercise. Some described disliking the perceived high intensity of the exercises (e.g. feeling uncomfortable with sweating, {24,28,29}. Others discussed how their various physical or mental health comorbidities could prohibit them from full engagement in activities.{18,24,28,29,36,37,38,39,47}

> *"Exercise is the best [to lose weight] and I get all this physical therapy exercise and all of that just increases my pain, which reduces my desire to have any exercise." (No sample characteristics provided).{18}*

> *"I think for me, with my disability it was difficult to engage with some of the activities recommended." (No sample characteristics provided).{37}*

**Programme tools and BCTs designed to support behaviour change**

Participants suggested that many of the WMP's tools and theories were helpful to them for reflecting on their habits and behaviours and for helping them to positively change their attitudes. However, some participants described these tools as being somewhat intrusive and sometimes inflexible in nature. For example, some participants described disliking food logs and found food diaries/goal setting/daily self-weighing and the monitoring of exercise as excessive and too



For peer review only - http://bmjopen.bmj.com/site/about/guidelines.xhtml

confrontational.{24,36,46,47} Others reported that programme staff did not appropriately monitor and feedback on progress:{17}

> *"I mean no one ever looked at it [food diary]. No one ever asked for it. I just did all the work, like, for nothing because no one ever asked me for it." (No sample characteristics provided). {17}*

Others expressed frustration with the perceived inflexibility of tools designed to record behaviour and activities and to support behaviour change. For example, not being able to record life events and/or comorbidities that might help to explain lack of achievement regarding weight loss:{36,41}

> *"I thought that might be useful [to] have something [to] explain why things are going as they are going." (Female; 59 years, remote support; high user). {36}*

> *"I would want to tailor the messages [daily text messages] to the things that I was most struggling with." (No sample characteristics provided). {41}*

With regard to psychological support, two papers highlighted that some people wanted more counselling for non-direct weight issues, such as mental health, recognising that these additional problems had implications for weight management. {39,46} In contrast, although many participants discussed the various positive psychological changes they experienced (which seemed to relate to the BCTs/counselling employed within some of the WMPs), others found personal development classes challenging and confrontational and questioned their appropriateness:{27}

> *"I cannot benefit from it [the personal development classes]. I will never open up in that room and talk among others." (Male). {27}*

**Findings from the review – provider participants**

Ten of the included papers provided qualitative data from a range of WMP providers. {20,21,23,26,28,30,36,41,42,43} Seven of these papers were linked to one of three of the same interventions. Programme providers who provided qualitative data were described as primary care providers; {23,30} nurses; {36} GPs and consumer representatives;{43} GPs; {42,44} mental health care workers, dietitians, and nurses; {20,21} GPs, weight management advisors, practice nurses, {26} and key personnel working at a residential weight-loss centre. {27}



For peer review only - http://bmjopen.bmj.com/site/about/guidelines.xhtml

***General impressions of being involved in WMPs***

With the exception of one study, in which some GPs were reportedly less enthusiastic, {26} views about being involved in a WMP were generally very positive. Health professionals acknowledged that engagement was potentially very useful for them for facilitating a conversation around weight loss with participants - recognising that this can often be challenging in their everyday practices. {36,42,43,44}

However, the authors of one study{20} noted that discussions about weight tend to be embedded within the context of conversations about other health issues (rather than being discrete or stand-alone). They argued that this could act as a potential barrier with regards to the implementation of WMPs within primary care:

> *"I don't have patients that come to see me just for obesity or…just one thing…yes they're one of my diabetic patients but … we're talking about their cholesterol today or their blood pressure and their weight another day." (Nurse, no other sample characteristics provided).* {20}

***Motivating factors for participants'/provider engagement in WMPs***

One paper included some insights from the perspectives of programme providers about what motivated prospective participants to take part in a WMP. {23} Health care providers involved in WMP delivery described how they regarded participants' perceptions of their professional 'buy in' to the intervention study (i.e. endorsement) as important and influential regarding their decisions to take part. {23} One study (linked to two papers){23,30} reported unusual success at enrolling men which programme providers attributed to their endorsing it as a 'medical' programme:

> *"I think that [our affiliation with a research institution] helped make it into a legitimate type of program that* [our patients] *would have confidence in, not just one of these wild watermelon diets or things like that." (Primary Care Provider, no other sample characteristics provided).{23}*

In terms of disincentives towards retention in such WMPs, some providers reported that participants could sometimes have unrealistic expectations about weight loss, not fully understanding programme goals and commitment and wanting a "quick fix":



For peer review only - http://bmjopen.bmj.com/site/about/guidelines.xhtml

> *"What they wanted was a quick fix…They want to lose pounds very quickly. And it doesn't happen…"(GP, no other sample characteristics provided)* {26}

Only one study {26} provided data around barriers and facilitators to health professionals' own engagement with a specific WMP. They described how clinicians' pre-conceived beliefs and attitudes towards integrating WMPs into primary care settings were important and they noted that engaged practices (as opposed to less engaged practices) were characterised by active GP participation and 'buy in.'

***The importance of the people within the programme setting (for fostering a sense of accountability)***

In keeping with some key findings from participants across the included papers, programme providers reflected on the importance of WMPs for creating a sense of accountability both for themselves as professionals (by increasing their responsiveness and sensitivity to their participants' weight management plan and needs) and for participants continued engagement, motivation and success: {23,42}

> *"...I think it just made me be more sensitive...I've been kinda tryin' to dial it* [being tough on the patients*] down a little bit" (Primary Care Provider, no other sample characteristics provided) {23}*

Programme providers also recognised and reflected on the importance of establishing and maintaining good relationships and of giving positive reinforcement and encouragement and being supportive of weight loss efforts.{20,23,30,36}

***The types of interaction/support offered***

Several health care providers recognised that the intensity of interactions between programme staff and participants was important for motivating the latter to stay engaged and to sustain behaviour changes. {23,30} However, several provider participants raised concerns about the reality of this for their everyday clinical practice when time constraints were a real issue. {20,21,43} Other health care providers raised concerns around a lack of interdisciplinary working within clinic settings, which could inhibit their abilities to support weight loss, as well as lack of clarity with regard to professional role remits within teams:



For peer review only - http://bmjopen.bmj.com/site/about/guidelines.xhtml

*"I work with our RN all the time so on a daily basis we talk about things going back and forth but the others* [referring to dietitian and mental health workers] *I don't really see to be honest." (Nurse, no other sample characteristics provided). {21}*

Although providers in the above study{21} raised broad issues in their interviews relating to these barriers, they reflected positively on the study WMP for facilitating interdisciplinary collaboration.

**Views about mode of support**

When discussing preferred modes of support, health care providers considered issues regarding access and/or perceived effectiveness. Health providers in one primary care study{23} argued that telephone-delivered weight counselling was the most convenient for participants. In contrast, providers in another study (one that involved a residential WMP) {27} argued that face-to-face group interaction was essential and particularly useful for participants with severe obesity who often experience social isolation. In another primary care study, {36} views regarding mode of delivery of support were more mixed. Whilst recognising the practicalities of remote forms of support, programme providers (in this case nurses) argued that face-to-face interactions worked best for helping them connect more effectively and facilitated participant engagement and motivation. Some even stated that they did not regard remote support as support at all.

**Views about levels of provider engagement**

Health care providers in one study{23} stated that they played a fairly peripheral role in aspects of programme delivery and that sometimes this made it difficult for them to fully engage with their patient and to assess progress. They suggested that individualised feedback from other professionals involved in programme delivery (e.g. in this case weight-loss health coaches) would have been helpful. However, the study also reported that the majority of health care providers valued the fact that they played a limited role in the WMP, with time constraints and specific skill sets being raised as issues. Another study {36} raised related issues around level of provider engagement with aspects of the WMP. In this case, nurses discussed the perceived disadvantage of not being able to view the information provided to participants on the study website. Some stated that viewing this information would have allowed them to understand more fully, what participants were referring to in consultations. In one study,{43} GPs commented on and seemed to value the relatively 'loose' nature of the intervention design (in this case a weight management toolkit) as they considered it offered scope to enable them to tailor it to the individual and their community. Similarly, nurses in another study {36} expressed frustration around the lack of flexibility of their



For peer review only - http://bmjopen.bmj.com/site/about/guidelines.xhtml

intervention, both in terms of how they were supposed to behave (i.e. by not being directive) and also the lack of scope within the website to document individual issues. This was a concern raised by the participants themselves. Personnel in a residential WMP{27} specifically designed for people with severe obesity seemed to value having a very strict programme structure (in this case participants had to attend morning meetings, group activities, and eat six meals a day at fixed times). The general feeling amongst staff was that instilling this strictness on participants would facilitate behaviours that they would then seek to maintain at home.

**Views about intervention content**

Whilst some, (but not all), participants in one study{27} found personal development classes challenging and confrontational, providers in the same study consistently argued that personal development (i.e. focussing on personal factors such as self-knowledge and self-acceptance) was essential and crucially important for maintaining lifestyle changes longer term:

> *"It is important that they become aware of what in their life makes a difference in being obese or not." (Personnel, no other sample characteristics provided).{27}*

**Discussion**

*Principal findings*

This review synthesised findings from qualitative data relating to the views of adults with BMI ≥35kg/m$^2$ (and/or their health care providers) about engaging with WMPs. In summary, although there was variation expressed in views about the acceptability of various programme components (indicating the inappropriateness of a 'one size fits all' approach), there were, nevertheless, recurring themes around what both participant and programme providers described valuing and enjoying. Some of these key findings resonate with previous qualitative research with people with less severe obesity. {9,50}.

Participants in our review described being attracted to WMPs that were perceived to be novel or exciting in some key way, as well as perceived to have been endorsed by their health care providers (a view supported by programme providers themselves). The sense of belonging to a group of people who shared similar issues relating to weight and food, and who had similar physiques and personalities, was described as being particularly important to many participants. This seemed to



For peer review only - http://bmjopen.bmj.com/site/about/guidelines.xhtml

foster a strong group identity and related 'accountability', which seemed to help with motivation and continuing engagement.

Good relationships with programme providers were described as being highly valued, with ongoing encouragement and monitoring apparently important for facilitating motivation and behaviour change (a view also endorsed by the programme providers themselves). Group based programme activities were enjoyed by many participants along with intensive support from programme providers. This observation is supported in previous qualitative research with people with less severe obesity{9,50}. However, in our review, concerns were raised about the availability of continuing support post intervention.Similarly providers questioned the practicalities and logistics of integrating such intense support into their everyday clinical practices once the studies were completed.

Overall, both participants and programme providers valued having choice and flexibility. For example, participants welcomed flexibility around diet choices, flexibility around when face-to-face counselling sessions were scheduled, and welcomed personalised interventions. Similarly, some programme providers found the perceived lack of flexibility with various intervention components frustrating and prohibitive for supporting individualised care.

Those participants who described engaging in group discussions/therapy sessions and those who discussed engaging in exercises were mainly positive about their perceived benefits. Where it was discussed, participants valued the psychological input integrated into many interventions. This is a view supported in a study of user experiences of both Tier 2 and Tier 3 weight management services in England{50}. However, our review also highlighted that some participants did describe struggling with these aspects, with some describing them as particularly challenging. Some participants described difficulties with the various physical activities (because of a range of physical comorbidities). Not everyone enjoyed group interaction and discussions with others (sometimes apparently because they suffered from various mental health comorbidities).

*Practice Implications*

For intervention developers, it was clear from our review that social interaction activities tended to be valued. It was also apparent that ongoing encouragement and monitoring by programme providers was viewed as important for facilitating motivation and behaviour change. The waning intensity of programme activities and/or programme cessation could cause problems for



For peer review only - http://bmjopen.bmj.com/site/about/guidelines.xhtml

maintaining behaviour change patterns if group interaction and support were integral components. There is a need for WMPs to help consumers to establish support post intervention.

Intervention developers should be aware that people with severe obesity might be especially vulnerable to both physical and mental comorbidities, which could inhibit engagement with certain intervention components (e.g. group-based interaction; physical activities). This could inhibit their engagement with much fitter peers with fewer weight-related issues, or restrict their ability to undertake certain intervention components. This observation is less apparent in research with people with less severe obesity {9}. WMPs developers could consider including a choice of interaction styles/mix of physical activities to accommodate this.

*Strengths and limitations*

To our knowledge, this is the first review of key findings from qualitative studies exploring participants' perspectives of WMPs for adults with severe obesity. Our review has highlighted a range of important factors that have the potential to facilitate engagement with WMPs for this group.

We were interested in ascertaining the views of participants with severe obesity (people with BMI $\geq$35kg/m$^2$). Therefore, our inclusion criteria were that papers needed to state that participants in their respective studies (i.e. either in their qualitative evaluations or the intervention studies to which their qualitative evaluations were linked) had a mean BMI $\geq$35kg/m$^2$. Of those papers that only considered programme providers' views, these had to be linked to intervention studies where we could establish that included participants had a mean BMI $\geq$35kg/m$^2$. Only two papers stated that their respective WMPs were designed *specifically* for people with BMI $\geq$35kg/m$^2$. {24,42} Thus, across the papers, some people with BMI $<$35kg/m$^2$ would have been included. Quotes from participants were not linked to specific detail regarding BMI status, and so we cannot be certain that findings reflect exclusively the views of those with severe obesity.

Only nine papers linked participant quotes to sex; {24,27,29,31,35,36,38,39,40} only one to age status;{36} and none to socioeconomic/demographic characteristics, making it hard for us to consider whether any issues raised were particularly sensitive or pertinent to these aspects.



For peer review only - http://bmjopen.bmj.com/site/about/guidelines.xhtml

We know from a recent review of Tier 3 weight management interventions for adults with severe obesity that drop-out rates are very high (43-63%). {51} Only four of our included papers stated that some of the participants in their qualitative evaluations had been 'low users', 'quitters' or 'drop-outs'{17,24,25,36} and only one of these papers linked quotes directly to intervention usage status. {36} Although our findings highlighted a range of views with regard to the usefulness or otherwise of various intervention components, it is worth noting that participant sample characteristics within the included papers are skewed towards those who had chosen to engage and who had completed the various intervention activities.

Applying quality criteria to qualitative research remains a contentious issue and there is no consensus regarding whether and how this should be done {52,53}. Whilst authors of some qualitative evidence syntheses have chosen to exclude what they deem to be poor quality papers, we made the decision not to exclude any of the identified papers. We included 33 papers that each reported some qualitative data that met our inclusion criteria and addressed our key research questions. Although all included qualitative data, with regard to 'quality,' some were deemed richer than others in terms of data and insights. Some ranged from being exclusively qualitative studies providing rich data in our areas of interest, through to studies that were actually primarily quantitative with responses to open-ended survey questions. The five studies providing qualitative data in the form of responses to open-ended survey questions within structured questionnaires{22,32,37,46,49} were deemed less useful as they presented only very limited qualitative data and insights. Despite this variation in the overall level of quality, we believed it was more important to retain any relevant findings rather than disregard based on study quality. In doing so, we would argue that all 33 papers contributed useful elements to the collective whole and enabled us to develop our understanding of the issues of importance to people with BMI $\geq$35kg/m$^2$. We cannot exclude the possibility that unpublished service evaluations from within the NHS, that we failed to locate, might have been sources of rich data.

*Implications for research*

No papers included in our review provided qualitative data from those who had been invited to join a WMP but who had declined to take part. Only four papers reported including participants who had not fully engaged with all programme activities to varying degrees. The views of those who do not engage are important and should be a focus of future research. In terms of pointers for effective interventions, it is worth acknowledging that key findings will be skewed towards those who had



For peer review only - http://bmjopen.bmj.com/site/about/guidelines.xhtml

chosen to engage and who had completed the various intervention activities. This review also demonstrated that the qualitative research literature focusing specifically on lifestyle WMPs for people with very high BMIs is limited, particularly for people who are low-users or do not wish to engage with such services.

*Conclusions*

WMPs that are perceived to be novel or exciting and WMPs that are perceived to be endorsed by health care providers tend to be valued by participants. The sense of belonging to a group of people who share similar issues and characteristics seems particularly important, helping to foster a strong group identity and related 'accountability'- aiding motivation and continuing engagement. In-person group-based programme activities tend to be valued (over more remote forms of support), along with intensive support from programme providers. However, intervention developers should be aware that people with severe obesity might be especially vulnerable to both physical and mental co-morbidities that could inhibit engagement with certain intervention components.

Supporting Information

S1 Appendix: Search strategies

S1 ENTREQ Checklist

S1 Table: Characteristics of included studies

S1 Figure

S1 Conceptual Diagram

**Acknowledgements**

*REBALANCE Project management team Elisabet Jacobsen[1], Dwayne Boyers[2], David Cooper[1], Lise Retat[3], Paul Aveyard[4], Fiona Stewart[1], Graeme MacLennan[1], Laura Webber[3], Emily Corbould[3], Benshuai Xu[3], Abbygail Jaccard[3], Bonnie Boyle[1], Eilidh Duncan[1], Michal Shimonovich[1], Cynthia Fraser[1], Lara Kemp[1].

REBALANCE Advisory Group for all their advice and support during this project: Margaret Watson, Lorna Van Lierop, Richard Clarke, Jennifer Logue, Laura Stewart, Richard Welbourn, Jamie Blackshaw, Su Sethi.

[1]Health Economics Research Unit, University of Aberdeen, Aberdeen, UK

[2]Health Services Research Unit, University of Aberdeen, Aberdeen, UK

[3]UK Health Forum, Fleetbank House, Salisbury Square, London, UK



For peer review only - http://bmjopen.bmj.com/site/about/guidelines.xhtml

[4]Nuffield Department of Primary Care Health Sciences, Oxford University, Oxford, UK

## Contributors

AA and ZCS conceived the study idea for the qualitative systematic review. ZCS and MAM screened all titles and abstracts. ZCS and MAM conducted the data analysis and ZCS wrote the initial and subsequent manuscript drafts. AA, ZCS, MAM, CR, MdB contributed critically to discussions about interpretation of data and revisions of manuscript drafts. AA, ZCS, MAM, CR, MdB approved the final version.

## Funding

The project was funded by the NIHR Health Technology Assessment Programme (Project number: 15/09/04). See the HTA Programme website for further project information.

The views and opinions expressed therein are those of the authors and do not necessarily reflect those of the Department of Health, or the funders that provide institutional support for the authors of this report.

## Data sharing statement

This is a review of published studies which are available to access through the relevant journals.

## Competing interests statement

There are no competing interests for any author.

1. Welbourn R, Hopkins J, Dixon JB, et al on behalf of the Guidance Development Group. Commissioning guidance for weight assessment and management in adults and children with severe complex obesity. Obes Rev 2017.
2. Swancutt D, Tarrant M, Pinkney J. H. How Group-Based Interventions Can Improve Services for People with Severe Obesity. Current Obesity Reports 2019; 8:333–339.
3. National Institute for Health and Care Excellence. Obesity: identification, assessment and management of overweight and obesity in children, young people and adults. (Partial update of CG43). 2014. http://www.nice.org.uk/guidance/cg189
4. Royal College of Surgeons. Commissioning guide: weight assessment and management clinics (tier 3), 2014. https://www.rcseng.ac.uk/healthcare-bodies/docs/weight-assessment-and-management-tier-3-services.
5. The Look AHEAD Research Group. Cardiovascular effects of intensive lifestyle intervention in type 2 diabetes. N Eng J Med 2013;369:145-54.
6. Scottish Intercollegiate Guidelines Network. Management of obesity. Edinburgh; SIGN: 2010. http://www.sign.ac.uk/guidelines/fulltext/115/index.html
7. National Institute for Health and Care Excellence. Managing overweight and obesity in adults—lifestyle weight management services. (Public Health Guideline PH53).2014



For peer review only - http://bmjopen.bmj.com/site/about/guidelines.xhtml

8. Avenell A, Robertson C, Skea ZC, Jacobsen E, Boyers D, Cooper D et al. Bariatric surgery, lifestyle interventions and orlistat for severe obesity: The REBALANCE mixed-methods systematic review and economic evaluation. Health Technology Assessment, vol 22,no.68.

9. Sutcliffe K, Richardson M, Rees R, Burchett H, Melendez-Torres GJ, Stansfield C, Thomas J (2016) What are the critical features of successful Tier 2 weight management programmes for adults? A systematic review to identify the programme characteristics, and combinations of characteristics, that are associated with successful weight loss. London: EPPI-Centre, Social Science Research Unit, UCL Institute of Education, University College London.
10. Robertson C, Archibald D, Avenell A, Douglas F, Hoddinott P, van Teijlingen E, et al. Systematic reviews of and integrated report on the quantitative, qualitative and economic evidence base for the management of obesity in men. Health Technol Assess 2014;13:35.
11. Barnett-Page E, Thomas J. Methods for the synthesis of qualitative research: A critical review. *BMC Med Res Method* 2009;**9:**59**.**
12. Spencer L, Ritchie J, Lewis J, Dillon L. Quality in qualitative evaluation: a framework for assessing research evidence. London: Cabinet Office; 2003
13. Pawson R. Evidence-based policy: a realist perspective. London: Sage; 2006
14. Thomas J, Harden A. Methods for the thematic synthesis of qualitative research in systematic reviews. *BMC Med Res Method* 2008;**8:**45.
15. Harden A, Brunton G, Fletcher A, Oakley A. Teenage pregnancy and social disadvantage: systematic review integrating controlled trials and qualitative studies. *BMJ* 2009;**339:**b4254.
16. Toye F, Seers K, Allcock N, Briggs M, Carr E, Andrews J, et al. 'Trying to pin down jelly' - exploring intuitive processes in quality assessment for meta-ethnography. *BMC Med Res Method* 2013;**13:**46.
17. Abildso C, Zizzi S, Gilleland D, Thomas J, Bonner D. A mixed methods evaluation of a 12-week insurance-sponsored weight management program incorporating cognitive-behavioral counseling. *J Mix Methods Res* 2010;**4:**278-94.
18. Amy Janke E, Kozak AT. "The more pain I have, the more I want to eat": obesity in the context of chronic pain. *Obesity* 2012;**20:**2027-34.
19. Aschbrenner KA, Naslund JA, Bartels SJ. A Mixed Methods Study of Peer-to-Peer Support in a Group-Based Lifestyle Intervention for Adults With Serious Mental Illness. *Psychiatr Rehabil J* 2016;**39:**328-34.
20. Asselin J, Osunlana AM, Ogunleye AA, Sharma AM, Campbell-Scherer D. Missing an opportunity: the embedded nature of weight management in primary care. *Clin Obes* 2015;**5:**325-32.
21. Asselin J, Osunlana AM, Ogunleye AA, Sharma AM, Campbell-Scherer D. Challenges in interdisciplinary weight management in primary care: lessons learned from the 5As Team study. *Clin Obes* 2016;**6:**124-32.
22. Barham K, West S, Trief P, Morrow C, Wade M, Weinstock RS. Diabetes prevention and control in the workplace: a pilot project for county employees. *JPHMP* 2011;**17:**233-41.
23. Bennett WL, Gudzune KA, Appel LJ, Clark JM. Insights from the POWER practice-based weight loss trial: a focus group study on the PCP's role in weight management. *J Gen Intern Med* 2014;**29:**50-8.
24. Borkoles E, Carroll S, Clough P, Polman RCJ. Effect of a non-dieting lifestyle randomised control trial on psychological well-being and weight management in morbidly obese pre-menopausal women. *Maturitas* 2016;**83:**51-8.
25. Bradbury K, Dennison L, Little P, Yardley L. Using mixed methods to develop and evaluate an online weight management intervention. *Br J Health Psychol* 2015;**20:**45-55.
26. Counterweight Project T, McQuigg M, Brown JE, Broom JI, Laws RA, Reckless JP, et al. Engaging patients, clinicians and health funders in weight management: the Counterweight Programme. *Fam Pract* 2008;**25 Suppl 1:**i79-86.



For peer review only - http://bmjopen.bmj.com/site/about/guidelines.xhtml

27. Dahl U, Rise MB, Kulseng B, Steinsbekk A. Personnel and participant experiences of a residential weight-loss program. A qualitative study. *PLoS ONE* 2014;**9:**e100226.
28. Danielsen KK, Sundgot-Borgen J, Rugseth G. Severe Obesity and the Ambivalence of Attending Physical Activity: Exploring Lived Experiences. *Qual Health Res* 2016;**26:**685-96.
29. Groven KS, Engelsrud G. Dilemmas in the process of weight reduction: Exploring how women experience training as a means of losing weight. *Int J Qual Stud Health Well-being* 2010;**5**.
30. Gudzune KA, Clark JM, Appel LJ, Bennett WL. Primary care providers' communication with patients during weight counseling: a focus group study. *Patient Educ Couns* 2012;**89:**152-7.
31. Hunt K, Gray CM, Maclean A, Smillie S, Bunn C, Wyke S. Do weight management programmes delivered at professional football clubs attract and engage high risk men? A mixed-methods study. *BMC Public Health* 2014;**14:**50.
32. Jackson C, Coe A, Cheater FM, Wroe S. Specialist health visitor-led weight management intervention in primary care: exploratory evaluation. *J Adv Nurs* 2007;**58:**23-34.
33. Jennings A, Hughes CA, Kumaravel B, Bachmann MO, Steel N, Capehorn M, et al. Evaluation of a multidisciplinary Tier 3 weight management service for adults with morbid obesity, or obesity and comorbidities, based in primary care. *Clin Obes* 2014;**4:**254-66.
34. Jimenez-Lopez JL, Maldonado-Guzman ME, Flores-Perez Pasten L, Deciga-Garcia E. [Reasons for losing weight: Why have programed support?]. *Rev Med Inst Mex Seguro Soc* 2012;**50:**407-12.
35. Kidd LI, Graor CH, Murrock CJ. A mindful eating group intervention for obese women: a mixed methods feasibility study. *Arch Psychiatric Nurs* 2013;**27:**211-8.
36. Little P, Stuart B, Richard Hobbs FD, Kelly J, Smith ER, Bradbury KJ, et al. Randomised controlled trial and economic analysis of an internet-based weight management programme: POWeR+ (Positive Online Weight Reduction). *Health Technol Assess* 2017;**21:**1-61.
37. McRobbie H, Hajek P, Peerbux S, Kahan BC, Eldridge S, Trepel D, et al. Tackling obesity in areas of high social deprivation: clinical effectiveness and cost-effectiveness of a task-based weight management group programme - a randomised controlled trial and economic evaluation. *Health Technol Assess* 2016;**20:**79.
38. Owen-Smith A, Donovan J, Coast J. "Vicious circles": the development of morbid obesity. *Qual Health Res* 2014;**24:**1212-20.
39. Owen-Smith A, Donovan J, Coast J. Experiences of accessing obesity surgery on the NHS: a qualitative study. *J Public Health* 2016;**31:**31.
40. Pera PI, Ferrer MCO, Juarez MN, Juarez EN, Soler LM, Matheu CL, et al. Obesity, knee osteoarthritis, and polypathology: Factors favoring weight loss in older people. *Patient Prefer Adher* 2016;**10:**957-65.
41. Shaw RJ, Bosworth HB, Silva SS, Lipkus IM, Davis LL, Sha RS, et al. Mobile health messages help sustain recent weight loss. *Am J Med* 2013;**126:**1002-9.
42. Sturgiss E, Haesler E, Elmitt N, van Weel C, Douglas K. Increasing general practitioners' confidence and self-efficacy in managing obesity: a mixed methods study. *BMJ Open* 2017;**7:**e014314.
43. Sturgiss EA, Douglas K. A collaborative process for developing a weight management toolkit for general practitioners in Australia-an intervention development study using the Knowledge To Action framework. *Pilot Feasib Stud* 2016;**2:**20.
44. Sturgiss EA, Elmitt N, Haesler E, van Weelc C, Douglas K. Feasibility and acceptability of a physician delivered weight management programme. *Fam Pract* 2017;**34:**43-8.
45. Turner D, Haboubi N. Qualitative and Quantitative Outcomes of a 1:1 Multidisciplinary Weight Management Clinic. *Healthcare* 2015;**3:**429-51.



For peer review only - http://bmjopen.bmj.com/site/about/guidelines.xhtml

46. VanWormer JJ, Martinez AM, Cosentino D, Pronk NP. Satisfaction with a weight loss program: what matters? *Am J Health Promot* 2010;**24:**238-45.
47. Yarborough BJ, Stumbo SP, Yarborough MT, Young TJ, Green CA. Improving lifestyle interventions for people with serious mental illnesses: Qualitative results from the STRIDE study. *Psychiatr Rehabil J* 2016;**39:**33-41.
48. Young AS, Cohen AN, Goldberg R, Hellemann G, Kreyenbuhl J, Niv N, et al. Improving Weight in People with Serious Mental Illness: The Effectiveness of Computerized Services with Peer Coaches. *J Gen Intern Med* 2017;**32:**48-55.
49. Zizzi S, Kadushin P, Michel J, Abildso C. Client experiences with dietary, exercise, and behavioral services in a community-based weight management program. *Health Promot Pract* 2016;**17:**98-106.
50. Guzelegun N, Sissoko F, Jeong Ko U, Harrington J, Lanford K, Coulton V, et al. Qualitative opportunities into user experiences of tier 2 and tier 3 weight management services: Public Health England; 2017. https://www.gov.uk/government/publications/weight-management-services-insights-into-user-experiences [Accessed April 2018]
51. Brown 2017 Brown TJ, O'Malley C, Blackshaw J, Coulton V, Tedstone A, Summerbell C, et al. Exploring the evidence base for Tier 3 weight management interventions for adults: a systematic review. *Clin Obes* 2017;**7:**260-72.
52. Campbell R, Pound P, Morgan M, Daker-White G, Britten N, Pill R, Yardley L, Pope C, Donovan J: Evaluating meta-ethnography: systematic analysis and synthesis of qualitative research. Health Technol Assess 2011, 15(43):1–164. 3.
53. Dixon-Woods M, Sutton A, Shaw R, Miller T, Smith J, Young B, Bonas S, Booth A, Jones D: Appraising qualitative research for inclusion in systematic reviews: a quantitative and qualitative comparison of three methods. J Health Serv Res Policy 2007, 12(1):42–47

For peer review only



For peer review only - http://bmjopen.bmj.com/site/about/guidelines.xhtml

**S1 Table Characteristics of the included qualitative studies**

| Study | Aim (as described within the papers) | Condition of Focus | Participants Characteristics | Details of intervention | Qualitative data collection methods |
|---|---|---|---|---|---|
| *First Author*: Bennett<br>*Year:* 2014<br>*Category*: A<br>*Country*: USA | To understand primary care providers' (PCPs) perspectives about their role in the intervention and in their patients' weight loss, thereby providing insights to inform best practices in developing practice-based weight management programmes. | Patients with obesity in their usual care practices. | *Role:* Provider<br>*Number providers interviewed*: 26 PCPs<br>*Providers' characteristics*: 15 female, 11 male, 24 physicians, 2 nurse practitioners, and 20 had internal medicine training. The mean time in practice was 16 years (SD ± 11.7), and mean number of patients in the trial was 11.1 (SD ± 6.8)<br>*Socioeconomic and demographic characteristics*: 15 White, 6 Asian/Pacific Islander, 3 Black, 2 Other | The Practice-based Opportunities for Weight Reduction (POWER) was a 24 month trial that had two intervention groups (by phone and face-to-face) in which weight-loss health coaches (not PCPs) provided education and positive reinforcement. Participants in both intervention arms had access to the same online educational modules, self-monitoring tools and received both automated and individualized e-mails. Participants in the control arm met with a weight loss health coach at the time of randomization and, if desired, after the final data collection visit. They also received brochures along with a list of recommended weight loss websites. | Focus groups |
| *First Author*: Bradbury<br>*Year:* 2015<br>*Category*: A | To explore helpful (and unhelpful) aspects of coaching; | Participants with obesity. | *Role:* Participant<br>*Number of participants*: 58. | Positive Online Weight Reduction (POWeR) is an e-health intervention designed to produce sustainable weight | Interviews |

For peer review only

| | | | | | |
|---|---|---|---|---|---|
| *Country*: UK | the experiences of POWeR and the accompanying coaching, including what aspects people found most helpful, unhelpful, appealing or unappealing, and what factors seemed to influence whether participants continued to follow POWeR. | | Planning and development stages: 16 participants;<br>Feasibility stage: 23 participants;<br>Community trial 19 participants.<br>*Participants' characteristics*: From the community trial: age range 34-68, Participants were sampled from both the coaching arm (10 female, four male) and Web only arm (four female, one male) and varied in their usage of POWeR.<br>*Socioeconomic and demographic characteristics*: NR<br>*Comorbidities*: NR | management. POWeR consisted of 12 sessions which taught users self-regulation skills in order for them to become their own personal health trainer. Patients were randomized to either usual care, the POWeR website, POWeR accompanied by basic nurse support, or POWeR with regular nurse support. The nurse support was mainly delivered face to face, although telephone and email support could also be provided. | |
| *First Author*: Gudzune<br>*Year:* 2012<br>*Category*: A<br>*Country*: USA | To explore PCPs' usual practices as part of weight counselling to identify how PCPs communicate with their patients about weight loss. | Patients with obesity in their usual care practices | See *Bennett 2014* | See *Bennett 2014* | Focus groups |
| *First Author*: Hunt<br>*Year:* 2014<br>*Category*: A<br>*Country*: UK | To report the characteristics of men participating in a randomised | Men with obesity (BMI > 28kg/m$^2$), age 35–65 at high | *Role:* Participant<br>*Number of participants*: 63 men (who had attended at least six FFIT sessions of the programme). | Football Fans in Training (FFIT) is a men-only, evidence-based, 12-session, weight management and physical activity group programme with subsequent | Focus groups |

For peer review only - http://bmjopen.bmj.com/site/about/guidelines.xhtml

| | | | | | |
|---|---|---|---|---|---|
| | controlled trial of a weight management programme designed specifically to attract men, and, secondly, their accounts of why they decided to participate in the programme. | risk of ill-health due to obesity | *Participants characteristics*: No specific data for qualitative analysed participants<br>*Socioeconomic and demographic characteristics*: NR<br>*Comorbidities reported*: NR | minimal-contact weight loss maintenance support delivered free of charge at Scotland's top professional football clubs by community coaches trained in diet, nutrition, physical activity and behaviour change techniques to a standard programme delivery protocol. | |
| *First Author*: Little<br>*Year:* 2017<br>*Category*: A<br>*Country*: UK | To explore patients' expectations of POWeR+, experiences of the POWeR+ programme, experiences of using the POWeR+ website and experiences of nurse support. | Participants with obesity (BMI $\geq$30kg/m$^2$, or $\geq$28kg/m$^2$ with comorbidities) from general practice | *Role:* Participant and Provider<br>*Number of providers*: 13 nurses (HCPs who supported POWeR+ were included in qualitative evaluation)<br>*Number of participants:* 31 POWeR+ programme users. 14 remote support (3 low users/11 high users) and 17 face-to-face support patients (2 low users/15 high users).<br>*Participants' characteristics*: 15 female, 16 male, mean age 61 years (range 45-88 years).<br>*Socioeconomic and demographic characteristics*: No specific data for qualitative analysed participants.<br>*Comorbidities reported*: No specific | This is a 24-session web-based weight management intervention consisting of a series of 24 brief maintenance-oriented sessions for up to 6 months and links to encourage patients to continue to use the website to track their weight at least fortnightly until they have formed healthy eating habits that sustain weight management. | Interviews |

For peer review only

| | | | | | |
|---|---|---|---|---|---|
| | | | data for qualitative analysed participants. | | |
| *First Author*: McRobbie<br>*Year:* 2016<br>*Category*: A<br>*Country*: UK | To explore the many components of the WAP. By providing a summary of participant feedback on the overall helpfulness of the programme. | Adults (aged ≥ 18 years) with obesity (BMI of ≥ 30 kg/m$^2$ or a BMI of ≥ 28 kg/m$^2$ plus comorbidities) who wanted to lose weight | *Role:* Participant<br>*Number of participants:* 177. Participants who reported helpfulness of the programme at 12-months follow up; 48 in the nurse arm and 129 in the WAP arm. People who dropped out of treatment were called; only 19 provided a reason for dropping out.<br>*Participants' characteristics*: Not reported<br>*Socioeconomic and demographic characteristics:* Not reported.<br>*Comorbidities:* Not reported | The WAP is a multicomponent programme that includes a range of concrete and verifiable tasks agreed individually with each participant and also includes monthly 'maintenance' sessions that targeted to improve participant motivation, allowing participants to discuss the challenges they have faced since the last session, and to anticipate challenges of the month ahead. | Anonymous feedback questionnaire |
| *First Author*: Yarborough<br>*Year:* 2016<br>*Category*: A<br>*Country*: USA | To assess lifestyle change barriers and facilitators across the first 18 months of study participation and to identify modifiable factors associated with making and maintaining healthy | Adults (aged ≥ 18 years) with obesity (BMI ≥27kg/m$^2$) taking antipsychotic medications (stable on antipsychotic medications for at least 30 days) | *Role:* Participant<br>*Number of participants*: 84. Participants in the control arm were interviewed once; 17 intervention participants were interviewed more than once to ensure that all cohorts were represented in each interview wave.<br>*Participants' characteristics*: Mean age 48.1 (SD ± 10.1), 30 male, 54 | This was a 24-month study of the STRIDE comprehensive weight loss and lifestyle-change intervention that consisted of 24 weekly meetings that targeted readiness to change; included interactive, participant-centred delivery of lifestyle education information along with a 20-min walk; encouraged skills practice, self-monitoring and feedback; and facilitated group interactions and | Interviews |

For peer review only

| | | | | | |
|---|---|---|---|---|---|
| | lifestyle changes in order to inform clinicians and improve the development of future interventions for individuals with serious mental illnesses. | | female. 18 were members of ethnic or racial minorities.<br>*Socioeconomic and demographic characteristics*: 34 married or living with partner, 27 had an income of $30,000 or higher, 18 were college graduate or higher, 28 were retired, unemployed, student, homemaker or temporarily laid off.<br>*Comorbidities*: 34 Schizophrenia, 17 bipolar disorder, 31 affective psychoses, 2 PTSD | support. Intervention participants could consult with interventionists by telephone as needed. | |
| *First Author*: Abildso<br>*Year:* 2010<br>*Category*: B<br>*Country*: USA | To examine physical and psychosocial differences at baseline between completers of and dropouts from a 12-week weight management program; to assess the physical, behavioural, and psychosocial impact on program completers; to | Adults with obesity (BMI ≥ 30kg/m$^2$ alone or a BMI of 25 to 29.9kg/m$^2$ with comorbidities) | *Role:* Participant<br>*Number of participants*: 11<br>*Participants characteristics*: Mean age 46.2 (SD ± 8.5), 8 female, 3 male. Seven were successful program completers (three high weight losers, four moderate weight losers), and four were program dropouts or completers with low weight loss).<br>*Socioeconomic and demographic characteristics*: 7 married, number of children 1.5 (SD ± 1.1)<br>*Comorbidities*: Not reported | Weight loss is encouraged in the weight management program (WMP) through increasing physical activity and decreasing caloric intake. For a $45 monthly co-payment, the WMP benefit during Phase 1 (12 weeks) included assessment and follow-up meetings with an exercise physiologist and registered dietitian, monthly personal training sessions, and periodic phone calls from the insurance agency to track progress. | Interviews |

For peer review only

| | | | | | |
|---|---|---|---|---|---|
| | compare the psychosocial changes of high and moderate weight losers; and to qualitatively explore factors associated with program adherence and weight loss. | | | | |
| *First Author*: Aschbrenner<br>*Year:* 2016<br>*Category*: B<br>*Country*: USA | To explore participants' perceptions and experiences with peer interactions during the lifestyle intervention. | Obese (BMI ≥ 30kg/m$^2$) adults (aged 21 or older) with serious mental illness (diagnosis of schizophrenia, schizoaffective disorder, major depressive disorder, or bipolar disorder) on stable pharmacological treatment | *Role:* Participant<br>*Number of participants*: 17<br>*Participants' characteristics*: No specific data for qualitative analysed participants<br>*Socioeconomic and demographic characteristics*: Not reported<br>*Comorbidities:* Not reported | A 24-week group-based lifestyle intervention that consisted of once weekly 1-hr group weight management sessions facilitated by a psychologist and a public health professional; twice weekly (optional) 1-hr group exercise sessions led by a certified fitness trainer; and mobile technology and use of social media to increase motivation and facilitate self-monitoring and peer-to-peer support outside of in person group treatment or exercise sessions. | Focus groups |

For peer review only

| | | | | | |
|---|---|---|---|---|---|
| *First Author*: Asselin<br>*Year:* 2015<br>*Category*: B<br>*Country*: Canada | To explore how primary care providers incorporate weight management in their practice. | Obesity prevention and weight management at interdisciplinary primary care environment | *Role:* Provider<br>*Number of providers interviewed:* 29<br>*Providers' characteristics*: 7 mental healthcare workers, 7 registered dietitians, 15 registered nurses or nurse practitioners.<br>*Socioeconomic and demographic characteristics:* NR | The 5 As Team (5AsT) study was designed to create, implement and evaluate a flexible intervention to improve the quality and quantity of weight management visits in primary care. 5AsT is a randomized controlled trial on the implementation of a 6-month 5AsT intervention designed to operationalize the 5As of obesity management in primary care. | Interviews and field notes of intervention sessions |
| *First Author*: Asselin<br>*Year:* 2016<br>*Category*: B<br>*Country*: Canada | To describe the intervention, provide continual intervention monitoring and to identify contextual factors that could influence the primary outcome measure. | See *Asselin 2015* | See *Asselin 2015* | See *Asselin 2015* | See *Asselin 2015* |
| *First Author*: Barham<br>*Year:* 2011<br>*Category*: B<br>*Country*: USA | To improve nutrition and physical activity of county employees and promote weight loss (*There was no* | Adults at highest risk for the development of diabetes or who already have been | *Role:* Participant<br>*Number of participants*: Unclear how many of 45 programme participants provided written responses on the end of study programme evaluations. | There were 2 waves of enrolment and 4 intervention groups (up to 12 participants/ group). The intervention was a 3-month program (12 one hour weekly midday group sessions) that targeted healthy diet, physical activity, | Written responses to end of programme participant evaluations |

For peer review only

| | | | | | |
|---|---|---|---|---|---|
| | *qualitative aim stated).* | diagnosed with type 2 diabetes | *Participants characteristics*: No specific data for those who provided written responses<br>*Socioeconomic and demographic characteristics*: Not reported<br>*Comorbidities reported*: Not reported | and stress reduction, followed by a monthly maintenance program with the groups choosing topics that they considered of greatest benefit. Most of the sessions were led by a nurse educator, but individual sessions were also conducted by a dietitian, psychologist, and physical therapist all employees of Upstate Medical University, Syracuse, NY. | |
| *First Author*: Borkoles<br>*Year:* 2016<br>*Category*: B<br>*Country*: UK | To examine the effects of a non-dieting lifestyle intervention approach for women with morbid obesity designed in the framework of the self-determination theory and Health at Every Size on weight maintenance and psychological functioning. | Pre-menopausal females with morbid obesity (BMI ≥30kg/m$^2$) older than 18 years of age free of obesity-related diseases and fit for exercise | *Role:* Participant<br>*Number of participants*: 62 (62 interviews at baseline with 36 follow-up interviews, including 12 drop-outs).<br>*Participants' characteristics*: Pre-menopausal women predominantly white Caucasian (97%), with a mean age of 40.2 years<br>*Socioeconomic and demographic characteristics*: most were from the lower SES background, 21% had a degree and 57% left school at 16, 66.1% worked full time and 11% worked part-time, in mainly manual | The WHEEL (Weight, Healthy Eating and Exercise in Leeds) study was a delayed-start, 12 weeks of intensive intervention and 40-week maintenance phase RCT comprising of community-based supervised exercise, lifestyle physical activity and psycho-educational classes on healthy eating and weight management. | Interviews |

For peer review only

| | | | | | |
|---|---|---|---|---|---|
| | | | (29%) and administrative jobs (46.8%)<br>*Comorbidities*: 50% met the International Diabetes Federation metabolic syndrome criteria, 42% reported to have depression often or very often, and 36% used medication related to psychological problems | | |
| *First Author*: Dahl<br>*Year:* 2014<br>*Category*: B<br>*Country*: Norway | To describe how personnel argued for and perceived a residential weight-loss program, to investigate how the participants experienced the program, and to contrast these perspectives. | Adults (between 18 and 60 years old) with obesity (BMI > 40kg/m$^2$ or >35kg/m$^2$ including comorbidities)<br>*Providers*:<br>The personnel were recruited among the staff at the centre | *Role:* Participant and Provider<br>*Number of participants*: 10<br>*Participants' characteristics*: 10 Norwegian participants took part in interviews (8 in focus groups and 2 individually). The age and weight range for these 10 persons were the same as for the total sample (n=30). Age between 22 and 56 years old, their BMI was between 40 and 63, and the group's mean body weight was 144kg<br>*Socioeconomic and demographic characteristics*: NR<br>*Comorbidities:* NR<br>*Number of providers interviewed*: 6<br>*Providers' characteristics*: 2 males and 4 females, considered to be key | This 18-week on-site program intervention took place at the Danish residential weight-loss centre. The program consisted of group-based intensive structured group exercise and educational sessions exercise, diet (individual calorie intake was based on energy calculations for a normal weight person with a sedentary activity level), and an educational program. The educational program comprised lessons about nutrition, monitoring of food intake and instruction in behavioural techniques from cognitive therapy. The personal development component included a minimum of two individual conversations with one of the | Focus groups and interviews |

For peer review only

| | | | | | |
|---|---|---|---|---|---|
| | | | personnel; the director, the administrative executive, and the leaders of the main areas diet, exercise and personal development | psychotherapists, motivational meetings for all participants. | |
| *First Author*: Danielsen<br>*Year:* 2016<br>*Category*: B<br>*Country*: Norway | To explore the experiences of physical activity from a participant perspective prior to, during, and after an intensive inpatient lifestyle modification program, including a high volume of adapted physical activity for the treatment of severe obesity. | Both genders, with a variety in age, degree of obesity (BMI ≥ 40 or 35.0–39.9 with comorbidities), and weight loss during the inpatient stay, as well as variation in weight-loss maintenance and lack of maintenance | *Role:* Participant<br>*Number of participants*: 8<br>*Participants' characteristics*: 5 female, 3 male, aged 35 to 63 years; 6 married/cohabitants and 2 single; BMI ranged from 37 to 60 and body weight from 96 to 185 kg<br>*Socioeconomic and demographic characteristics*: NR<br>*Co-morbidities:* NR | The study was supplementary to a clinical controlled trial with a 1-year prospective follow-up study examining the effects of a 10- to 14-week inpatient lifestyle modification program for subjects with severe obesity. Two to three group-exercise sessions 5 days a week during the inpatient period, each lasting for a minimum of 45 minutes. Aiming to increase compliance, the activity was supervised by exercise scientists and physiotherapists, and the participants were introduced to adapted physical activity and equipment, and exercised together with other individuals with severe obesity. | Interviews |
| *First Author*: Groven<br>*Year:* 2010<br>*Category*: B<br>*Country*: Norway | To show how the training is experienced from a first-person perspective, namely | Female participants with obesity (BMI >35kg/m$^2$) from the weight-loss program in | *Role:* Participants<br>*Number of participants*: 5<br>*Participants' characteristics*: Aged 35-63 years and had been overweight for more than 10 years | Group-based weight-loss program in Norway, a program organized by physiotherapists in the primary health system. Offered to eight women struggling with obesity problems in a particular district of Norway for one | Interviews |

For peer review only

| | | | | | |
|---|---|---|---|---|---|
| | the patients themselves. | Norway | *Socioeconomic and demographic characteristics*: 3 married, 1 divorced and 1 widowed, 1 had a university degree, 2 had a college degree, and 2 had no formal education after high school. The women were at present or previously working in professions providing a service, or care, doing office work, or an academic job on various levels.<br>*Comorbidities*: Not reported | year. Total of 12 exercises were performed throughout the one-hour exercise program. The treatment also included group discussion for 1 hour per month. | |
| First Author: Jackson<br>Year: 2007<br>Category: B<br>Country: UK | To evaluate the effectiveness and acceptability of a specialist health visitor-led weight management clinic in primary care. | Patients with a BMI ≥30 | *Role: Participants*<br>*Number of participants: Unclear how many of 25 questionnaires returned provided written responses*<br>*Participants' characteristics: Not reported*<br>*Socioeconomic and demographic characteristics: Not reported*<br>*Comorbidities: Not reported* | Specialist health visitor-led intervention based on the Jan Felgens '12E2' model. The specialist health visitor sought to inspire participants through a combination of shared goal setting, reflection, problem-solving, positive affirmation and reinforcement. Consultations took place at the health centre and a relaxed, unhurried atmosphere was created. The average consultation time was 20 minutes (range 10–30 minutes), although the first appointment took approximately 1 hour and gave participants time to reflect on their lifestyles and to plan realistic goals | Open ended response options to questionnaire |

For peer review only

| | | | | | |
|---|---|---|---|---|---|
| | | | | for healthy eating and physical activity with the specialist health visitor. | |
| *First Author*: Janke<br>*Year:* 2012<br>*Category*: B<br>*Country*: USA | To gain insight into the patient's experience of comorbid chronic pain and obesity and to improve understanding of the behavioural linkages between the experience of pain, engagement in health behaviours, and obesity treatment outcomes. | Patients attending primary care clinics at a large Midwestern Veteran's Affairs hospital, > 18 years, BMI ≥25; weekly pain at an intensity ≥4 during the prior 3 months; and current diagnosis of a medical complaint associated with persistent pain | *Role:* Participant<br>*Number of participants*: 30<br>*Participants characteristics*: 24 male, 6 female<br>26 were age 50 or older, mean BMI was 36.8 (SD ± 8.9)<br>*Socioeconomic and demographic characteristics*: 22 were white, 20 had greater than a high school education, and 14 were unemployed or disabled while 13 were retired<br>*Comorbidities*: Measured on a scale of 0 to 10 (0 = none, 10 = worst imaginable), average pain intensity was 5.6 (SD ± 1.9) and average pain interference was 3.6 (SD ± 2.1) | The qualitative research project was designed to identify perceptions of those with both overweight/obesity and chronic pain regarding their experience of the course, impact, and treatment history of pain and weight symptoms; factors that might either ease or limit their ability to engage in health-promoting behaviours; and factors that facilitate or hinder engagement in treatments designed to achieve weight and/or pain control. | Focus groups and interviews |
| *First Author*: Jennings<br>*Year:* 2014<br>*Category*: B<br>*Country*: UK | To facilitate weight loss by implementing progressive and sustainable lifestyle changes, based on individually agreed goals over a 1-year | Adults (over 18 years) with obesity (BMI ≥40, or BMI ≥30 with obesity-related comorbidities and/or waist | *Role:* Participant<br>*Number of participants*: 12<br>*Participants' characteristics*: No specific data for qualitative analysed participants<br>*Socioeconomic and demographic characteristics*: No specific data for qualitative analysed participants. | The Fakenham weight management service (FWMS) provides Tier 3 services. This paper was service evaluation and had a cohort design recruited patients to a 1-year programme. | Focus groups |

| | | | | | |
|---|---|---|---|---|---|
| | programme. Focus groups were conducted to explore participants' experiences. | circumference ≥102 cm in men or ≥88 cm in women) | *Comorbidities*: No specific data for qualitative analysed participants. | | |
| *First Author*: Jimenez Lopez<br>*Year:* 2012<br>*Category*: B<br>*Country*: Mexico | To explore the motivations of patients involved in a with reduction programme, by analysing their experiences. | Patients with obesity included in a waiting list for bariatric surgery at a public hospital | *Role:* Participant<br>*Number of participants*: 10<br>*Participants' characteristics*: 2 Male, 8 women, mean age 45.2, mean BMI 41.3<br>*Socioeconomic and demographic characteristics*: NR<br>*Comorbidities*: NR | The dynamic of the intervention included the modification of dietary habits by a psychologic intervention, as recommended by the federal law of obesity management The focus group included ten patients with one investigator as an active observer, and 12 weekly sessions. | Focus groups |
| *First Author*: Kidd<br>*Year:* 2013<br>*Category*: B<br>*Country*: USA | To describe the effect of an 8-week mindful eating intervention on mindful eating, weight loss self-efficacy, depression, and biomarkers of weight in urban, underserved, women | Females (aged 30 years and older) with obesity (BMI ≥30kg/m$^2$) | *Role:* Participant<br>*Number of participants*:12<br>*Participants' characteristics*: Mean weight was 119.7kg (SD ± 16.87), BMI 44.7 (SD ±6.9) , Age ranged from 31–61 and averaged 51.8 years (SD ± 9.1)<br>*Socioeconomic and demographic characteristics*: 7 African American, 5 unemployed, and 4 married; 11 | The study used a mixed methods design. A one group pre-test/ post-test design examined the effect of an 8-week mindful eating intervention on the psychosocial variables and biomarkers. Weekly group sessions lasted 60 to 90 minutes and consisted of education and application of mindful eating principles. | Focus groups |

For peer review only

| | | | | | |
|---|---|---|---|---|---|
| | with obesity; and to identify themes of the lived experience of mindful eating. | | graduated from high school, 6 had college degrees<br>*Comorbidities*: Not reported | | |
| *First Author*: Pera<br>*Year:* 2016<br>*Category*: B<br>*Country*: Spain | To explore the meaning of obesity in elderly persons with knee osteoarthritis and to determine the factors that encourage or discourage weight loss. | Participants with obesity, knee osteoarthritis, and polypathology | *Role:* Participant<br>*Number of participants*: 10<br>*Participants characteristics*: 2 male, 8 female, mean age 67.23 (SD ±7.87), BMI 40.47 (SD ± 4.22), mean weight 92.35 kg (SD ± 8.93)<br>*Socioeconomic characteristics*:: 1 No education, 5 Primary (<5 years), 3 Secondary (<10 years), 1 Higher (>10 years), 2 Housewife, 8 Retired<br>*Comorbidities*: Mean number of co-morbidities 7.02 (SD ± 3.08) | The therapeutic education and functional preadaptation program was a 4-month program consisted of two 40-minute individual visits and three 90-minute group sessions for participants with obesity, knee osteoarthritis and polypathology. The program was designed following the methodology established for this type of program and was based on social learning theories. | Focus group |
| *First Author*: Counterweight<br>*Year:* 2008<br>*Category*: B<br>*Country*: UK | To explore key barriers and facilitators of practice and patient engagement in the Counterweight Programme and to describe key strategies used to | Patients with obesity in routine primary care | *Role:* Participant and Provider<br>*Number of participants*: 37 patients<br>*Number of providers*: weight management advisers (n = 7) in a focus group. In depth interviews were conducted with 15 PNs and 7 GPs across 11 practices.<br>*Participants' and/or providers characteristics*: Not reported | The Counterweight Project was set up to establish and improve obesity management in primary care by implementing an evidence-based weight management intervention that is practice focused. It was developed using theoretical models of behavioural change and, the best available methods from the published evidence. | *Participants*:<br>Interviews and focus groups<br><br>*Providers*:<br>Interviews and focus groups |

For peer review only

| | | | | | |
|---|---|---|---|---|---|
| | address barriers in the wider implementation of this weight management programme in UK primary care. | | *Socioeconomic and demographic characteristics*: Not reported<br>*Comorbidities reported*: Not reported | | |
| *First Author*: Shaw<br>*Year:* 2013<br>*Category*: B<br>*Country*: USA | To evaluate the acceptability, feasibility, and efficacy of daily text messages using regulatory focus theory to help individuals sustain weight loss. | Individuals had to own a mobile phone, be able to receive text messages, and have lost 5% of their body weight since entering the Duke Diet and Fitness Centre | *Role:* Participant<br>*Number of participants*: 60<br>*Participants' characteristics*: No specific data for qualitative analysed participants<br>*Socioeconomic and demographic characteristics*: No specific data for qualitative analysed participants.<br>*Comorbidities*: Not reported | Clients who received treatment at a residential weight loss management program that provides education, practical behavioural strategies, and ongoing support to make long-term changes at the Duke Diet and Fitness Centre (DFC), participated in this study. Participants were randomized to a promotion, prevention, or an attention control text message group after completion of a weight loss program. | Interviews |
| *First Author*: Sturgiss<br>*Year:* 2016<br>*Category*: B<br>*Country*: Australia | To describe the collaborative process used to develop an obesity management programme based on current Australian guidelines for GPs and their patients to | Health professionals involved in obesity management programme based on current Australian | *Role:* Provider<br>*Number of providers*: 38<br>*Providers' characteristics*: 15 GPs, 14 GPs registrar, 5 healthcare consumer representative, 2 representative bodies for chronic illness, 1 dietician, 1 psychologist | The Change Programme is a GP-delivered weight management programme that was developed based on Australian guidelines for the management of obesity in primary healthcare. It is based on one of the pillars of general practice—'patient centeredness'. No directive patient goals | Interviews and focus groups |

For peer review only

| | | | | | |
|---|---|---|---|---|---|
| | be used in primary care. | guidelines for GPs and their patients to be used in primary care | *Socioeconomic and demographic characteristics*: Not reported | were stated and the work was individualized. The programme consists of a GP handbook, patient workbook and computer template. This programme. The patients initially attended appointments every 2 weeks, with less frequent appointments as the programme continued. | |
| *First Author*: Sturgiss<br>*Year:* 2017<br>*Category*: B<br>*Country*: Australia | To assess the acceptability and feasibility of a GP-delivered weight management programme. | *Providers*: Fully qualified GPs from the Australian Capital Territory and New South Wales. | *Role:* Participant and Provider<br>*Number of providers*: 12<br>*Providers' characteristics*: The recruited GPs had an average 12 years of experience (range 4–30 years). The GPs worked in four urban practices and one rural practice.<br>*Number of patient participants*: 15 interviewed<br>*Participants' characteristics*: No specific data for qualitative analysed participants.<br>*Socioeconomic and demographic characteristics*: NR<br>*Comorbidities*: Not reported | See *Sturgiss 2016a* | Interviews |
| *First Author*: Sturgiss<br>*Year:* 2017<br>*Category*: B | To assess the self-efficacy and confidence of GPs | GPs working in 5 different general practices | *Role:* Provider<br>*Number of providers*: 12 | See *Sturgiss 2016a* | Interviews |

For peer review only

| | | | | | |
|---|---|---|---|---|---|
| *Country*: Australia | before and after implementing a weight management programme in their practice. | | *Providers' characteristics*: 12 GPs practised in 5 different general practices, 1 rural and 4 urban, and had between 4 and 30 years clinical experience<br>*Socioeconomic and demographic characteristics*: Not reported | | |
| *First Author*: Turner<br>*Year:* 2015<br>*Category*: B<br>*Country*: UK | To determine both physiological benefits and qualitative information, namely patient satisfaction, associated with the service. | Patients with obesity attending Multidisciplinary Weight Management Clinic (MDWMC) at Aneurin Bevan Hospital, Wales | *Role:* Participant<br>*Number of participants*: 180<br>*Participants characteristics*: 131 female, 49 male, ages ranged between 19 and 74<br>*Socioeconomic and demographic characteristics*: Not reported<br>*Comorbidities*: Not reported | Obesity management in Wales includes the provision of a 1:1 MDWMC. Strategic management of obesity in Wales is guided by The All Wales Obesity Pathway and recommends MDWMCs for people with obesity who have one or more co morbidities and who have tried several interventions without success, or who have complex emotional relationships with food. | Interviews |
| *First Author*: VanWormer<br>*Year:* 2010<br>*Category*: B<br>*Country*: USA | To examine the association between participant and program experiences and satisfaction with a weight loss intervention. | Adults (18 years or older) with obesity (BMI ≥ 32kg/m$^2$) employees of a managed care organization | *Role:* Participant<br>*Number of participants*: 78 (*not clear if all of these provided qualitative information*)<br>*Participants' characteristics*: Mean age 46.9 (SD ± 8.3), 70 female, 8 male, 55 married or living with a partner, 23 not married; body weight | Participants were randomly assigned to either an immediate or delayed start group. The intervention lasted 6 months. During treatment, participants received a telephone-based behavioural weight loss counselling intervention. The intervention included a course manual, behaviour change tools (e.g., food/ activity log, weight chart, pedometer), | Written responses to open ended response options within a questionnaire |

| | | | | | |
|---|---|---|---|---|---|
| | | | (kg) 106.2 (SD ± 16.32), BMI 38.3 (SD ± 5.2)<br>*Socioeconomic and demographic characteristics*: 36 college or graduate degree, 42 had less than college degree<br>*Comorbidities*: Not reported | and up to 10 telephone counselling calls from a registered dietitian and/or health educator. In addition, participants received a home tele monitoring scale and were instructed to weigh themselves daily. | |
| *First Author*: Young<br>*Year:* 2017<br>*Category*: B<br>*Country*: USA | To determine whether computerized provision of weight management with peer coaching is feasible to deliver, is acceptable to patients, and is more effective than in-person delivery or usual care. | Adults (18 years or older) with obesity (BMI > 30 or 28–30kg/$m^2$ with self-reported weight gain of at least 10 pounds in the last 3 months), with diagnosis of schizophrenia, schizoaffective disorder, bipolar disorder, major depressive disorder with psychosis, or posttraumatic | *Role:* Participant<br>*Number of participants*: 48 (24 randomized to WebMOVE and 24 randomized to MOVE SMI)<br>*Participants' characteristics*: No specific data for qualitative analysed participants<br>*Socioeconomic and demographic characteristics*: No specific data for qualitative analysed participants<br>*Comorbidities*: Not reported | Patients were randomized to a computerized weight management with peer coaching (Web- MOVE) or in-person clinician-led weight services, or usual care. Both active interventions offered the same educational content. WebMOVE weekly manualized peer coaching was delivered by phone and emphasized a strengths-based approach with motivational interviewing. MOVE SMI is an in-person weight management program led by a master's level mental health clinician. The program includes 24 sessions (8 individual and 16 group), each lasting 60 min. Usual care consisted of one educational handout on the benefits of weight loss, given to participants after randomization | Interviews |

For peer review only

| | | | | | |
|---|---|---|---|---|---|
| | | stress disorder; with prescribed an antipsychotic medication | | | |
| *First Author*: Zizzi<br>*Year:* 2016<br>*Category*: B<br>*Country*: USA | To explain how these services are perceived and received by participants in a community-based intervention so that specific recommendations can be made to health professionals working with similar populations and in similar settings. | West Virginia public employees' insurance agency weight management program (WMP), which is open to insured members that have a BMI >25 | *Role:* Participant<br>*Number of participants*: 567 (not clear how many provided qualitative data within the questionnaire<br>*Participants' characteristics*: 437 female, 130 male<br>*Socioeconomic and demographic characteristics*: Not reported<br>*Comorbidities*: Self-reported medication usage for 36% heart disease or high blood pressure, 31% anxiety or depression 21% high cholesterol, 12.7% diabetes, 9% sleep apnea | The WMP was a 2-year long benefit, and a $20 monthly co-payment that allowed participants to meet with a registered dietitian, exercise physiologist, and certified personal trainer at various point throughout their time in the program. The majority of individuals in the program also spoke with a health behaviour counsellor via telephone every 6 to 8 weeks. The WMP was offered at approximately 60 approved exercise facilities in West Virginia, such as YMCAs, wellness centres, fitness centres, and physical therapy clinics. | Written responses to open ended response options within a questionnaire |
| *First Author*: Owen Smith<br>*Year:* 2014<br>*Category*: C<br>*Country*: UK | To present a synthesis of data from two qualitative studies in which both the development and the experience of living with morbid obesity in men and | Individuals who met the United Kingdom NICE criteria for a morbid obesity (BMI $\geq$ 40, or 35 kg/m$^2$ with comorbidity), and | *Role:* Participant<br>*Number of participants*: 31 (Study 1 n = 13; Study 2 n = 18)<br>*Participants characteristics*: 9 males, 3 age group 20–29, 11 age group 30–39, 7 age group 40–49, 9 age group 50–59, 1 60+ age group | The qualitative approach to both studies, to investigate individual experiences of developing and living with morbid obesity. The first study (Study 1) as part of a broader investigation into patients' experiences of implicit and explicit rationing. The core results the second study (Study 2) as part of an ongoing | Interviews |

For peer review only

| | | | | | |
|---|---|---|---|---|---|
| | women were explored in depth. | sought access to treatment for their condition | *Socioeconomic and demographic characteristics*: 15 non manual employment, 5 manual employment, 5 homeworker/carer, 1 retired, 4 unemployed<br>*Comorbidities*: Not reported | longitudinal study investigating how clinicians communicate with patients about the availability of treatment in the context of resource scarcity. | |
| *First Author*: Owen Smith<br>*Year:* 2016<br>*Category*: C<br>*Country*: UK | To focus on experiences of accessing treatment for morbid obesity in primary care. | Patients and providers at a weight management clinic at a general hospital in the South West of England | *Role:* Participant and providers<br>*Number of participants*: 22 patients<br>*Number of providers*: 11<br>*Participants' characteristics*: 7 male, 15 female, 9 age group 20-39, 12 age group 40-59, 1 age 60+<br>*Socioeconomic and demographic characteristics*: 21 white British, 4 professional, 8 other non-manual, 3 manual, 6 unemployed, 1 retired<br>*Comorbidities*: 19 joint pain/mobility issues, 11 depression/other depressive disorder, 10 breathlessness/respiratory difficulties, 9 diabetes, 8 hypertension, 4 sleep apnoea, 4 cardiac problems, 3 fertility issues<br>*Number of providers*: 11 clinicians<br>*Providers' characteristics*: Clinician informants included consultants and | Data collection was undertaken using in-depth interviews with patients and clinicians working in a specialist secondary care facility, and analysis took a constant comparative approach. Patients were followed from before their first consultation in secondary care up to 36 months after referral. | Interviews |

For peer review only

| | | | | | |
|---|---|---|---|---|---|
| | | | three allied medical professionals who worked within the weight management service.<br>*Socioeconomic and demographic characteristics*: Not Reported | | |

Categories: A= Qualitative and mixed-methods studies linked to eligible RCTs, including any qualitative data reported as part of papers reporting quantitative outcomes; B= Qualitative and mixed-methods studies linked to ineligible RCTs and identified non-randomised intervention studies including any reported qualitative data; C= UK-based qualitative studies not linked to any specific interventions that draw on the experiences and perceptions of adults with BMI ≥35 (and/or providers involved in their care). ¥=Studies included in review 2 (long-term randomised and non-randomised studies conducted in UK). BMI= Body Mass Index, calculated weight (kg) / height (m2)

For peer review only

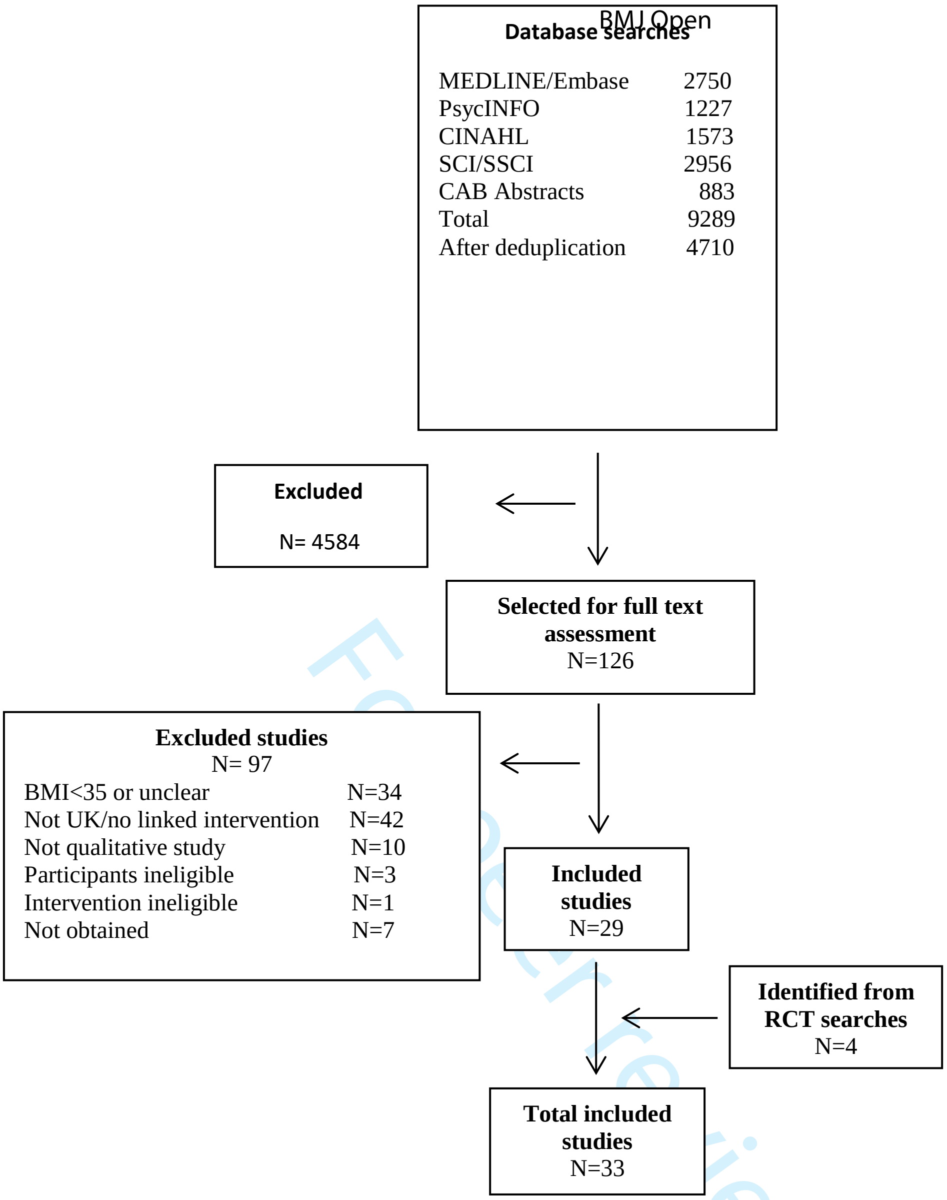


**S1 Figure Flow chart of included studies**

For peer review only

**REVIEW: Qualitative Studies**

**MEDLINE and EMBASE**

Ovid multifile search: http://shibboleth.ovid.com/

Database: Embase <1980 to 2017 Week 31>, Epub Ahead of Print, In-Process & Other Non-Indexed Citations, Ovid MEDLINE(R) Daily and Ovid MEDLINE(R) <1946 to Present>

26th April 2017

**Date of Search 26th April 2017**

1 qualitative research/

2 exp interviews as topic/ use ppez

3 exp interview/ use emez

4 focus groups/ use ppez

5 grounded theory/

6 (qualitative or interview$ or focus group?).tw,kw.

7 (ethno$ or grounded or thematic or realist or interpretive or narrative or discourse analysis or discursive or mixed method$).tw,kw.

8 or/1-7

9 *obesity/

10 morbid obesity/ use emez

11 exp obesity, morbid/ use ppez

12 (obese or obesity).tw,kw

13 or/9-12

14 Weight Loss/ use ppez

15 weight reduction/ use emez

16 (weight adj1 (los$ or reduc$ or maint$ or control$ or manag$)).tw,kw.

17 (reduc$ adj2 (bmi or body mass index)).tw.

18 (reduc$ adj2 (waist adj3 (ratio$ or circumference))).tw.

19 (obesity adj1 manag$).tw,kw

20 anti obesity.tw,kw

21 or/14-20

For peer review only

22 8 and 13 and 21
23 (obes$ adj3 (morbid$ or severe$ or extreme$)).tw,kw.
24 8 and (10 or 11 or 23)
25 22 or 24
26 25 not (abstract or letter or note or comment).pt.
27 remove duplicates from 26

**PsycINFO**

Ovid: http://shibboleth.ovid.com/

Database: PsycINFO <1987 to April Week 3 2017>

**Date of Search: 26th April 2017**

1 qualitative research/
2 interviews/
3 grounded theory/
4 discourse analysis/
5 ethnography/
6 (qualitative or interview$ or focus group?).tw,kw.
7 (ethno$ or grounded or thematic or realist or interpretive or narrative or discourse analysis or discursive or mixed method$).tw,kw.
8 or/1-7
9 obesity/ or body weight/
10 (obese or obesity).tw,kw
11 9 or 10
12 Weight Loss/ or weight control/
13 (weight adj1 (los$ or reduc$ or maint$ or control$ or manag$)).tw,kw.
14 (reduc$ adj2 (bmi or body mass index)).tw.
15 (reduc$ adj2 (waist adj3 (ratio$ or circumference))).tw
16 anti obesity.tw,kw.
17 (obesity adj1 manag$).tw,kw
18 or/12-17
19 8 and 11 and 18
20 (obes$ adj3 (morbid$ or severe$ or extreme$)).tw,kw.

For peer review only

21 8 and 20

22 "obesity (attitudes toward)"/

23 19 or 21 or 22

**CINAHL**

http://search.ebscohost.com

!981- 25th April 2017

**Date of Search: 25th April 2017**

For peer review only

S1 (MH "Qualitative Studies+")

S2 (MH "Interviews") OR (MH "Semi-Structured Interview") OR (MH "Structured Interview")

S3 (MH "Focus Groups")

S4 (MH "Narratives")

S5 TX qualitative OR TX interview* OR TX focus group*

S6 TX ( ethno* or grounded or thematic ) OR TX ( realist or interpretive or narrative ) OR TX ( discourse analysis or discursive or mixed method* )

S7 S1 OR S2 OR S3 OR S4 OR S5 OR S6

S8 (MH "Obesity") OR (MH "Obesity, Morbid")

S9 (MH "Body Weight")

S10 TX obese OR TX obesity

S11 S8 OR S9 OR S10

S12 (MH "Weight Control")

S13 (MH "Weight Loss")

S14 TX weight N1 los* OR TX weight N1 reduc* OR TX weight N1 maint* OR TX weight N1 control

S15 TX weight N1 manag* OR TX reduc* N2 bmi OR TX reduc* N2 body mass

S16 reduc* N2 waist ratio* OR TX reduc* N2 waist circumference TX

S17 S12 OR S13 OR S14 OR S15 OR S16

S18 (S7 AND S11 AND S17)

S19 (MH "Obesity, Morbid")

S20 TX obes* N3 morbid* OR TX obes* N3 severe OR TX obes* N3 extreme*

S21 S19 OR S20

S22  S7 AND S21

S23 (MH "Attitude to Obesity")

S24  S18 OR S22 OR S23

**Science Citation Index and Social Science Citation Index**

www.webofknowledge.com

1980 -  28th April 2017

**Date of Search: 28th April 2017**

# 1  TS=(qualitative or interview* or focus group)

# 2  TS=(ethno* or grounded or thematic or realist or interpretive or narrative or discourse analysis or discursive or mixed method*).

# 3  #1 OR #2

# 4  TS=(obesity or obese)

# 5   TS=(weight NEAR/1 los*) or TS=(weight NEAR/1 reduc*) or TS=(weight NEAR/1 maint*) or TS=(weight NEAR/1 control*) or TS=(weight NEAR/1 manag*).

# 6  TS=(reduc* NEAR/2 BMI) OR TS=(reduc* NEAR/2 body mass index)

# 7  TS=anti obesity

# 8  TS= (obesity NEAR/1 manag*)

# 9  #5 or #6 or #7 or #8

10  #3 AND #4 AND #9  *))) AND DOCUMENT TYPES: (Article)

**CAB Abstracts**

Ovid search: http://shibboleth.ovid.com/

Database: CAB Abstracts <1984 to 2017 Week 15>

**Date of Search: 26th April 2017**

1     qualitative analysis/

2     qualitative techniques/

3     (qualitative or interview$ or focus group?).tw.

4     (ethno$ or grounded or thematic or realist or interpretive or narrative or discourse analysis or discursive or mixed method$).tw.

5     or/1-4

6 obesity/

7 (obese or obesity).tw.

8 6 or 7

9 weight reduction/

10 (weight adj1 (los$ or reduc$ or maint$ or control$ or manag$)).tw.

11 (reduc$ adj2 (bmi or body mass index)).tw.

12 (reduc$ adj2 (waist adj3 (ratio$ or circumference))).tw.

13 (obesity adj1 manag$).tw

14 anti obesity.tw.

15 or/9-14

16 5 and 8 and 15

17 (obes$ adj3 (morbid$ or severe$ or extreme$)).tw.

18 5 and 17

19 16 or 18

For peer review only

# Enhancing transparency in reporting the synthesis of qualitative research: ENTREQ

**ENTREQ Statement: content and rationale**

The ENTREQ statement consists of 21 items grouped into five main domains: introduction, methods and methodology, literature search and selection, appraisal, and synthesis of findings (Table 1). For each item, a descriptor and examples are provided. Below we present a rationale for each domain and its associated items.

Table 1

**Enhancing transparency in reporting the synthesis of qualitative research: the ENTREQ statement**

| No | Item | Guide and description | |
|---|---|---|---|
| **1** | Aim | State the research question the synthesis addresses. | See Page 3 |
| **2** | Synthesis methodology | Identify the synthesis methodology or theoretical framework which underpins the synthesis, and describe the rationale for choice of methodology *(e.g. meta-ethnography, thematic synthesis, critical interpretive synthesis, grounded theory synthesis, realist synthesis, meta-aggregation, meta-study, framework synthesis).* | See Page 4 |
| **3** | Approach to searching | Indicate whether the search was pre-planned (*comprehensive search strategies to seek all available studies)* or iterative (*to seek all available concepts until they theoretical saturation is achieved)*. | See Page 3/4 |
| **4** | Inclusion criteria | Specify the inclusion/exclusion criteria *(e.g. in terms of population, language, year limits, type of publication, study type).* | See Page 3 |
| **5** | Data sources | Describe the information sources used (e.g. *electronic databases (MEDLINE, EMBASE, CINAHL, psycINFO, Econlit), grey literature databases (digital thesis, policy reports), relevant organisational websites,* | See Page 3 |

| No | Item | Guide and description | |
|---|---|---|---|
| | | *experts, information specialists, generic web searches (Google Scholar) hand searching, reference lists)* and when the searches conducted; provide the rationale for using the data sources. | |
| **6** | Electronic Search strategy | Describe the literature search *(e.g. provide electronic search strategies with population terms, clinical or health topic terms, experiential or social phenomena related terms, filters for qualitative research, and search limits)*. | See Page 3 and S1 Appendix |
| **7** | Study screening methods | Describe the process of study screening and sifting *(e.g. title, abstract and full text review, number of independent reviewers who screened studies).* | See Page 3/4 |
| **8** | Study characteristics | Present the characteristics of the included studies *(e.g. year of publication, country, population, number of participants, data collection, methodology, analysis, research questions).* | See Page 6/7 and S1 Table |
| **9** | Study selection results | Identify the number of studies screened and provide reasons for study exclusion *(e,g, for comprehensive searching, provide numbers of studies screened and reasons for exclusion indicated in a figure/flowchart; for iterative searching describe reasons for study exclusion and inclusion based on modifications t the research question and/or contribution to theory development).* | See Figure 1, page 5 |
| **10** | Rationale for appraisal | Describe the rationale and approach used to appraise the included studies or selected findings *(e.g. assessment of conduct (validity and robustness), assessment of reporting (transparency), assessment of content and utility of the findings).* | See Page 5 |

| No | Item | Guide and description | |
|---|---|---|---|
| **11** | Appraisal items | State the tools, frameworks and criteria used to appraise the studies or selected findings *(e.g. Existing tools: CASP, QARI, COREQ, Mays and Pope* [25]*; reviewer developed tools; describe the domains assessed: research team, study design, data analysis and interpretations, reporting).* | See Page 5 |
| **12** | Appraisal process | Indicate whether the appraisal was conducted independently by more than one reviewer and if consensus was required. | See Page 5. Two reviewers initially assessed quality of included studies using the criteria proposed by Toye et al. During subsequent group discussions we continued to discuss and reflect on key aspects of quality. |
| **13** | Appraisal results | Present results of the quality assessment and indicate which articles, if any, were weighted/excluded based on the assessment and give the rationale. | Please see detail provided on pages 22-23 |
| **14** | Data extraction | Indicate which sections of the primary studies were analysed and how were the data extracted from the primary studies? *(e.g. all text under the headings "results /conclusions" were extracted electronically and entered into a computer software).* | See Page 4 ans S1 Table |
| **15** | Software | State the computer software used, if any. | N/A |
| **16** | Number of reviewers | Identify who was involved in coding and analysis. | See Pages 4 |
| **17** | Coding | Describe the process for coding of data *(e.g. line by line coding to search for concepts).* | See Page 4 |
| **18** | Study comparison | Describe how were comparisons made within and across studies *(e.g. subsequent studies were coded into pre-existing concepts, and new concepts were created when deemed necessary).* | See Page 4 and S1 Table |

| No | Item | Guide and description | |
|---|---|---|---|
| **19** | Derivation of themes | Explain whether the process of deriving the themes or constructs was inductive or deductive. | See page 4 |
| **20** | Quotations | Provide quotations from the primary studies to illustrate themes/constructs, and identify whether the quotations were participant quotations of the author's interpretation. | See Results section |
| **21** | Synthesis output | Present rich, compelling and useful results that go beyond a summary of the primary studies (e.g. *new interpretation, models of evidence, conceptual models, analytical framework, development of a new theory or construct).* | See Results and discussion section. |

For peer review only

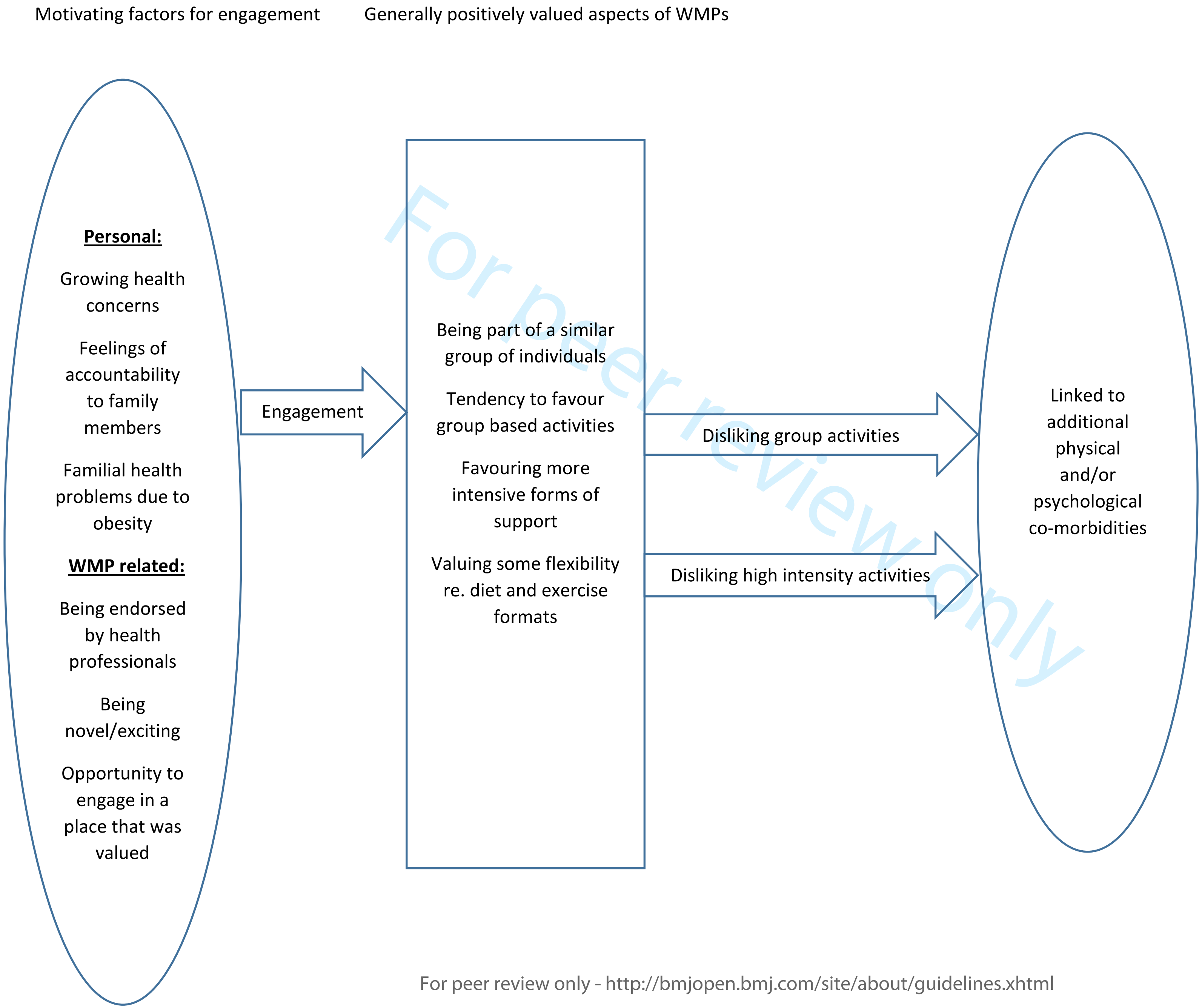


For peer review only
